# Endothelial Foxo1 Phosphorylation Inhibition via Aptamer‐Liposome Alleviates OPN‐Induced Pathological Vascular Remodeling Following Spinal Cord Injury

**DOI:** 10.1002/advs.202406398

**Published:** 2024-09-28

**Authors:** Jiaqi Xu, Chaoran Shi, Yinghe Ding, Tian Qin, Chengjun Li, Feifei Yuan, Yudong Liu, Yong Xie, Yiming Qin, Yong Cao, Tianding Wu, Chunyue Duan, Hongbin Lu, Jianzhong Hu, Liyuan Jiang

**Affiliations:** ^1^ Department of Spine Surgery and Orthopaedics Xiangya Hospital Central South University Changsha 410008 China; ^2^ Key Laboratory of Organ Injury Aging and Regenerative Medicine of Hunan Province Changsha 410008 China; ^3^ Department of Neurosurgery Xiangya Hospital Central South University Changsha 410008 China; ^4^ National Clinical Research Center for Geriatric Disorders Xiangya Hospital Central South University Changsha 410008 China; ^5^ Department of Sports Medicine Xiangya Hospital Central South University Changsha 410008 China

**Keywords:** foxo1, osteopontin, spinal cord injury, targeted therapy, vascular remodeling

## Abstract

Reconstruction of the neurovascular unit is essential for the repair of spinal cord injury (SCI). Nonetheless, detailed documentation of specific vascular changes following SCI and targeted interventions for vascular treatment remains limited. This study demonstrates that traumatic pathological vascular remodeling occurs during the chronic phase of injury, characterized by enlarged vessel diameter, disruption of blood‐spinal cord barrier, endothelial‐to‐mesenchymal transition (EndoMT), and heightened extracellular matrix deposition. After SCI, osteopontin (OPN), a critical factor secreted by immune cells, is indispensable for early vascular regeneration but also contributes to traumatic pathological vascular remodeling. This work further elucidates the mechanism by which OPN influences spinal cord microvascular endothelial cells, involving Akt‐mediated Foxo1 phosphorylation. This process facilitates the extranuclear transport of Foxo1 and decreases Smad7 expression, leading to excessive activation of the TGF‐β signaling pathway, which ultimately results in EndoMT and fibrosis. Targeted inhibition of Foxo1 phosphorylation through an endothelium‐specific aptamer‐liposome small molecule delivery system significantly mitigates vascular remodeling, thereby enhancing axon regeneration and neurological function recovery following SCI. The findings offer a novel perspective for drug therapies aimed at specifically targeting pathological vasculature after SCI.

## Graphical abstract

Osteopontin (OPN) promotes pathological vascular remodeling after spinal cord injury (SCI). OPN activates Akt‐mediated Foxo1 phosphorylation in spinal cord microvascular endothelial cells, which reduces Smad7 expression and excessively activates TGF‐β signaling, leading to endothelial‐to‐mesenchymal transition and fibrosis. Inhibition of Foxo1 phosphorylation via aptamer‐liposome encapsulated Sarmentosin reduces vascular remodeling and enhances neurological recovery post‐SCI.

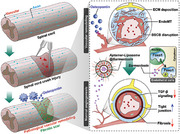

## 1 Introduction

Spinal cord injury (SCI) constitutes a serious impairment of the central nervous system, frequently leading to considerable disability among affected individuals. Worldwide, more than 6.2 million individuals suffer from SCI, with a predominant effect on young and middle‐aged adults, which is crucial for workforce efficiency.^[^ ^1^ ^]^ Due to the absence of effective clinical interventions to improve neurological function in SCI patients, innovative pharmaceutical strategies remain necessary.^[^ ^2^ ^]^ A significant obstacle to progress is the insufficient comprehension of the inherent repair mechanisms following SCI.^[^ ^3^ ^]^

Although advancements have been achieved in directly promoting axon regeneration or neuron survival, challenges remain in securing long‐term effective functional recovery.^[^ ^4^ ^]^ A primary issue is the inability to fully restore the neurovascular unit.^[^ ^5^ ^]^ The disruption of vascular structures, a key pathological feature oftraumatic SCI, results in tissue ischemia and hypoxia, which subsequently triggers blood‐spinal cord barrier (BSCB) disruption, immune cell activation, and the accumulation of neurotoxic substances.^[^ ^6^ ^]^ These pathophysiological alterations create a detrimental environment that persistently obstructs axon regeneration and neural circuit reconstruction. To address this challenge, vascular reconstruction has become a crucial factor in promoting neural tissue repair following SCI.

Endogenous angiogenesis triggered by hypoxia and proangiogenic growth factors can partially restore blood supply and mitigate ischemia.^[^ ^7^ ^]^ Although promoting early angiogenesis within the spinal cord through external modulation can somewhat improve tissue repair in SCI models, neurological recovery frequently reaches a plateau.^[^ ^4^, ^8^ ^]^ Blood vessel density temporarily increases but typically remains leaky with disrupted BSCB, proving insufficient to support local metabolism and potentially accelerating cell death at lesion sites.^[^ ^7^, ^9^ ^]^ The complexities of ongoing microvascular remodeling after SCI remain largely unexplored. In the chronic phase of SCI, persistent BSCB impairment,^[^ ^10^ ^]^ an imbalanced microenvironment,^[^ ^11^ ^]^ and ongoing inflammatory responses^[^ ^12^ ^]^ continue at the injury site. The formation of dense fibrotic scars, characterized by excess extracellular matrix (ECM) deposition and the failure of glial cells and axons to regenerate, underscore the incomplete functionality of the newly formed vascular network.^[^ ^13^ ^]^ Research has shown that endothelial‐to‐mesenchymal transition (EndoMT) and endothelial fibrosis may occur within the injury epicenter.^[^ ^14^ ^]^ However, there is still a lack of evidence regarding the phenotypic characterization of endothelial cells during this process. This indicates a critical gap in understanding the pathological alterations and influencing factors in vascular remodeling.

Osteopontin (OPN)/ secreted phosphoprotein‐1 (Spp1) is a secreted protein known to play crucial roles in the tissue repair process,^[^ ^15^ ^]^ neuroinflammation,^[^ ^16^ ^]^ and neurological repair.^[^ ^17^ ^]^ Its expression markedly increases in immune cells after SCI, indicating a potentially significant role in spinal cord repair.^[^ ^18^ ^]^ Recent research has uncovered the complex involvement of OPN in vascular remodeling.^[^ ^19^ ^]^ Nevertheless, the role of OPN in traumatic pathological vascular remodeling after SCI remains elusive. Understanding the relationship between OPN and endothelial cells is crucial, as it may guide targeted pharmaceutical approaches and facilitate neural repair following SCI.

OPN exerts its effects by binding to cell surface integrin receptors and activating the downstream PI3K‐Akt signaling pathway, which is critical for the endothelial cell‐matrix adhesion and signal crosstalk.^[^ ^20^ ^]^ During mesenchymal and fibrotic transformation in endothelial cells, the activation of the transforming growth factor‐β (TGF‐β) signaling pathway serves as a key downstream executor.^[^ ^21^ ^]^ The mechanism by which OPN affects the phenotype of endothelial cells remains unclear. Elucidating these mechanisms will enhance the understanding of microenvironment factors and aid in the identification of new targets for pharmaceutical therapy.

Targeted modulation of the endothelial phenotype in vivo poses considerable challenges. Conventional methods that use protein ligands for endothelial cell binding depend on genetically engineered cell protein expression, which entails substantial costs and intricate procedures.^[^ ^22^ ^]^ Nanocarriers, known for their high biocompatibility, can facilitate precise drug delivery.^[^ ^23^ ^]^ Among these, liposomes are advantageous as drug carriers due to their favorable cellular uptake and substantial modifiability, rendering them an effective and safe option for in vivo drug delivery.^[^ ^24^ ^]^ The direct modification of endothelium‐specific aptamers on liposomes holds promise for achieving efficient vascular targeted therapy after SCI.^[^ ^25^ ^]^

In this study, we first characterized the late‐stage pathological vascular remodeling following mouse spinal cord crush injury. Next, by combining bioinformatics analysis with data from OPN knockout mice, we identified pivotal functions of OPN in vascular regeneration and traumatic pathological vascular remodeling after SCI. Subsequently, in vitro experiments with spinal cord microvascular endothelial cells (SCMECs) showed that OPN overactivated the TGF‐β signaling pathway in microvascular endothelial cells through a targetable Foxo1‐Smad7 axis, resulting in EndoMT and fibrosis. Based on these new findings and mechanisms, an aptamer‐liposome‐encapsulated small molecule was developed for vascular therapy. This drug delivery system is designed to specifically target endothelial cells at the injury epicenter of SCI. This approach has the potential to significantly advance SCI treatment by ameliorating pathological vascular remodeling, restoring BSCB functionality, reducing fibrotic scar formation, and facilitating neurological function recovery.

## 2 Results

### 2.1 Pathological Vascular Remodeling after Spinal Cord Injury

**Figure 1:**
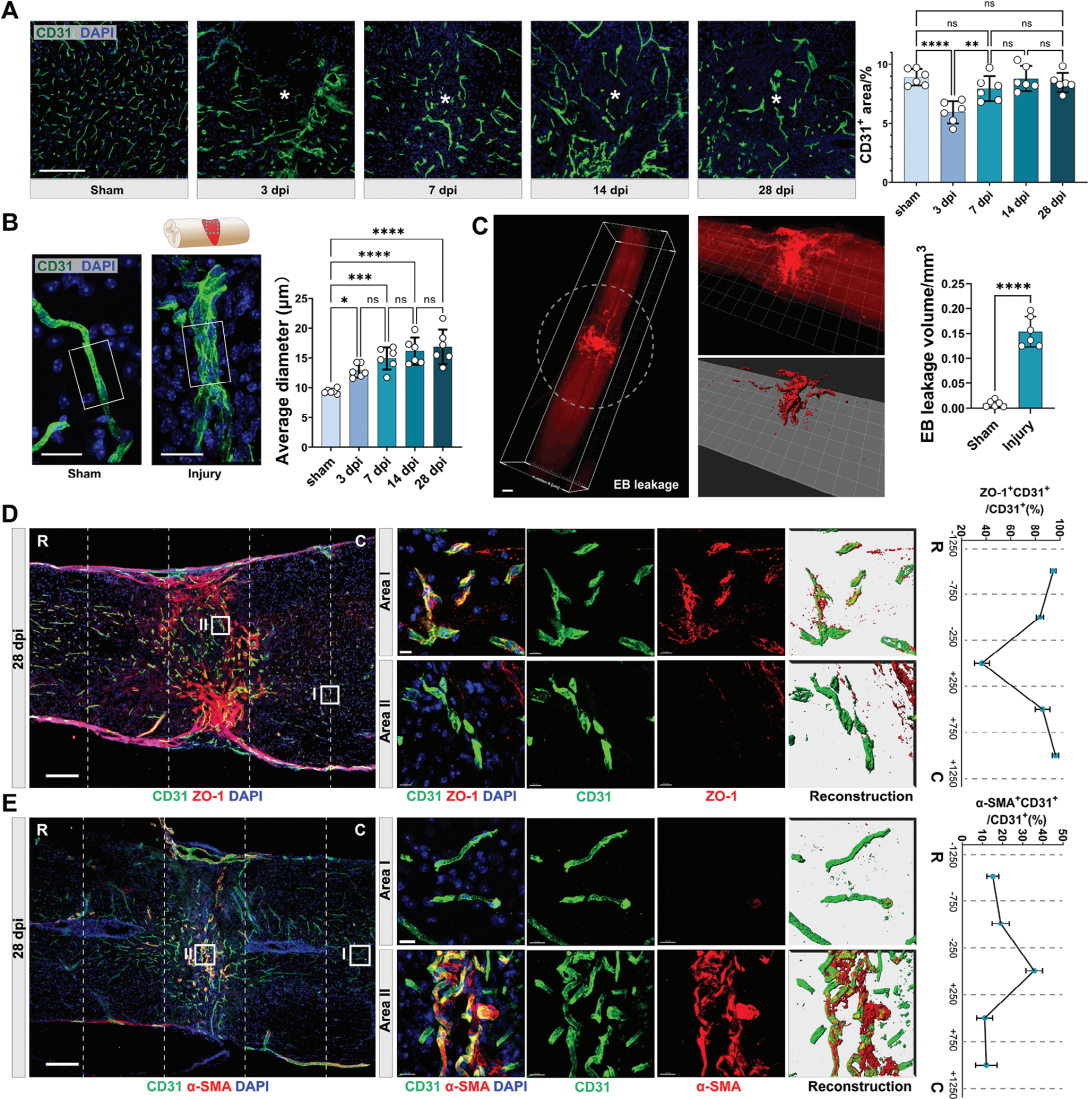
Pathological vascular remodeling in the chronic phase of SCI. A) Representative IF images of CD31 (green) and DAPI (blue), and quantitative analysis of CD31^+^ area (%) in the sham or epicenter of the injured spinal cord at 3, 7, 14, and 28 dpi. (Scale bar = 200 µm, * denotes epicenter, n = 6, mean ± SD, one‐way ANOVA, Tukey's multiple comparisons) B) Representative confocal images of CD31 (green) and DAPI (blue) in the sham or injured spinal cord (images captured from the epicenter as illustrated), and quantitative analysis of average diameters of CD31^+^ vessels in the sham or injured spinal cord at 3, 7, 14, and 28 dpi. (Scale bar = 20 µm, white box = 20 × 30 µm, n = 6, mean ± SD, one‐way ANOVA, Tukey's multiple comparisons) C) Light sheet‐microscope 3D images of the spinal cord after Evans blue (EB) leakage (red) assay (Scale bar = 500 µm). White dashed circles outline the epicenter. The quantification of the EB^+^ volume in spinal cord sections is presented (right). (n = 6, mean ± SD, unpaired t‐test) D) Representative confocal images of CD31 (green), ZO‐1 (red), and DAPI (blue) in the injured spinal cord at 28 dpi (n = 3, Scale bar = 200 µm, R rostral, C caudal), and the zoom‐in view of local vessels in Area I (injury epicenter) and Area II (uninjured area) (Scale bar = 10 µm). (Right panel: quantification of ZO‐1^+^CD31^+^/total CD31^+^ area (%), n = 3, mean ± SD, one‐way ANOVA, Tukey's multiple comparisons) E) Representative confocal images of CD31 (green), α‐SMA (red), and DAPI (blue) in the injured spinal cord at 28 dpi (n = 3, Scale bar = 200 µm), and the zoom‐in view of local vessels in Area I (injury epicenter) and Area II (uninjured area) (Scale bar = 15 µm). (Right panel: quantification of α‐SMA^+^CD31^+^/total CD31^+^ area (%) in spinal cord segments. (n = 3, mean ± SD, one‐way ANOVA, Tukey's multiple comparisons) ns not significant, ∗ p < 0.05, ∗∗ p < 0.01, ∗∗∗ p < 0.001, ∗∗∗∗ p < 0.0001.

Immunofluorescence (IF) images revealed that on 3 days post‐injury (dpi), the CD31^+^ vascular structure was disrupted, with a significant decrease in CD31^+^ areas. However, by 7, 14, and 28 dpi, the CD31^+^ areas had returned to levels comparable to the sham group (**Figure** **1**A), suggesting the presence of endogenous angiogenesis following SCI, peaking at 14 dpi. However, the regenerated vessels at the injury site had larger diameters, with a continued increase in the vessel diameter post‐injury from 3 to 28 dpi (Figure 1B). Additionally, the most highly expressed component of the ECM within the fibrotic scar is collagen type III (Figure S1, Supporting Information). Astrocytes did not interact with vessels within the scar (Figure S2A, Supporting Information). Furthermore, a substantial deposition of Collagen type III was found both within and surrounding endothelial cells, indicating endothelial fibrosis in the chronic phase post‐SCI (Figure S2B, Supporting Information). These suggested a disparity between the regenerated vessels post‐SCI and those in the normal spinal cord.

We further investigated the functionality of the late‐stage (28 dpi) vasculature. Light sheet microscope demonstrated leakage of Evans blue (EB)‐labeled plasma proteins from circulation to the epicenter of the injured spinal cord (Figure 1C), indicating an incomplete BSCB. Furthermore, IF images of CD31 and ZO1 depicted the absence of tight junction proteins among endothelial cells in the epicenter (Figure 1D). IF images also depicted elevated expression of EndoMT markers α‐SMA in endothelial cells within the epicenter, while their expression remained markedly low in the peripheral regions (Figure 1E). This evidence collectively indicated the emergence of pathologically remodeled vessels following SCI, characterized by morphological enlargement, loss of astrocytic coverage, increased deposition of ECM proteins, compromised barrier function, and EndoMT. These aberrant vessels persisted within the fibrotic scar in the chronic phase of SCI.

### 2.2 Osteopontin Promotes Vascular Regeneration following Spinal Cord Injury

To characterize the newly formed vessels post‐injury, we analyzed the public single‐cell dataset GSE162610.^[^ ^26^ ^]^ A distinct subset of Apln^+^ endothelial cells was identified solely post‐injury, marking the presence of endothelial tip cells (Figure S3A, Supporting Information). Apln^+^‐Endo exhibited a unique expression of pro‐angiogenic factors (Figure S3B, Supporting Information). They exhibit the highest levels of stemness within all endothelial celltypes (Figure S3C‐D, Supporting Information), demonstrating a differentiation trajectory similar to that of capillary endothelial cells (Figure S3E, Supporting Information). Analysis of Apln^+^‐Endo enriched genes highlighted biological processes and pathways related to cell migration, regulation of angiogenesis, and tight junctions (Figure S3F‐G, Supporting Information), collectively suggesting that Apln^+^‐Endo functioned in vascular regeneration post‐SCI. IF images further demonstrated minimal Apln expression in uninjured spinal cord tissue but high expression along newly formed vessels post‐injury (Figure S4A‐B, Supporting Information). Western blot (WB) revealed a significant elevation in Apln and Angpt2 expression post‐SCI, peaking at 7 dpi (Figure S4C, Supporting Information).

**Figure 2:**
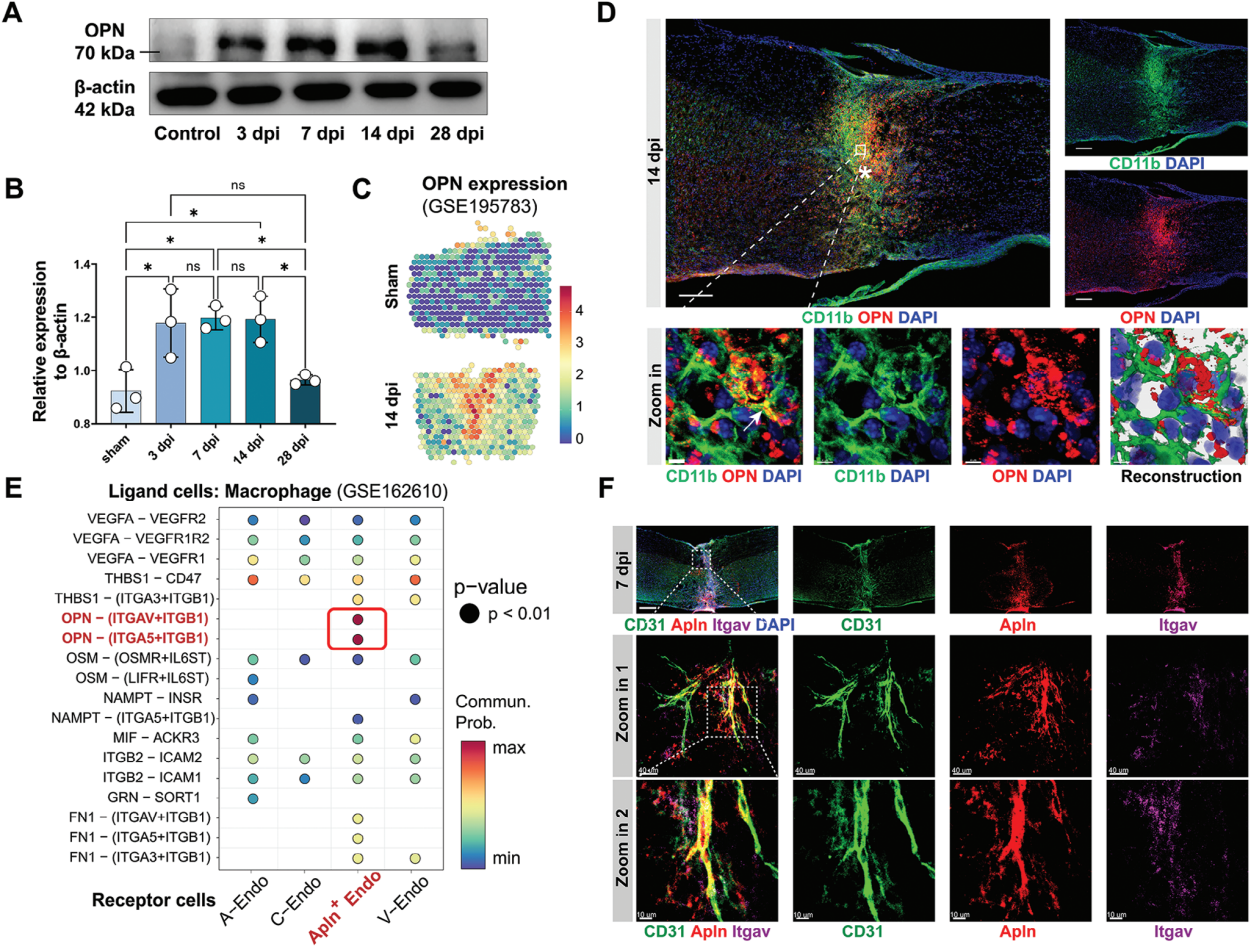
The interaction between OPN and endothelial tip cells. A) Western blots images of OPN and β‐actin protein expression level in the sham or injured spinal cord at 3, 7, 14, and 28 dpi. B) Quantitative analysis of the relative protein expression level of OPN to β‐actin. (n = 3, mean ± SD, one‐way ANOVA, Tukey's multiple comparisons) C) Spatial transcriptomic data illustrating the expression levels and spatial distribution of Opn mRNA in the spinal cord of the sham group and injured spinal cord at 14 dpi (bar: Opn mRNA expression level). D) Representative confocal images (Upper) and local zoom‐in view (Lower) of CD11b (green), OPN (red), and DAPI (blue) in the injured spinal cord at 14 dpi. (Upper scale bar = 200 µm, lower scale bar = 4 µm, * denotes epicenter) E) Dot plot illustrating the communication probability between macrophages and endothelial cells in Ligand‐Receptor interaction analysis from GSE162610. The displayed dots indicate p<0.01. F) Representative confocal images and zoom‐in view of CD31 (green), Apln (red), Itgav (purple), and DAPI (blue) in the injured spinal cord at 7 dpi. (Upper scale bar = 200 µm, middle scale bar = 40 µm, lower scale bar = 10 µm) ns not significant, ∗ p < 0.05.

Meanwhile, the single‐cell dataset GSE162610 revealed a predominant expression of Opn mRNA in macrophages. Opn was minimally expressed in uninjured spinal cords and gradually increased post‐injury (Figure S3H, Supporting Information). WB confirmed the increased expression of OPN post‐injury, reaching its peak at 3, 7, and 14 dpi (**Figure** **2**A‐B). Public spatial transcriptomic data GSE195783 further indicated high expression of Opn in the epicenter (Figure 2C). IF image showed a significant amount of OPN distributed at the injury site, originating from CD11b^+^ myeloid cells and secreted from intracellular to extracellular compartments (Figure 2D). Dataset GSE162610 showed that OPN was highly expressed in the Chemotaxis‐inducing macrophages (Figure S3I, Supporting Information). Importantly, ligand‐receptor interaction analysis revealed that the OPN‐Integrin receptor pair exhibited the strongest communication probability (Figure 2E). OPN signaling predominantly originated from Chemotaxis‐inducing macrophages and acted on Apln^+^ endothelial cells (Figure S3J, Supporting Information). Colocalization of integrin αv (itgav) was detected on Apln^+^‐Endo in the injury site (Figure 2F), collectively indicating a strong interaction between OPN and sprouting angiogenesis post‐SCI.

Subsequently, to determine the proportion of OPN derived from macrophages, we used clodronate liposomes to eliminate macrophages after SCI. Results showed complete clearance of F4/80^+^ macrophages upon treatment, leading to a reduction in OPN expression, albeit remaining higher than the uninjured group (Figure S5A‐C, Supporting Information), suggesting expression of OPN from other cells, such as microglia. Furthermore, using OPN KO mice allowed the deletion of OPN in CD11b^+^ myeloid cells post‐SCI (Figure S5D‐E, Supporting Information). In the sham group, OPN KO mice did not exhibit any functional abnormalities, maintaining spinal cord vascular density and Basso mouse scale (BMS) score as to WT mice. However, post‐SCI, OPN KO mice showed significantly poorer recovery in neurological function (Figure S5F‐G, Supporting Information), aligning with previous research.^[^ ^27^ ^]^

**Figure 3:**
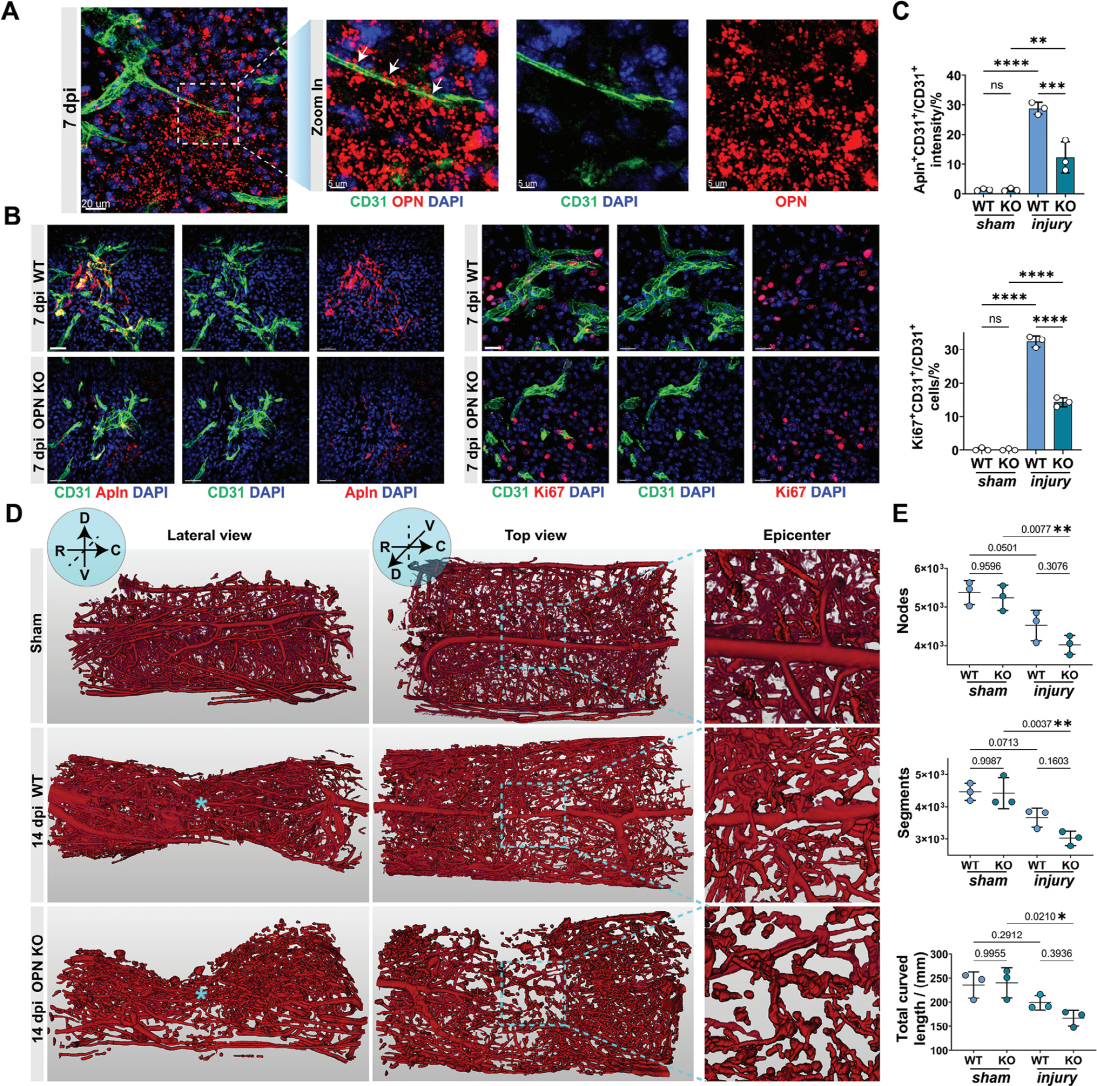
OPN KO mice exhibit impaired vascular regeneration after SCI. A) Representative confocal images and zoom‐in view of CD31 (green), OPN (red), and DAPI (blue) in the epicenter of injured spinal cord at 7 dpi. (Left scale bar = 20 µm, right scale bar = 5 µm) B) Representative confocal images of CD31 (green), Apln/Ki67 (red), and DAPI (blue) in the epicenter of injured spinal cord of WT and OPN KO mice at 7 dpi. (n = 3, Left scale bar = 40 µm, right scale bar = 20 µm) C) Quantitative analysis of Apln^+^CD31^+^/CD31^+^ fluorescence intensity (%) and Ki67^+^CD31^+^/CD31^+^ cells (%) in the sham group and injured spinal cord (7 dpi) of WT and OPN KO mice in (B). (n = 3, mean ± SD, one‐way ANOVA, Tukey's multiple comparisons) D) Representative 3D lateral and top views of Microfil‐perfused spinal cord microvasculature SRµCT data in the sham group and injured spinal cord (14 dpi) of WT and OPN KO mice. (n = 3, D dorsal, V ventral, R rostral, C caudal, * denotes epicenter) E) Quantitative analysis of spinal cord microvasculature in (D), including vasculature nodes, segments, and total curved length. (n = 3, mean ± SD, one‐way ANOVA, Tukey's multiple comparisons, p‐value indicated) ns not significant, ∗ p < 0.05, ∗∗ p < 0.01, ∗∗∗ p < 0.001, ∗∗∗∗ p < 0.0001.

At 7 dpi, the budding branches of the newly formed vascular network extended toward regions where OPN gathered, directly interacting with OPN (**Figure** **3**A). Compared to WT mice, OPN KO mice exhibited a significant decrease in CD31^+^ vascular areas within the injury zone at 7 dpi (Figure S6, Supporting Information). Additionally, there was a notable reduction in the proportion of Apln^+^ vessels and a decreased count of Ki67^+^ endothelial cells within the neo‐vasculature (Figure 3B‐C). 3D reconstruction of microvascular networks from SRµCT data depicted reduced nodes and segments in the intramedullary vasculature at 14 dpi, with a slight decrease in total curved length. However, in OPN KO mice, these microvascular structural parameters showed a more pronounced reduction than in WT mice (Figure 3D‐E). These findings underscored the essential role of OPN in vascular regeneration following SCI.

### 2.3 OPN Induces EndoMT and Fibrosis of SCMECs

**Figure 4:**
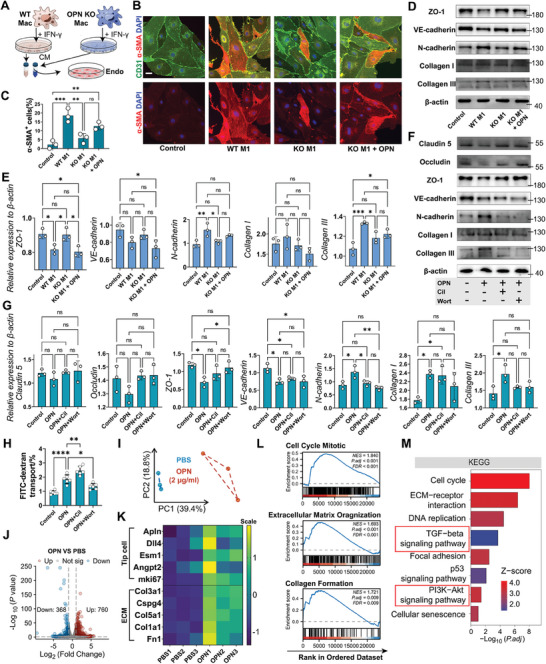
OPN promotes EndoMT and fibrosis of SCMECs in vitro. A) Experimental flowchart for treating SCMECs with Conditioned Medium (CM) from M1‐BMDMs of WT or OPN KO mice. B) Representative IF images of CD31 (green), α‐SMA (red), and DAPI (blue) in SCMECs treated with CM and recombinant OPN protein. (n = 3, Scale bar = 10 µm) C) Quantitative analysis of α‐SMA^+^ SCMECs proportions (%) in (B). (n = 3, mean ± SD, one‐way ANOVA, Tukey's multiple comparisons, ∗∗ p < 0.01, ∗∗∗ p < 0.001) D) Western blot images showing the protein expression levels of ZO‐1, VE‐cadherin, N‐cadherin, Collagen I, Collagen III, and β‐actin in SCMECs with different treatments of CM and recombinant OPN protein. E) Quantitative analysis of the relative protein expression level to β‐actin in (D). (n = 3, mean ± SD, one‐way ANOVA, Tukey's multiple comparisons) F) Western blot images showing the protein expression levels of Claudin5, Occludin, ZO‐1, VE‐cadherin, N‐cadherin, Collagen I, Collagen III, and β‐actin in SCMECs with different treatments of recombinant OPN protein, Cilengitide (Cil), and/or Wortmannin (Wort). G) Quantitative analysis of the relative protein expression level to β‐actin in (F). (n = 3, mean ± SD, one‐way ANOVA, Tukey's multiple comparisons) H) Quantitative analysis of FITC‐dextran transport rate (%) in the trans‐endothelial permeability assay. (n = 6, mean ± SD, one‐way ANOVA, Tukey's multiple comparisons) I) PCA plot showing the sample clusters in bulk RNA‐sequence. J) Volcano plot showing the number of DEGs after OPN treatment. K) Heatmap showing the scaled expression level of genes related to tip cell and ECM in DEGs. L) Gene set enrichment analysis (GSEA) visualization of pathways enriched in DEGs after OPN treatment. M) Histogram showing the ‐log_10_(p.adjust) and Z‐scores of terms enriched by the GO and KEGG analysis of DEGs. ns not significant, ∗ p < 0.05, ∗∗ p < 0.01, ∗∗∗ p < 0.001, ∗∗∗∗ p < 0.0001.

Furthermore, we seek to ascertain the effects of OPN on SCMECs. Recent studies have revealed that substances secreted by M1 pro‐inflammatory macrophages triggered EndoMT.^[^ ^14a^ ^]^ Therefore, we individually treated primary SCMECs with conditioned medium (CM) from M1 bone marrow‐derived macrophages (BMDMs) of WT or OPN KO mice (**Figure** **4**A; Figure S7A, Supporting Information). IF images displayed an increased population of mesenchymal‐like α‐SMA^+^ cells induced by M1 macrophage‐derived CM (Figure 4B‐C). Upon treatment with CM from WT M1 macrophages, a significant decrease in the endothelial cell tight junction protein ZO‐1 and endothelial lineage marker VE‐cadherin was detected, while mesenchymal markers N‐cadherin and collagen expression increased. Conversely, this phenotypic change was reduced in the group treated with CM from OPN KO M1 macrophage. However, reintroducing recombinant OPN protein reinstated EndoMT of SCMECs and collagen secretion (Figure 4D‐E).

We then simultaneously treated SCMECs with recombinant OPN protein as well as the integrin inhibitor cilengitide (Cil) or the PI3K inhibitor wortmannin (Wort). Results showed that OPN induced Akt phosphorylation and increased Apln, Angpt2, and Ki67 expression. However, their elevation was all suppressed with Cil or Wort (Figure S7B, Supporting Information). Cil and Wort both reversed the decrease in claudin‐5, occludin, and ZO‐1 expression, as well as the increase in Collagen III and N‐cadherin induced by OPN, with minimal impact on the expression of VE‐cadherin and Collagen I (Figure 4F‐G). The FITC‐dextran transport experiment showed a significantly elevated cell permeability induced by OPN, which was alleviated by Wort treatment but accentuated with Cil, possibly due to cell adhesion loss upon comprised integrin function (Figure 4H). OPN notably increased wound recovery rates of bEnd.3 cells at 12 h and 24 h, whereas did not enhance endothelial mesh numbers in tube formation assays (Figure S7C‐F, Supporting Information). These findings suggested that OPN's effects on endothelial cells are multifaceted, enhancing their proliferation and migration abilities while compromising their barrier function and tube‐forming capabilities.

Subsequently, after treating SCMECs with 2 µg mL^−1^ recombinant OPN protein, we conducted RNA sequencing. A distinct separation between OPN‐treated endothelial cells and the control group was achieved (Figure 4I). 760 genes were significantly upregulated post‐treatment, while 368 genes were notably downregulated (Figure 4J). Cells treated with OPN displayed a substantial increase in genes related to tip cell formation and ECM organization (Figure 4K). Gene set enrichment analysis (GSEA) revealed pathways significantly altered after OPN treatment, including Cell Cycle Mitotic, Extracellular Matrix Organization, and Collagen Formation (Figure 4L). Pathway enrichment of differentially expressed genes (DEGs) unveiled the involvement of TGF‐β signaling and PI3K‐Akt signaling pathways (Figure 4M). Our findings indicated that OPN, while inducing endothelial cell proliferation, also triggered EndoMT and endothelial fibrosis.

### 2.4 OPN Synergizes with the TGF‐β Signaling Pathway through the Foxo1‐Smad7 Axis

**Figure 5:**
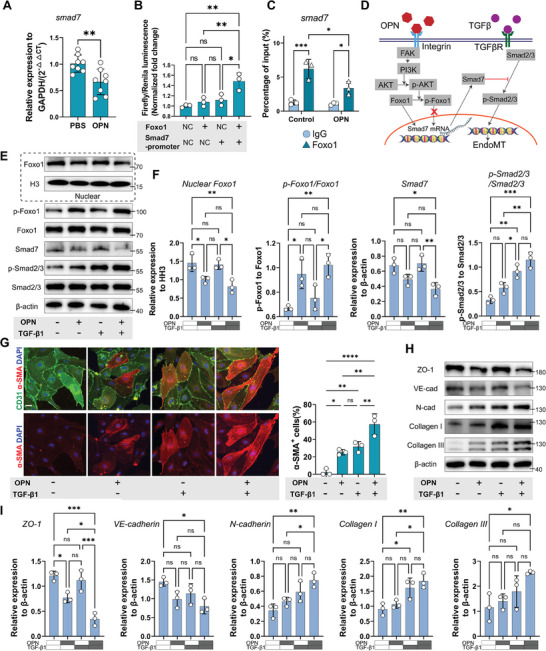
OPN exerts synergistic effects with TGF‐β signaling by reducing Foxo1‐mediated Smad7 transcription. A) Relative expression of Smad7 RNA to GAPDH upon PBS or OPN treatment shown by qRT‐PCR. (n = 8, mean ± SD, unpaired t test) B) The normalized Firefly/Renila luminescence intensity in bEnd.3 cells transfected with Foxo1 overexpression and/or Smad7 promoter plasmid in Dual‐luciferase assay. (n = 3, mean ± SD, one‐way ANOVA, Tukey's multiple comparisons) C) The percentage of input (%) of Smad7 DNA after ChIP pull‐down using IgG or Foxo1 antibody in PBS or OPN‐treated SCMECs. (n = 3, mean ± SD, two‐way ANOVA, Tukey's multiple comparisons). D) Schematic representation of the signal transduction pathway illustrating the synergistic effect of OPN through the Foxo1‐Smad7 axis with the TGF‐β signaling. E) Western blot images showing the protein expression levels of nuclear Foxo1, nuclear Histone H3, p‐Foxo1 (Ser 256), Foxo1, Smad7, p‐Smad2/3, Smad2/3, and β‐actin in SCMECs with different treatment of OPN and/or TGF‐β1. F) Quantitative analysis of the relative protein expression level in (E). (n = 3, mean ± SD, one‐way ANOVA, Tukey's multiple comparisons) G) Representative IF images and quantitative analysis of CD31 (green), α‐SMA (red), and DAPI (blue) in SCMECs with different treatments of OPN and/or TGF‐β1. (Scale bar = 10 µm, n = 3, mean ± SD, one‐way ANOVA, Tukey's multiple comparisons) H) Western blot images showing the protein expression levels of ZO‐1, VE‐cadherin, N‐cadherin, Collagen I, Collagen III, and β‐actin in SCMECs with different treatments of OPN and/or TGF‐β1. I) Quantitative analysis of the relative protein expression level in (H). (n = 3, mean ± SD, one‐way ANOVA, Tukey's multiple comparisons) ns not significant, ∗ p < 0.05, ∗∗ p < 0.01, ∗∗∗ p < 0.001.

After SCI, EndoMT and endothelial fibrosis rely on the downstream activation of the TGF‐β signaling in endothelial cells.^[^ ^14b^ ^]^ In the enriched pathway of DEGs in SCMECs after OPN treatment, the TGF‐β signaling pathway exhibited significance (Figure 4M). We observed a notable downregulation of Smad7, a critical gene within the TGF‐β signaling pathway, following OPN treatment (Figure S8A, Supporting Information), which was further validated through qRT‐PCR (**Figure** **5**A). Further analysis of binding motifs for upstream transcription factors corresponding to the top 200 DEGs revealed Foxo1 as a significant transcription factor (NES = 1.83) (Figure S8B, Supporting Information).

Foxo1 binds to DNA through two major motifs (transfac_pro_M019680 and homer_CTGTTTAC_Foxo1), both of which are enriched in genes including Smad7 (Figure S8C, Supporting Information). From the JASPAR database, both motifs of the mouse Foxo1 transcription factor are present in the promoter sequence of Smad7 (Figure S8D, Supporting Information). To validate Foxo1's activity on Smad7 transcription, we constructed a Foxo1 overexpression plasmid and a smad7 promoter‐firefly_luciferase plasmid. Firefly/Renila luminescence intensity was only enhanced when both plasmids were co‐transfected, indicating Foxo1's binding with the Smad7 promoter (Figure 5B). The ChIP‐qPCR experiment demonstrated the binding of Smad7 promoter DNA on the pulled‐down proteins by Foxo1 antibody, and a significant decrease in Smad7 DNA content was detected after OPN treatment (Figure 5C). Based on these results and known signaling pathways, we summarized the interaction between the OPN and TGF‐β signaling pathways in Figure 5D. Activated Akt could phosphorylate Foxo1, leading to reduced nuclear translocation of Foxo1,^[^ ^28^ ^]^ thereby weakening both the transcription and translation processes of Smad7. With the loss of this crucial negative regulatory factor, the effect of the TGF‐β signaling pathway was further strengthened, causing a phenotype transition in endothelial cells. The siRNA‐mediated knockdown of Foxo1 also reduced Smad7 expression and increased Smad2/3 phosphorylation level (Figure S8E, Supporting Information).

In primary SCMECs treated individually with OPN, TGF‐β1, and both, the nuclear shuttle of Foxo1 was observed only upon the addition of OPN. This was manifested by decreased nuclear Foxo1 and increased phosphorylated Foxo1. Upon OPN treatment, Smad7 expression significantly decreased, resulting in the highest level of phosphorylated Smad2/3 in the co‐treatment group, indicating excessive activation of the TGF‐β signaling (Figure 5E‐F). IF images revealed an increased proportion of α‐SMA^+^ cells in the co‐treatment group (Figure 5G). The expression of ZO‐1 and VE‐cadherin decreased in the OPN‐treated group and was further reduced in the co‐treatment group. In contrast, N‐cadherin, Collagen I, and Collagen III expression increased following OPN or TGF‐β1 treatment, with the most significant increase in the co‐treatment group (Figure 5H‐I). These findings strongly proved the synergistic effect between OPN and TGF‐β1, highlighting the bridging role of the Foxo1 nuclear shuttle and decreased Smad7 expression.

### 2.5 Targeted Delivery of Small Molecule Alleviates OPN‐induced Pathological Vascular Remodeling by Reducing Foxo1 Phosphorylation

**Figure 6:**
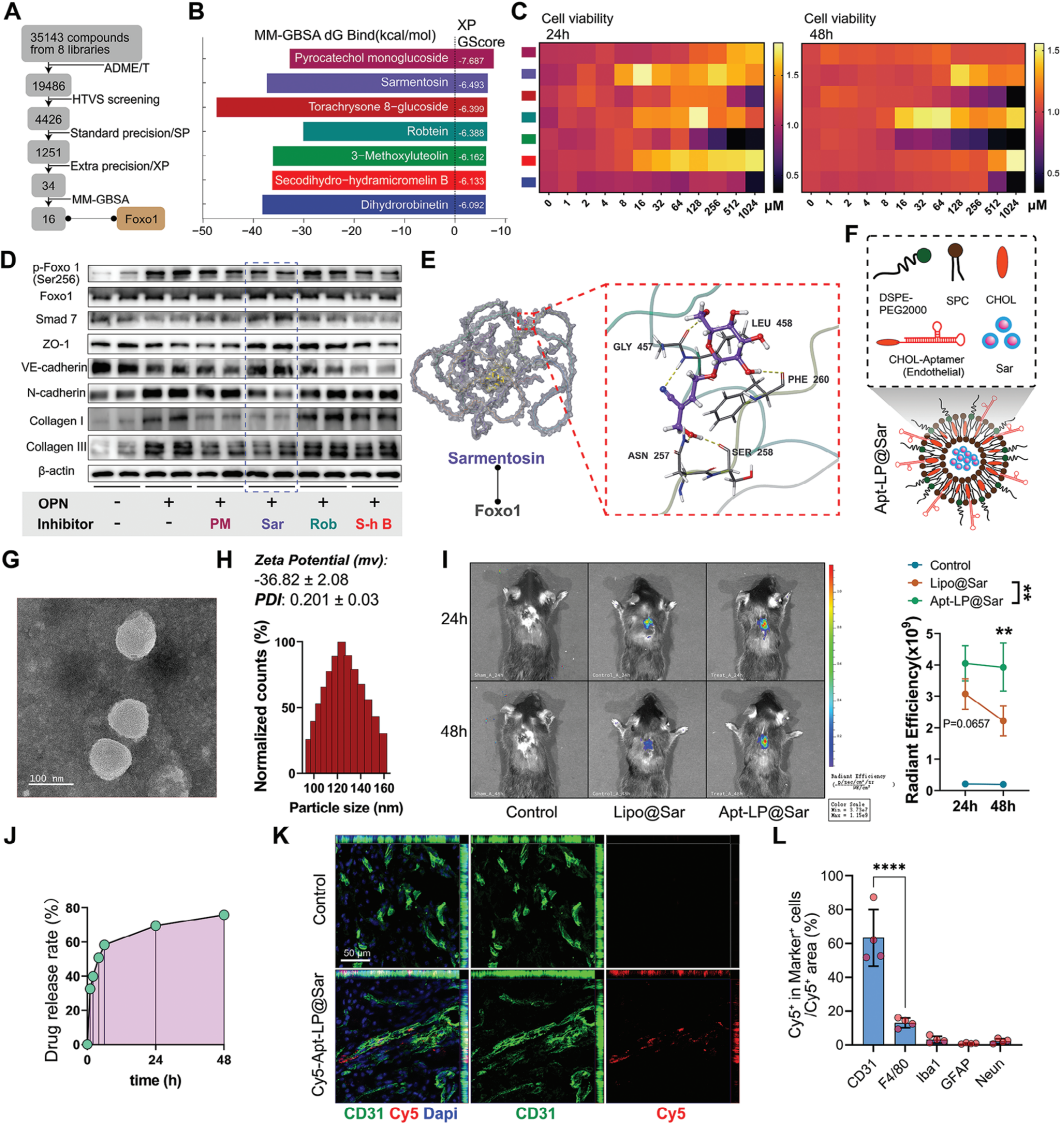
Developing aptamer‐liposomes encapsulated small molecule targeting phosphorylation of Foxo1 in endothelial cells. A) Flowchart illustrating the molecular docking process for screening small‐molecule drugs targeting Foxo1. B) Histogram showing the top seven small‐molecule drugs ranked by MM‐GBSA dG Bind (kcal mol^−1^) and XP GScore. C) Heatmap showing the cell viability of SCMECs determined by CCK8 assay after treatment with different drugs for 24 h and 48 h. D) Western blot images showing the protein expression levels of p‐Foxo1 (Ser 256), Foxo1, Smad7, ZO‐1, VE‐cadherin, N‐cadherin, Collagen I, Collagen III, and β‐actin with different treatment of small‐molecule drugs. (n = 4) Quantitative analysis of the relative protein expression level is presented in Figure S8F (Supporting Information). E) Molecular schematic diagram of the interaction between Sarmentosin (Sar) and Foxo1 protein. Sar deeply penetrates into the binding pocket of Foxo1, establishing hydrophobic interactions with residues such as ALA455 and PRO456, and forming hydrogen bonds with residues GLY457, LEU458, PHE260, SER258, and ASN257 (wherein the murine Foxo1 protein's SER258 site corresponds to the human Foxo1 protein's phosphorylated SER256 site). F) Illustration of the assembly of Apt‐LP@Sar using DSPE‐PEG2000, soybean phosphatidylcholine (SPC), cholesterol molecules (CHOL), Aptamer‐CHOL, and Sar. G) Representative TEM images illustrating the morphology and dispersity of Aptamer‐liposome encapsulated sarmentosin (Apt‐LP@Sar). (Scale bar = 100 nm) H) Representative results of nanoparticle size and zeta potential analysis of Apt‐LP@Sar from the phase analysis light scattering (PALS) mode. I) Representative in vivo images and quantitative analysis of total radiant efficiency in the control and treatment groups at 24 h and 48 h after intravenous injection of saline, Dir‐labeled Lipo@Sar, and Dir‐labeled Apt‐LP@Sar. (color bar gradient: total radiant efficiency, n = 3, mean ± SD, two‐way ANOVA, Tukey's multiple comparisons) J) Drug release rate (%) of Apt‐LP@Sar within 48 hours determined by UV‐spectroscopy. K) Representative confocal images of in vivo tracing showing the co‐localization of Apt (Cy5, red)‐LP@Sar with endothelial cells (CD31, green) in the epicenter. (Scale bar = 50 µm, n = 4) L) Quantitative analysis of the proportion of the total Cy5 ^+^ Apt‐LP@Sar area in different cells (Figure S9C, Supporting Information). (n = 4, mean ± SD, one‐way ANOVA, Tukey's multiple comparisons) ∗∗ p < 0.01, ∗∗∗∗ p < 0.0001.

Given the impediment to vascular regeneration through direct inhibition of OPN, we endeavored to develop a Foxo1 phosphorylation inhibitor that selectively interfered with the Foxo1 nuclear shuttle, mitigating EndoMT and fibrosis without compromising endothelial cell proliferation. From a screening of 35 143 small molecule drugs across eight drug compound libraries, ADME/T assessment identified 19 486 compounds with clinical drug potential. Molecular docking via HTVS yielded 4426 molecules exhibiting strong interactions with Foxo1. Further refinement through standard precision (SP), extra precision (XP) screening, and MM‐GBSA analysis identified 16 compounds meeting the selection criteria (**Figure** **6**A, Expanded Methods). Finally, by ranking the XP score and MM‐GBSA binding free energy, seven compounds with stable binding and high druggability were chosen for subsequent experimental screening (Figure 6B). CCK8 assays revealed notable cytotoxicity at high concentrations of Torachrysone 8‐glucoside, 3‐Methoxyluteolin, and Dihydrorobinetin, while the remaining four compounds exhibited no discernible cytotoxic effects (Figure 6C). Results demonstrated that sarmentosin (Sar) most effectively reduced Foxo1 phosphorylation level and restored Smad7 expression, alleviating TGF‐β signaling excessive activation, EndoMT, and fibrosis (Figure 6D; Figure S8F‐H, Supporting Information). Sar also showed an inhibitory effect on the activation of mTOR signaling induced by OPN but had no significant effect on the phosphorylation of GSK‐3β (Figure S8H, Supporting Information). Molecular docking analysis revealed Sar's deep penetration into the binding pocket of Foxo1 and establishing hydrogen bonds with residues SER258 (Figure 6E), which supported Sar's inhibition of the phosphorylation of Foxo1.

To facilitate targeted delivery to the endothelium, we employed liposome encapsulation of Sar, followed by conjugation with an endothelium‐specific aptamer sequence, resulting in the assembly of aptamer‐liposome encapsulated sarmentosin (Apt‐LP@Sar) for intravenous administration (Figure 6F). The previously screened aptamer (CA2), a 33‐base single‐stranded DNA sequence, exhibited strong specific binding affinity to endothelial cells.^[^ ^29^ ^]^ Transmission electron microscopy (TEM) revealed the uniform spherical structure of Apt‐LP@Sar, with particle size measuring 127.10 ± 3.89 nm in diameter (Figure 6G‐H). The zeta potential is −36.82 ± 2.08 mV, and the polymer dispersity index (PDI) is 0.201 ± 0.03. Multiple batches of Apt‐LP@Sar demonstrated good reproducibility (Figure S9A, Supporting Information). The encapsulation efficiency of the drug in liposomes was determined to be 59.2%. In vivo imaging of mice demonstrated that the CA2‐bound liposomes persisted longer in the injury site, exhibiting higher total radiant efficiency in the injury site at 48 h post‐injection (Figure 6I). UV spectrum‐based drug release analysis also indicated that 75.8% of the encapsulated drug was released after 48 hours (Figure 6J). Dio‐labeled Apt (Cy5)‐LP@Sar treated SCMECs exhibited co‐staining of Dio‐labeled cell membranes with Cy5 fluorescence, suggesting successful binding of CA2 to the surface of the liposomes (Figure S9B, Supporting Information). In vivo tracing experiments using Apt (Cy5)‐LP@Sar revealed the highest proportion of uptake by CD31^+^ endothelial cells post‐SCI, validating its targeting ability (Figure 6K‐L; Figure S9C, Supporting Information).

**Figure 7:**
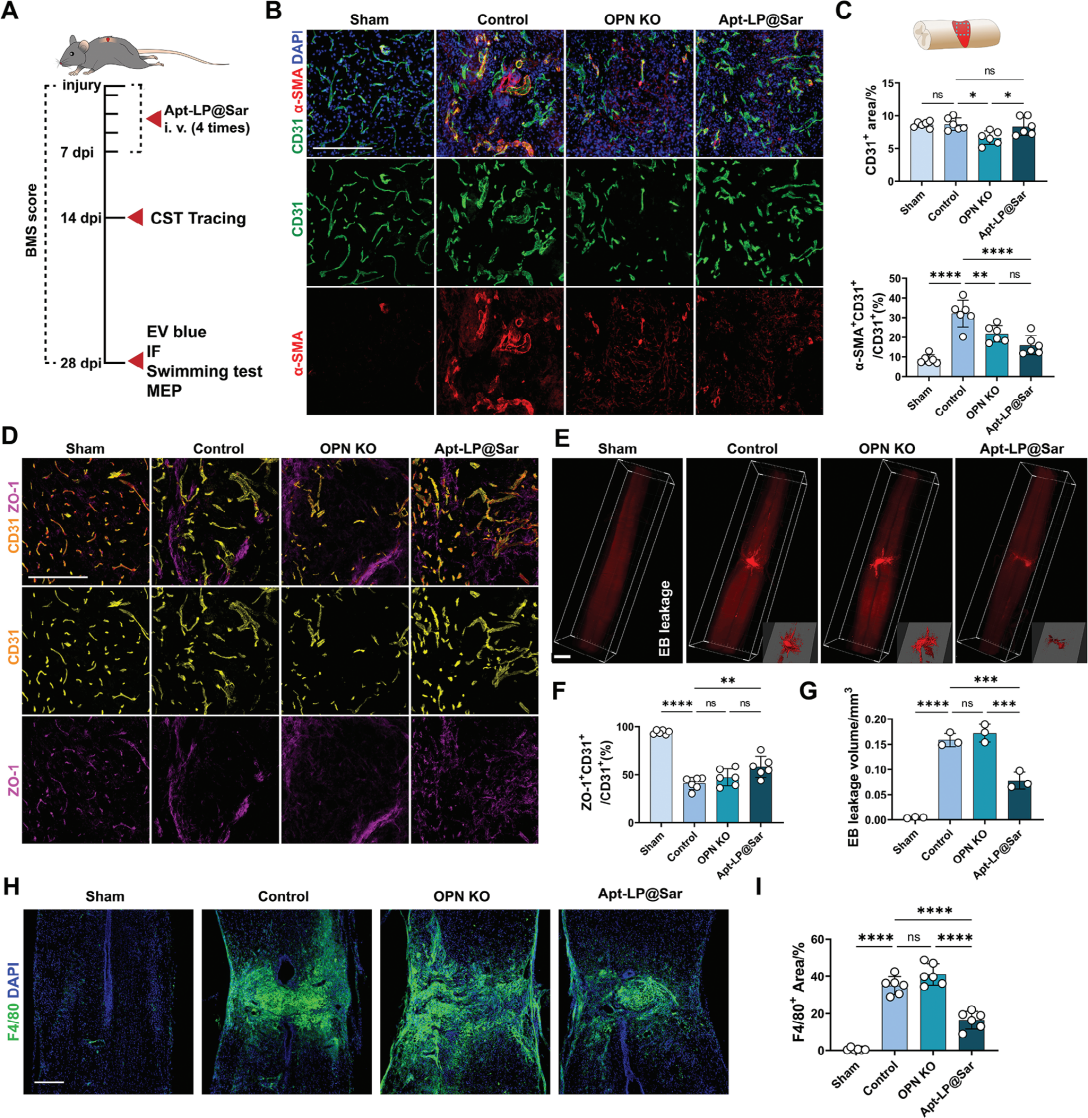
Apt‐LP@Sar alleviates pathological vascular remodeling and BSCB disruption after SCI. A) Illustration of the process of intravenous injection of the Apt‐LP@Sar and evaluation in mice after SCI. B) Representative IF images of CD31 (green), α‐SMA (red), and DAPI (blue) in the epicenter of the sham, control, OPN KO, and Apt‐LP@Sar‐treated spinal cord at 28 dpi. (Scale bar = 200 µm, n = 6) C) Quantitative analysis of epicenter CD31^+^ area (%) and α‐SMA^+^CD31^+^/ CD31^+^ area (%) in (B) (n = 6, mean ± SD, one‐way ANOVA, Tukey's multiple comparisons) D) Representative IF images of CD31 (yellow) and ZO‐1 (purple) in the epicenter of the sham, control, OPN KO, and Apt‐LP@Sar‐treated spinal cord at 28 dpi. (Scale bar = 200 µm, n = 6) E) Representative light sheet‐microscope 3D images of the spinal cord after EB leakage (red) experiment in the spinal cord of the sham, control, OPN KO, and Apt‐LP@Sar‐treated groups at 28 dpi. (Scale bar = 500 µm, n = 3) F) Quantitative analysis of ZO‐1^+^CD31^+^/ CD31^+^ area (%) in (D) (n = 6, mean ± SD, one‐way ANOVA, Tukey's multiple comparisons) G) Quantitative analysis of EB leakage volume in the spinal cord (%) in (E) (n = 3, mean ± SD, one‐way ANOVA, Tukey's multiple comparisons) H) Representative IF images of F4/80 (green) and DAPI (blue) in the spinal cord of the sham, control, OPN KO, and Apt‐LP@Sar‐treated groups at 28 dpi. (Scale bar = 200 µm, n = 6) I) Quantitative analysis of F4/80 ^+^ area (%) in the spinal cord. (n = 6, mean ± SD, one‐way ANOVA, Tukey's multiple comparisons) ns not significant, ∗ p < 0.05, ∗∗ p < 0.01, ∗∗∗ p < 0.001, ∗∗∗∗ p < 0.0001.

Next, following injections of Apt‐LP@Sar at days 1, 3, 5, and 7 post‐SCI, we evaluated the effects of Apt‐LP@Sar on SCI mice (**Figure** **7**A). IF images at 28 dpi displayed decreased areas of α‐SMA^+^ endothelial cells in OPN KO mice. However, the vascular density notably decreased, indicating impaired vascular regeneration in the OPN KO group. In contrast, in the Apt‐LP@Sar treatment group, vascular density remained unchanged, and areas of α‐SMA^+^ endothelial cells notably decreased (Figure 7B‐C). Also, the ZO‐1 expression was partially restored in the epicenter of injured spinal cord (Figure 7D,F). EB experiments revealed significantly decreased plasma protein leakage in the injury zone of the Apt‐LP@Sar treated group at 28 dpi (Figure 7E,G), concurrently reducing infiltration and residue of macrophages (Figure 7H‐I). These findings demonstrated that Apt‐LP@Sar significantly alleviates OPN‐induced traumatic pathological vascular remodeling by specifically targeting endothelial cells in the epicenter.

### 2.6 The Efficacy of Aptamer‐liposome Encapsulated Sarmentosin in Spinal Cord Injury Treatment

**Figure 8:**
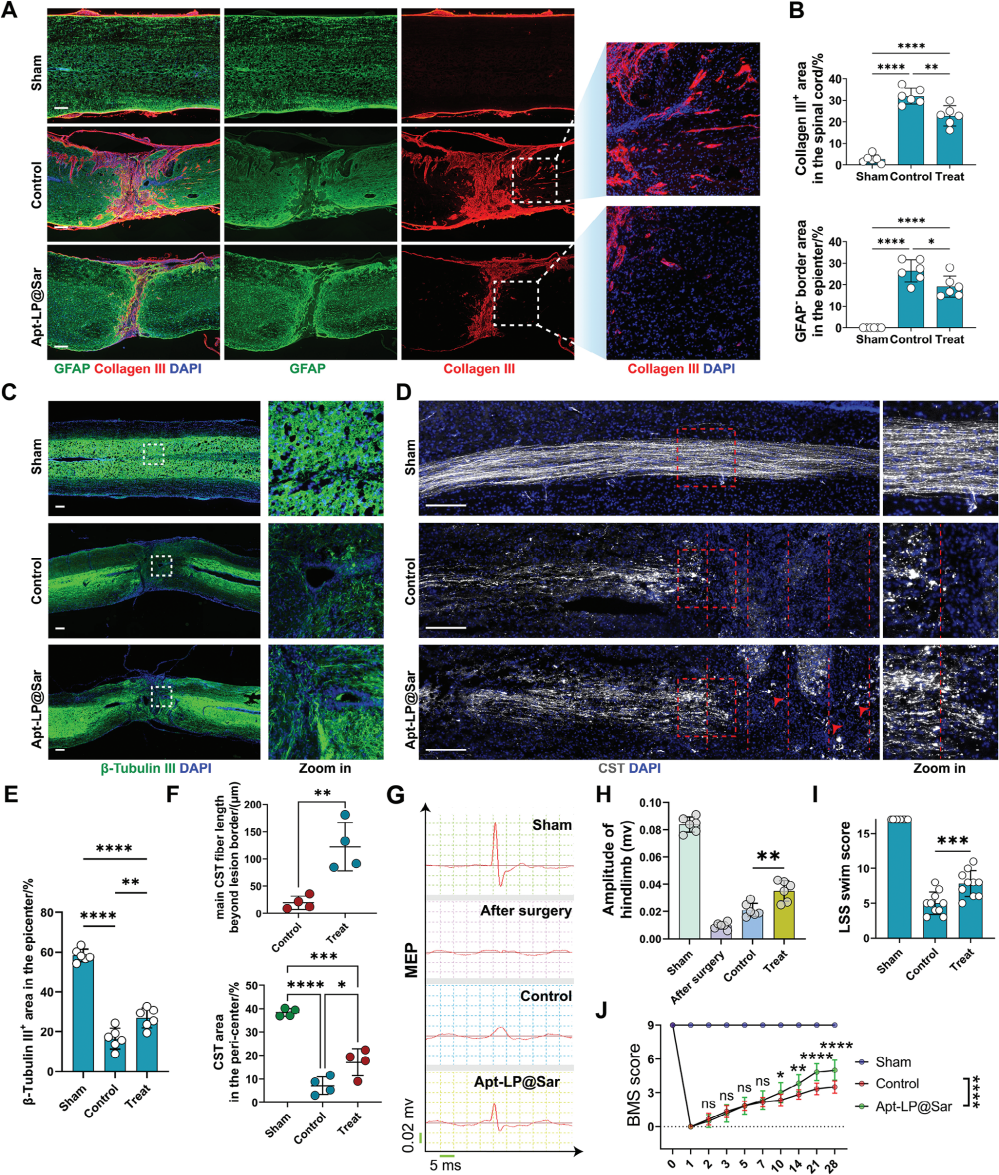
Apt‐LP@Sar promote axon regeneration and functional recovery in mice following SCI. A) Representative IF images of GFAP (green), Collagen III (red), and DAPI (blue) in the spinal cord of the sham, control, and Apt‐LP@Sar‐treated groups at 28 dpi. (Scale bar = 200 µm, n = 6). B) Quantitative analysis of Collagen III ^+^ area and GFAP^−^ border area in the spinal cord (%) in (A). (n = 6, mean ± SD, one‐way ANOVA, Tukey's multiple comparisons) C) Representative IF images and local zoom‐in views of β‐Tubulin III (green) and DAPI (blue) in the spinal cord of the sham, control, and Apt‐LP@Sar‐treated groups at 28 dpi. (Scale bar = 200 µm, n = 6). D) Representative fluorescence images and local zoom‐in views of dextran‐labeled CST (white) and DAPI (blue) in the spinal cord of the sham, control, and Apt‐LP@Sar‐treated groups at 28 dpi. (Scale bar = 200 µm, Intervals of red dashed lines = 200 µm, n = 4) E) Quantitative analysis of β‐Tubulin III ^+^ area in the epicenter (%) in (C). (n = 6, mean ± SD, one‐way ANOVA, Tukey's multiple comparisons) F) Quantitative analysis of main CST fiber beyond lesion border and CST area in the peri‐center in (D). (n = 4, mean ± SD, unpaired t‐test, and one‐way ANOVA, Tukey's multiple comparisons) G) Representative images of hindlimb motor evoked potentials (MEPs) in the sham, after surgery, control, and Apt‐LP@Sar‐treated groups at 28 dpi. H) Quantification of the amplitude of hindlimb in (G) (n = 6, mean ± SD, one‐way ANOVA, Tukey's multiple comparisons). I) Quantification of the swimming test using the Louisville swim scale (LSS) swim score (n = 10, mean ± SD, one‐way ANOVA, Tukey's multiple comparisons). J) Basso mouse scale (BMS) scores over time post‐injury in the sham, control, and Apt‐LP@Sar‐treated groups. (n = 10, mean ± SD, two‐way ANOVA, Tukey's multiple comparisons) ns not significant, ∗ p < 0.05, ∗∗ p < 0.01, ∗∗∗ p < 0.001, ∗∗∗∗ p < 0.0001.

Finally, we assessed tissue repair and mouse neurological functions after the administration of Apt‐LP@Sar. GFAP and Collagen III co‐staining showed a significant reduction in Collagen III^+^ scar and GFAP^−^ border area in the treatment group. Additionally, vessel‐like Collagen III depositions (indicated by white arrows) substantially decreased. These findings together indicated reduced fibrosis, regression of fibrotic scars, and corralling of glial scars in the treated group (**Figure** **8**A‐B). Immunostaining of β‐tubulin III revealed a more sparse and limited distribution of neural fibers within the control group compared to the sham group. However, the treatment group displayed significantly increased β‐tubulin III^+^ nerve fibers at the epicenter than the control group (Figure 8C,E). Anterograde tracing from the cerebral cortex depicted CST terminating at the injury site, while Apt‐LP@Sar treatment extended longer CST fibers beyond the lesion border and more CST fiber area in the peripheral regions of the injury site than the control group (Figure 8D,F).

MEPs assessment demonstrated significantly increased hindlimb amplitude in the treatment group compared to controls (Figure 8G‐H). In the swimming test, mice in the treatment group exhibited considerable improvement in hind limb motor ability, forelimb dependency, trunk stability, and body angles, with a markedly higher Louisville swim scale (LSS) swimming score than the control group (Figure 8I). Continuous 28‐day BMS scoring post‐SCI revealed significantly enhanced hind limb motor function in the treatment group starting at 10 dpi, showing better motor functional recovery compared to the control group. By 28 dpi, mice exhibited frequent or consistent plantar stepping and occasional coordination, scoring 5 – 6 points (Figure 8J). Overall, intravenous administration of Apt‐LP@Sar reduced fibrotic scar area and significantly promoted neural regeneration and functional recovery in mice post‐SCI, showing promising clinical application potential.

## 3 Discussion

In this study, significant progress was made in understanding the pathophysiological mechanisms of SCI by elucidating the role of OPN in vascular regeneration and pathological remodeling. OPN activated the Itgav/PI3K/Akt signaling pathway, initiating the early stages of angiogenesis. Furthermore, OPN‐induced Akt phosphorylation promoted the phosphorylation and extranuclear transport of Foxo1. This mechanism decreases Smad7 expression and excessively activates TGF‐β signaling, which subsequently triggers EndoMT, representing a critical intervention point. Vascular remodeling frequently originates from EndoMT.^[^ ^30^ ^]^ This remodeling is pivotal for understanding post‐SCI fibrosis and for developing therapeutic interventions. The development of Apt‐LP@Sar, which targets Foxo1 phosphorylation, presents an innovative approach to mitigate pathological vascular remodeling and fosters a conducive microenvironment for neural restoration. This endothelium‐specific targeting strategy provides a promising avenue for SCI treatment by counteracting the effects of OPN on TGF‐β excessive activation in SCMECs. Overall, our research not only deepened the understanding of SCI repair mechanisms but also introduced a targeted therapeutic strategy, potentially impacting treatments for conditions characterized by pathological vascular remodeling and fibrosis.

The role of OPN in vascular regulation following neural trauma was revealed in this work. In recent studies, the role of OPN in the nervous system has gradually been elucidated. As a modulator of immune responses occurring in the central nervous system post‐injury, OPN exhibits dual effects. Elevated OPN during the acute phase of a disease is necessary for the timely migration and function of immune cells, while persistently high levels of OPN during the chronic phase may lead to excessive inflammatory responses.^[^ ^16^, ^17^ ^]^ OPN is essential for the plasticity, phagocytic activity, and pro‐inflammatory response of microglia.^[^ ^31^ ^]^ This study showed that OPN is one independent factor in the regulation of vascular regeneration. After the primary injury to the spinal cord, hypoxia emerges as a primary factor affecting endothelial cells, initiating sprouting angiogenesis mediated by tip cells.^[^ ^32^ ^]^ Tip cells, along with the Apln signal, serve as initiators of angiogenesis.^[^ ^33^ ^]^ Then, both the hypoxic environment and the metabolic byproducts of anaerobic glycolysis induce vascular remodeling and EndoMT.^[^ ^30^, ^34^ ^]^ Zhou et al. demonstrated that microvascular endothelial cells engulf myelin debris after SCI, resulting in fibrosis.^[^ ^14b^ ^]^ Our study demonstrated that OPN could independently induce EndoMT and increase ECM secretion, emphasizing the significance of the immune cell‐endothelial cell crosstalk following SCI, as we previously proposed.^[^ ^35^ ^]^ In the RNA sequencing of SCMECs, OPN treatment upregulated the expression of a large number of ECM‐related genes (Figure 4K), and the proteins encoded by these ECM genes were all associated with fibrotic scar formation post‐SCI.^[^ ^13^, ^18^, ^36^ ^]^ Therefore, we posited that the factors triggering endothelial cell phenotypic transitions after SCI are diverse, and OPN was a previously unexplored factor. OPN KO may also affect the regeneration of epidural large vessels. Research suggests that OPN can promote the proliferation of vascular smooth muscle cells.^[^ ^37^ ^]^ Following SCI, cells in the dura are also affected by the altered microenvironment ^[^ ^38^ ^]^ and OPN KO may affect the remodeling of large epidural vessels. However, our study primarily focused on changes in the intramedullary microvasculature and did not experimentally verify alterations in large vessel regeneration.

Traumatic pathological vascular remodeling following SCI presents a major obstacle to scar repair and requires targeted intervention. Pathological vascular remodeling has predominantly been studied in cardiovascular diseases, tumors, and retinal disorders.^[^ ^39^ ^]^ This study, for the first time, characterized traumatic pathological vascular remodeling following SCI. Evidence indicates that accelerating angiogenesis in the acute phase of injury is advantageous, as it reduces tissue ischemia and hypoxia.^[^ ^40^ ^]^ Nonetheless, successful vascular regeneration involves not only restoring vascular density but also ensuring the normal function of blood vessels. In a functional neurovascular unit, endothelial cells, basement membrane, pericytes, astrocytic endfeet, and neuronal innervation, collaborate to support substance transport.^[^ ^41^ ^]^ Within the chronic phase, the disruption of the BSCB persists, further prompting our attention to vascular remodeling.^[^ ^10^ ^]^ Williamson et al. summarized vascular remodeling following stroke as decreased blood flow, reduction of the basement membrane, decreased pericyte coverage, and increased permeability.^[^ ^42^ ^]^ For vasculature in the epicenter, the lack of astrocytic coverage or neuronal innervation around the blood vessels results in compromised barrier function and potentially undiscovered abnormalities in blood flow regulation.^[^ ^43^ ^]^ In the fibrotic scars of the injured spinal cord, we characterized pathological vascular remodeling by the following features: loss of astrocytic coverage, increased vessel diameter, enhanced permeability, and extensive ECM deposition. The pathologically regenerated vasculature with incomplete BSCB structures leads to continuous leakage of macrophages and inflammatory substances, making the scar area “an inhospitable terrain” for neural tissue. The limited regenerative capacity of disrupted neuronal cells, combined with the unsuitable environment for axon regeneration in the dense fibrotic scar region, poses challenges for tissue repair.

Fibrotic scar formation represents a critical pathophysiological change driven by pathological vascular remodeling. After SCI, reactive astrocytes, oligodendrocyte progenitor cells, and activated microglia form protective barriers that limit damage spread and contribute to glial scar formation.^[^ ^44^ ^]^ Concurrently, various cell types, primarily fibroblasts, deposit proteins that affect axon growth, such as CSPGs, laminin, collagen, and fibronectin, which contribute to the development of a dense fibrotic scar at the injury epicenter. This scar acts as a significant barrier to endogenous repair mechanisms.^[^ ^45^ ^]^ The role of glial scars in axon regeneration is controversial.^[^ ^46^ ^]^ Glial scars may not be the fundamental reason why axons struggle to traverse the scar and regenerate. Genetically removing reactive astrocytes did not substantially promote axon regeneration at the injury site.^[^ ^47^ ^]^ Researchers suggest that glial scars may be beneficial to axon regeneration in the early stages, as they can limit damage spread and even secrete neurotrophic factors. However, in later stages, chronic glial scars primarily act as barriers and inhibitors to axon regeneration due to the increased production of fibrotic ECM components.^[^ ^48^ ^]^ However, reducing the deposition of fibrous ECM in both the boundary zone and injury epicenter of fibrotic scars can be beneficial for SCI repair.^[^ ^13^ ^]^ Therefore, we believe that intervention in fibrotic scars, especially targeting ECM components that hinder neural regeneration, is crucial. During the pathological remodeling of microvasculature and EndoMT, many ECM proteins, such as type III Collagen (Figure 8A), are produced, which has greatly hindered neural regeneration. Mouse SCI models also provide a platform for observing and quantifying fibrotic scar formation after SCI.^[^ ^18^ ^]^ Consequently, this work concentrated on the pathological remodeling of microvascular cells in the epicenter, whose phenotypic changes significantly contribute to fibrotic scar formation.

The phenotypic transition of SCMECs plays a critical role in traumatic pathological vascular remodeling. OPN may interact synergistically with factors such as hypoxia, cellular debris, exosomes, and others during this process. However, the phenomenon referred to as EndoMT or fibrosis in this study does not involve a complete transformation from endothelial cells to mesenchymal cells in vivo but rather an intermediate EndoMT phenotype. EndoMT frequently occurs in wound healing and is initiated by factors such as oxidative stress, hypoxia, abnormal shear stress, and inflammation. The main molecular drivers are TGF‐β, Notch, and ox‐LDL.^[^ ^49^ ^]^ During the process of EndoMT, endothelial cells acquire an intermediate EndMT phenotype, characterized by increased migratory ability and reduced cell‐cell contacts. We observed that the endothelial cells after SCI express both CD31 and α‐SMA, indicating an intermediate EndoMT phenotype (Figure 1D). At this stage, cells express markers of both endothelial cells and mesenchymal cells, and they also express fibrotic‐associated proteins such as collagen and fibronectin.^[^ ^21^ ^]^ Cells in this intermediate state still have the potential for reprogramming to an endothelial cell phenotype, thus representing promising targets for intervention. The complete conversion of endothelial cells into mesenchymal cells or fibroblasts after SCI requires cautious investigation, supported by cell lineage tracing experiments in transgenic mice.^[^ ^50^ ^]^

The TGF‐β signaling pathway is crucial for the induction of EndoMT and fibrosis.^[^ ^21^ ^]^ The activation of Smad2/Smad3 by TGF‐β is a downstream key signaling in EndoMT and tissue fibrosis, while Smad7 serves as a negative feedback regulator of the Smad‐dependent TGF‐β signaling pathway.^[^ ^51^ ^]^ Inhibition of the TGF‐β signal efficiently prevents myelin debris‐induced EndoMT.^[^ ^14b^ ^]^ During tissue fibrosis, the activation of latent TGF‐β involves the collaboration of proteases, integrins, and specific ECM molecules.^[^ ^52^ ^]^ In this study, we identified for the first time the synergistic action of OPN with TGF‐β signaling, whereby OPN directly decreased the expression of the antifibrotic factor Smad7. OPN initially activates the integrin‐mediated PI3K‐Akt signaling pathway.^[^ ^53^ ^]^ Phosphorylated Akt acts on various transcription factors, thereby influencing the expression of downstream effector proteins, which is a primary mechanism of action for OPN in cells. Akt phosphorylation decreases the activity of p21 and p27, thus promoting cell cycle progression.^[^ ^54^ ^]^ We also demonstrated that OPN activates both the mTOR and GSK3β signaling, which is the downstream of PI3K‐Akt signaling pathway. Activation of mTOR may also induce EndoMT, while GSK3β phosphorylation primarily promotes endothelial cell proliferation and migration.^[^ ^55^ ^]^ Sar slightly reduced mTOR phosphorylation but had no significant effect on GSK3β phosphorylation (Figure S8H, Supporting Information). This suggests that its inhibition of Foxo1 phosphorylation protects the endothelial cell phenotype without causing significant cytotoxicity or inhibiting proliferation.

The Foxo family constitutes the main regulator of endothelial cell homeostasis.^[^ ^56^ ^]^ Foxo1 is a crucial transcription factor that regulates the homeostasis and plasticity of endothelial cells. Both systemic knockout and endothelial‐specific knockout of Foxo1 result in embryonic lethality and impaired vascular development.^[^ ^57^ ^]^ Further research reveals that endothelial‐specific deletion of Foxo1 in mice induces endothelial cell proliferation but leads to abnormal vascular morphology, including vessel enlargement and hyperplasia, which disrupts coordinated angiogenesis. Conversely, elevated expression of Foxo1 restricts vascular expansion, resulting in vessel thinning and hypobranching.^[^ ^58^ ^]^ Andrade et al. further have shown that the activation of Foxo1 promotes a quiescent endothelial state through the production of S‐2‐hydroxyglutarate.^[^ ^56^ ^]^ Therefore, the balance of Foxo1 activity regulates the biological properties of the endothelial cell. Enhancing the transcriptional activity of Foxo1 is advantageous in alleviating vascular remodeling. The delivery of a Foxo1 stimulus, paclitaxel, has been used to treat pulmonary hypertension.^[^ ^59^ ^]^ We reported changes in the phosphorylation level and nuclear content of Foxo1 after OPN treatment, demonstrating that OPN could influence the nuclear shuttle of Foxo1 and subsequently affect the expression of the Smad7 protein through its canonical PI3K‐Akt pathway.^[^ ^60^ ^]^ Considering the essential role of Smad7 in the inhibitory of the TGF‐β pathway and promotion of tissue fibrosis.^[^ ^61^ ^]^ The discovery in our study established a novel bridging between Foxo1‐mediated endothelial homeostasis and the TGF‐β signaling pathway activation, ultimately shedding light on fibrosis after SCI. However, this study still has limitations regarding the roles of TGF‐β and Foxo1 in vivo. Further research into their expression and activation will aid in elucidating the status of endothelial cells in the injured microenvironment and provide a more solid theoretical basis for drug applications.

The development of cell‐targeted small molecule therapies for SCI offers several advantages, including minimizing off‐target effects, enhancing efficacy, potential for combination therapies, and prolonged therapeutic effects. It is undeniable that both OPN and TGF‐β play roles in neuronal survival after SCI, and direct intervention may lead to adverse outcomes.^[^ ^27^, ^62^ ^]^ Therefore, we exploited the regulatory capacity of Foxo1 in balancing endothelial cell proliferation and differentiation and screened the small‐molecule drug Sar to inhibit Foxo1 phosphorylation. Through this approach, we preserved the ability of OPN to support vascular regeneration and its other potential functions during tissue repair. Furthermore, by utilizing liposomes coupled with endothelial cell‐specific aptamers for encapsulation, we improved drug stability and minimized side effects on other cell types. The Apt‐LP@Sar exhibited excellent dispersion, reproducibility, biocompatibility, endothelial targeting, and injury site targeting. Overall, the aptamer‐liposome carrier represented an effective and easily producible strategy for in vivo targeted drug delivery to specific cells and SCI treatment.

## 4 Conclusion

In summary, inhibition of endothelium‐specific Foxo1 phosphorylation significantly reduced traumatic pathological vascular remodeling, decreased fibrotic scar, enhanced axon regeneration, and improved functional recovery in mice following SCI. By elucidating the molecular mechanisms through which OPN affects endothelial cells, a reliable vascular intervention‐targeted therapy was developed, showing promising preclinical potential.

## 5 Experimental Section

### Data Availability and Ethics

The data, analytical methods, and study materials are accessible to researchers upon request to reproduce the results or replicate the procedures. All necessary materials are housed in the laboratory at Central South University (CSU). Comprehensive details on methods and materials are provided in the Expanded Methods and Major Resources Table. All animal protocols and experimental procedures were conducted following the guidelines of the Ethics Committee of CSU and adhered to the guide for the care and use of laboratory animals (No. 2 022 020 623).

### Animals

8‐week‐old female mice, with a weight range of 20–25 g were utilized in all experiments. C57BL/6 mice were procured from Hunan SJA Laboratory Animal Company. OPN KO mice were obtained from the Jackson Laboratory (B6.129S6(Cg)‐Spp1tm1Blh/J, JAX:0 04936). Both strains were maintained in a pathogen‐free animal facility at CSU, following standard purification procedures. The mice were housed in the laboratory animal unit, subjected to a 12‐hour day/night cycle, and granted ad libitum access to food and water. Before the surgical procedure, the mice were allowed one week to acclimate to the new environment. Healthy mice with normal growth and development were randomized for the experiment. The grouping of mice is blind to the experimenters and data analysts.

### Spinal Cord Crush Injury

All procedures were conducted following intraperitoneal injection of 0.4‐0.6 mL 0.3% sodium pentobarbital. Before surgery, the skin was prepared and disinfected, with the back area appropriately draped. A central incision was made in the skin at the T10 spinous process, and the muscles adjacent to the spinous process were separated. Subsequently, a laminectomy was performed at T10 to expose the spinal cord. The spinal cord was crushed using No. 5 Dumont forceps (Fine Science Tools), fixed on a stereotaxic apparatus, maintaining pressure for 3 s, as previously described.^[^ ^63^ ^]^ The SCI model was successfully established upon observing a local hematoma in the spinal cord tissue, accompanied by twitching in the lower limbs and tail of the mice. Subsequently, a layered muscle, fascia, and skin closure were performed using 4‐0 sutures, with skin sterilization using iodine. The Sham group was included in each experiment, where mice underwent exposure to T10 laminectomy without crush injury. Post‐surgery, mice were placed on a heating pad to maintain body temperature until awakening. They were then housed in cages with 4–5 mice per cage provided with ample food and drinking water. To prevent infection, penicillin was administered via intramuscular injection twice daily for three days after the operation. Aspirin (20 mg kg^−1^ body weight, MCE) was dissolved in drinking water and administered to the mice to inhibit inflammatory spinal pain. Manual bladder expression was performed on the mice for seven consecutive days following the surgery.

### Bioinformatic Analysis of Public Transcriptomic Dataset

The single‐cell RNA sequencing data and annotation information for a mouse SCI were retrieved from the GEO database (https://www.ncbi.nlm.nih.gov/geo/, accession number **GSE162610**). The spatial transcriptomic data and annotation information for a mouse SCI were retrieved from the GEO database (accession number **GSE195783**).

Using R software version 4.2.1, The Illumina output data were processed into a gene‐cell count matrix using the CellRanger software (v 2–4). Data processing and analysis were conducted using the R software package Seurat (v 3). Genes expressed in fewer than 10 cells were excluded. Subsequently, the gene expression matrix underwent normalization and scaling. Principal component analysis (PCA) was performed based on 3000 variable genes, and the top 20 principal components were selected. Batch effects were corrected using the “harmony” algorithm. The FindNeighbors() and FindClusters() functions were employed for dimensionality reduction and cell clustering. UMAP plots were generated to visualize annotated cell subpopulations. The FindAllMarkers() function was used to calculate marker genes for each cell type, comparing differential gene expression between cell subpopulations using the Wilcoxon rank‐sum test. Volcano plots were created with the ggplot2 package (v 3.3.6) to display diff58erentially expressed genes with a significance threshold of |Log_2_FC|>1 and P‐value<0.05. Featureplot() and Vlnplot() were used to depict gene expression distributions in cell subpopulations through scatter plots and violin plots, respectively. Cell stemness scores were calculated for different cell subpopulations using the AddModuleScore() function based on cell stemness gene sets obtained from the GSEA database. Cell trajectory analysis was performed using the CytoTRACE (v 0.3.3) and Monocle (v 2) packages to infer the differentiation degree and direction of cell subpopulations. GO functions and KEGG signaling pathways were enriched using the clusterprofiler package (v 4.4.4). Cell communication analysis reflecting ligand‐receptor interactions between cell subpopulations was conducted using the cellchat (v 1.5.0) and cellphoneDB (v 2) tools. The spatial distribution map of Opn RNA expression was generated through analysis using the R package “Seurat.”

### Isolation and Culture of Spinal Cord Microvascular Endothelial Cells (SCMECs)

The primary mouse SCMECs were isolated with modifications based on previous experiments.^[^ ^64^ ^]^ Briefly, 8‐week‐old female C57BL/6 mice were euthanized by cervical dislocation, and the spinal cords were carefully dissected under a microscope, with the removal of the dura mater and associated large blood vessels. The tissue was minced, and centrifuged, and the supernatant was discarded. Low‐glucose DMEM (Gibco) containing 0.1% (v/v) collagenase type II (Sigma) was added, and the tissue was digested on an incubator shaker for 1.5 h at 37 °C and 210 rpm. BSA gradient centrifugation was used to remove the myelin from the cells.^[^ ^64^ ^]^ After centrifugation at 1000 rpm for 8 min, the pellet was collected, and 20 ml of sterilized BSA‐DMEM (20%, w/v) was added. The mixture was centrifuged at 4400 rpm for 22 min at 4 °C to remove myelin. After centrifugation, the sample was separated into three layers. The visible white lipid layer in the middle is rich in myelin, and the cells are located at the bottom layer. The supernatant and the lipid layer in the middle were discarded, and 0.1% collagenase/dispase (Sigma) was added for an additional one‐hour digestion on a constant temperature shaker at 37 °C and 210 rpm. Finally, after centrifugation at 1200 rpm for 5 min, the pellets were washed twice in DMEM before being seeded on a collagen‐coated (Sigma) flask and cultured in the endothelial cell‐specific culture medium (10% FBS, Gibco; 50 µg mL^−1^ FGF‐basic, Peprotech) for further experiments. The SCMECs were examined with CD31 IF and assessed for cell purity by flow cytometry stained with DAPI, CD45, GLAST, PDGFRβ, CD31, and TIE2 (Figure S10, Supporting Information).

### Immunofluorescence

For the in vivo IF assay, 16‐µm thick frozen sections were obtained from the spinal cord, encompassing the lesion site. These sections were cut along the sagittal plane using a freezing microtome (Thermo Fisher). Following a 15‐min rewarming period at room temperature, the sections underwent three 10‐min rinses with a PBS solution. The spinal cord sections on the slides were delineated using an immunohistochemical pen. Subsequently, the sections were permeabilized with 100 µL of a PBS solution containing 0.3% Triton‐X 100 (Solarbio) for 30 min and blocked with 5% BSA (BioFroxx) in PBS for an additional 30 min. After blocking, the sections were incubated overnight at 4 °C with primary antibodies. Following 10‐min rinses with PBS containing 0.1% Tween 20 (Solarbio) (PBST) five times, the sections were incubated for one hour at room temperature with species‐appropriate secondary antibodies conjugated with Alexa Fluor fluorescent dyes (Abcam). Following an additional rinse with PBST five times, the sections were mounted on slides and covered with DAPI (GeneTex).

For the in vitro IF assay, cells were first cultured on cell climbing sheets at the desired density. Before staining, the culture medium was removed, and cells were washed three times with PBS. After fixing with 4% formaldehyde (PFA) (Solarbio) for 15 min, cells were washed again with PBS three times. Subsequently, the experiment was conducted following the consistent procedures of permeabilization, blocking, and incubation with primary and secondary antibodies as in the in vivo IF assay. Finally, the cell climbing sheets were covered with DAPI (GeneTex) and mounted on glass slides.

The slides were imaged under a fluorescence or confocal microscope (Zeiss). To validate antibody specificity and distinguish genuine target staining from the background, secondary antibody‐only controls were employed. For all IF images that do not show the entire spinal cord tissue, multiple images were randomly obtained from the central region of the injured spinal cord (as illustrated in Figure 1B) and showed the representative one. The ImageJ software was used for quantitative analysis of the images. The image is first split into channels and then converted to 8‐bit format. The same threshold range was applied for threshold adjustment in the same experimental batch. Colocalization analysis is used to analyze fluorescence colocalization across different channels. Following this, fluorescence intensity, intensity ratios, or the number of positive cells above the threshold intensity are measured. The Imaris 9.0 software was used for 3D reconstruction.

### Vasculature Microfil Perfusion, Synchrotron Radiation Micro‐Computed Tomography (SRµCT) Imaging, and Data Processing

Mice weighing 20–25 g and aged 8 weeks were anesthetized with 0.3% sodium pentobarbital (0.4‐0.6 mL) via intraperitoneal injection. Following anesthesia, the left ventricle was perfused with physiological saline, and then 9.45 mL of Microfil contrast agent (Flow Tech Inc) was infused into the vasculature for over 30 min, as per the manufacturer's instructions (5 mL diluent, 4 mL contrast agent, and 0.45 mL curing agent). After allowing the contrast agent to solidify, the specimens were harvested, fixed in 4% PFA, dehydrated in a gradient of ethanol (75%, 85%, 95%, and 100% for 4 h each), and subsequently immersed overnight in methyl salicylate (Macklin). Three mice were used for each experimental group.

The scanning procedure was conducted at the BL13W1 beamline of the Shanghai Synchrotron Radiation Facility (SSRF), utilizing a micro‐CT apparatus. The spinal cord samples were placed in a glass tube, immersed in fixed on the sample stage, and examined using SRµCT. X‐rays generated from an electron storage ring with an accelerated energy of 15 keV were employed for the measurements. The beam size was ≈45 mm (horizontal) × 5 mm (vertical), and a double‐crystal monochromator, with Si (111) and Si (311) crystals, was utilized to monochromatize the X‐rays. Upon penetrating the sample, X‐rays were converted into visible light by a cleaved Lu2SiO5:Ce single‐crystal scintillator. Projections were magnified by diffraction‐limited microscope optics (×10 magnification for neuronal network visualization) and digitized with a high‐resolution detector (ORCA Flash 4.0 Scientific CMOS, Hamamatsu K.K., Shizuoka Prefecture, Japan) with a physical pixel size of 3.25 µm for vasculature detection. The samples underwent continuous rotation during scanning, and 720 projection images were captured with an angular step size of 0.25° over 180° of rotation. The exposure time for each projection image was set to 600–1000 ms. The distance between the detector and the sample was adjusted to 3 cm. Flat‐field images with X‐ray illumination on the beam path without samples and dark‐field images with X‐ray illumination off were also collected during each acquisition procedure to correct electronic noise and brightness variations.

For 3D image reconstruction and quantitative analysis, the projected tomographic images were reconstructed using PTIRE (Phase‐sensitive X‐ray Image processing and Tomography Reconstruction, V3.5) developed by the SSRF to perform a direct filtered back‐projection algorithm. Subsequently, all 2D spinal cord slices were processed using Amira software (version 6.01, FEI, USA) to obtain the reconstructed 3D volumes. In the process of image processing in Amira, a 3D image was first obtained through volume rendering. Then, a 600 × 600 × 600 voxel subvolume was extracted from either the sham or injury group spinal cord center. Using interactive thresholding on each xy plane, a uniform CT threshold for all data was selected. Next, the Labeling function was used to mark all vessel segments with different colors, remove small non‐vessel voxel particles with the Filter by measure, and overlay the filtered dataset with the original dataset using Multi by image to complement and reconstruct details. Finally, the image type was converted to 8‐bit and performed Filament reconstruction and analysis to calculate the vasculature's nodes, segments, and total curved length (Figure S11, Supporting Information).

### Western Blotting

Cellular and tissue proteins were extracted using RIPA buffer (Elanscience) infused with protease and phosphatase inhibitors (Beyotime). Nuclear proteins were extracted using the Nuclear and Cytoplasmic Protein Extraction Kit (Beyotime) following the manufacturer's instructions. For spinal cord tissue, a 0.5 cm segment centered around the injury site was collected. The spinal cord tissue was collected following excision of the dura mater and major blood vessels and then homogenized for subsequent RIPA lysis. The samples were lysed in RIPA buffer at 4 degrees for 30 min, followed by centrifugation at 12 000 g to collect the protein supernatant. The protein concentrations were measured using the BCA Assay Kit (Elanscience). Next, the proteins were denatured at 95 °C for 10 min and loaded with the loading buffer. Subsequently, the proteins were separated using SDS‐PAGE gels and transferred onto a polyvinylidene fluoride membrane (Millipore, Billerica, MA). The membrane was blocked with 5% milk in TBST for 60 min at room temperature before incubation with primary antibodies overnight at 4 °C. After rinsing five times with TBST (Solarbio), the membrane was incubated with peroxidase‐conjugated goat anti‐rabbit or anti‐mouse IgG secondary antibodies (Elanscience). Finally, immunoreactive bands were visualized using the chemiluminescence reagent (ShareBio) with a ChemiDoc XRS Plus luminescent image analyzer (Bio‐Rad, England). Image analysis was performed using ImageJ software. The relative expression levels of P‐Foxo1, p‐Smad2/3, p‐mTOR, and p‐GSK3β were determined by comparing them to the expression levels of their total proteins (Foxo1, Smad2/3, mTOR, and GSK3β, respectively). The relative expression level of nuclear Foxo1 was determined by comparing it to the expression level of HH3, while the expression levels of all other proteins were determined by comparing them to β‐actin.

### Evans Blue Leakage Assay and Light‐Sheet Microscopy Imaging

The permeability of the BSCB was assessed using EB dye extravasation assay, as previously described.^[^ ^10^ ^]^ A volume of 0.1 mL of 2% EB (Sigma) in saline solution was injected through the tail vein at 28 dpi. After one hour, mice were anesthetized, and the heart was perfused with 0.9% saline and 4% paraformaldehyde for fixation. Subsequently, mice were dissected to obtain complete spinal cord specimens. They were dehydrated in a 20/40/60/80/100% methanol solution for one hour each. Samples were dehydrated overnight in a 100% methanol solution. The following day, they were first washed twice with dichloromethane and then oscillated and incubated in dichloromethane at room temperature. Finally, they were incubated overnight in dibenzyl ether to clear the tissue to be completely transparent. A 3D fluorescence image of the entire tissue was obtained under a light microscope (Miltenyi, Germany) with a 4 × objective lens in dibenzyl ether medium. Quantitative analyses of the EB‐positive volume were conducted using the Imaris 9.0 software.

### Cell Treatment

The extracted primary BMDMs from WT or OPN KO mice were seeded in six‐well plates at 10^5 cells per mL density. The cells were treated with 100 ng mL^−1^ of interferon‐γ (Peprotech) to induce the M1 pro‐inflammatory phenotype of macrophages. After 48 hours, the supernatant containing cytokines was discarded, fresh culture medium was added, and the cells were cultured for an additional 48 h. The CM was then collected for endothelial cell treatment, with a duration of 24 h. Additional recombinant OPN protein (2 µg mL^−1^) was added with the CM from WT BMDMs.

For SCMECs and bEnd.3 treatment, cells were seeded in six‐well plates at 10^5 cells/ml density. In all experiments, recombinant OPN protein (2 µg mL^−1^) and/or TGF‐β1 (30 ng mL^−1^) (R&D systems) were administered for 72 h. Furthermore, Cilengitide (5 µg mL^−1^), Wortmannin (20 nM) (MCE), Sarmentosin (10 µM) (BioBioPha) or other inhibitors were introduced for an additional 48 h after 24 h of OPN induction. The Foxo1 siRNA was designed with siDirect version 2.1. and synthesized by Shanghai Sangon Biotech. Three top‐ranking sequences were selected and transfected into SCMECs using Lipofectamine 2000 (Thermofisher). The sequence with the best knockdown effect was chosen for co‐treatment with OPN.

### RNA Extraction, Library Preparation, and Sequencing

RNA‐seq experiment and high throughput sequencing and data analysis were conducted by Seqhealth Technology Co., LTD (Wuhan, China). The first‐generation SCMECs from primary culture were treated separately with PBS or 2 µg mL^−1^ of recombinant OPN protein for 72 h (two million cells per group). Total RNAs were extracted from cells using TRIzol Plus RNA Purification Kit (Invitrogen). DNA digestion was carried out after RNA extraction by DNaseI. RNA quality was determined by examining A260/A280 with a Nanodrop spectrophotometer (Thermo Fisher). RNA Integrity was confirmed by 1.5% agarose gel electrophoresis. Qualified RNAs were finally quantified by Qubit3.0 with a QubitTM RNA Broad Range Assay kit (Life Technologies, Q10210).

A total of 2 µg of total RNAs was utilized to perform stranded RNA sequencing library preparation employing the KCTM Stranded mRNA Library Prep Kit for Illumina (Catalog NO. DR08402, Wuhan Seqhealth Co., Ltd. China), following the manufacturer's instructions. The 200–500 bps range PCR products were subjected to enrichment, quantification, and subsequent sequencing on a DNBSEQ‐T7 sequencer (MGI Tech Co., Ltd. China) with the PE150 model. The initial raw sequencing data underwent filtration using Trimmomatic (version 0.36), whereby low‐quality reads were discarded, and sequences contaminated with adaptor sequences were trimmed. The resulting clean data were aligned to the mouse reference genome obtained from the UCSC Genome Browser (https://genome.ucsc.edu/) using STRA software (version 2.5.3a) with default parameters. Read counts corresponding to the exon regions of each gene were determined using featureCounts (Subread‐1.5.1; Bioconductor).

### RNA‐Seq Data Analysis

Using R software version 4.2.1, differential analysis was performed on the raw count's matrix with DESeq2 (v 1.36.0) and edgeR (v 3.38.2). The analysis followed standard procedures, and the raw count matrix was normalized using the VST (Variance Stabilizing Transformations) method provided by the DESeq2 package. For visualization of the differential analysis results, PCA analysis and volcano plots were generated using ggplot2 (v 3.3.6). Molecular ID conversion was carried out using the org.Hs.eg.db library, and clusterProfiler (v 4.4.4) was employed for GSEA analyses (based on the MSigDB Collections dataset) and Gene ontology (GO)/KEGG analysis. The Fragments Per Kilobase of transcript per Million mapped reads (FPKM) for gene expression, representing the transcription per million mapped fragments per kilobase, is calculated from the gene counts (RCg) using the following formula: FPKM = RCg*10^9/(RCpc*L)

Here, RCg refers to the number of reads mapped to the gene, RCpc represents the reads mapped to all protein‐coding genes, and L denotes the gene length, defined as the sum of exon lengths. To facilitate the visualization of gene expression patterns, the gene expression values are scaled to Z‐scores (with a mean of 0 and a variance of 1), generating a gene expression heatmap.

### Quantitative Real‐Time Polymerase Chain Reaction (qRT‐PCR)

Total RNA of cells was extracted using the TRIzol Plus RNA Purification Kit (Invitrogen), and the GoScript™ Reverse Transcription System (Promega Corporation) was employed for single‐strand cDNA synthesis using 1 µg total RNA from each sample. For qRT‐PCR, 2x SYBR Green qPCR master mix (Bimake) was utilized, and all reactions were conducted and analyzed on an RT‐PCR System (ABI 7900 fast real‐time PCR system, Applied Biosystems). The expression levels of target genes were normalized to GAPDH, and the relative gene expression was calculated using the 2**^–ΔΔCT^** method. Primer sequences for qRT‐PCR are provided in Table S1 (Supporting Information).

### Dual Luciferase Assay

The Mouse Foxo1 overexpression plasmid (CMV enhancer‐MCS‐3FLAG‐SV40‐Puromycin), Mouse Smad7 promoter‐luciferase reporter plasmid (MCS‐firefly_Luciferase), and negative Control (NC) plasmid were synthesized (GeneChem Biology, Shanghai, China). bEnd.3 cells, a mouse brain endothelial cell line, or SCMECs were co‐transfected with the Foxo1 overexpression plasmid and/or Smad7 promoter‐luciferase reporter plasmids using Lipofectamine 2000 (Thermofisher). SCMECs treated with OPN and/or Sarmentosin were only transfected with luciferase reporter gene plasmids. After 48 hours of transfection, the luminescence intensity ratio of Firefly Luciferase/Renilla Luciferase in bEnd.3 cells or SCMECs in each group were determined using the double luciferase reporter gene detection kit (Promega Corporation) following the manufacturer's instructions. The average Firefly/Renilla luciferase ratio of the control group was first calculated, and the ratios in all experimental groups were normalized based on their comparison to the control group's average value.

### ChIP‐qPCR

ChIP assays were conducted using the BeyoChIP™ ChIP Assay Kit (Beyotime). Following the manufacturer's instructions, protein samples were obtained, and antibodies against Foxo1 and IgG were used to immunoprecipitate the target protein. Protein A/G Magnetic Beads/Salmon Sperm DNA was employed to precipitate the protein complexes recognized by the primary antibody. After separation on a magnetic rack and elution, DNA products were obtained using a DNA purification kit (Beyotime) for subsequent qRT‐PCR analysis. Three primers targeting the Smad7 gene promoter were generated and validated using NCBI Primer‐BLAST and were employed in qRT‐PCR experiments (Table S1, Supporting Information). Values obtained from ChIP‐qPCR were normalized to the 1% input of total DNA. The adjusted input value was calculated by subtracting the dilution factor (log_2_100) from the 1% input CT value. The percent input of samples from Foxo1 or anti‐rabbit IgG control was calculated as 100 × 2^(adjusted input‐CT_(IP)_).

### In Vitro Trans‐Endothelial Permeability Assay

To assess trans‐endothelial permeability, 40 000 MW FITC‐dextran (1 mg mL^−1^; Sigma) was employed. SCMECs were seeded at a density of 2 × 10^5 cells mL^−1^ in the upper chamber of 24‐well transwell inserts with 0.4‐µm filter membranes (Corning) and cultured until reaching confluence. Following various treatments, the upper chamber was filled with FITC‐dextran, while PBS was placed in the lower chamber. After 1 h in the dark, 40 µL of medium from the lower chamber was collected, and the fluorescence intensity at 520 nm was measured using a microplate reader (Thermo Fisher Scientific, USA). The negative control refers to the group using PBS, and the positive control refers to FITC‐dextran initially added to the upper chamber. The transport of FITC‐dextran was calculated as follows: Transport (%) = (Fluorescence intensity (FI) ^lower chamber medium^ – FI ^Negative control^) / FI^Positive control^.

### CCK‐8 Assay

Each well of a 96‐well culture plate was loaded with 100 µl of bEnd.3 cell suspension (5 × 10^3 cells) and subjected to various drug treatments (0, 1, 2, 4, 8, 16, 32, 64, 128, 256, 512, 1024 µM) or vehicle controls. Three wells were designated for each experimental group, and a cell‐free group served as the blank. Following incubation in complete media for 24 and 48 h, 10 µL of Cell Counting Kit 8 Reagent (Beyotime) was added to each well. After a 2‐hour incubation at 37 °C, the absorbance was measured at 450 nm using a microplate reader (Thermo Fisher Scientific, USA). Cell viability was calculated by normalizing the average absorbance of groups without drug treatment to 1.0 and then comparing the ratios of all experimental groups to the control group's average value.

### Construction and Evaluation of Aptamer‐Liposome Encapsulated Sarmentosin

Soybean phosphatidylcholine (SPC), cholesterol, and DSPE‐PEG‐2K were dissolved in 3 mL of chloroform. The solution was vacuum‐evaporated in a sample vial to form a film. Sarmentosin, dissolved in deionized water, was added to the sample vial. The mixture underwent ultrasonication (JP‐020, Shenzhen Jiemeng, China) and extrusion through a liposome extruder (Mini‐extruder, Avanti, USA, polycarbonate membrane, pore size 100 nm). The nanodispersion was dialyzed using a nanoporous membrane (polycarbonate membrane, pore size 30 nm) to remove unloaded Sarmentosin (ND‐1, Xi'an Ruixi, China). Finally, deionized water was added to adjust the volume to 4 mL. Subsequently, A total of 500 nmol of aptamer (CA2, 5′ (Cy5)‐CCCACGTCTGCGCTTAGCTCCTGGGCCTGGATGGGC‐3′cholesterol, Sangon Biotech, Shanghai, China) conjugated with cholesterol was mixed with the liposome solution (encapsulating 10 mg of Sarmentosin) on a shaker at room temperature for 2 h to facilitate their binding with liposomes. The resultant solution was lyophilized with a freeze‐drying protectant, yielding a final product of 25 mg of lyophilized powder. Particle size distribution, Zeta potential measurements, and PDI were determined using the Brookhaven Instruments NanoBrook 90plus PALS Nanoparticle Size and Zeta Potential Analyzer (Brookhaven, USA).

The encapsulation efficiency and drug release curve were determined by UV spectroscopy (TU‐1810, Beijing Puxi, China). At specific time points (0 h, 1 h, 2 h, 4 h, 6 h, 24 h, 48 h), the liposome dispersion is extracted from the dialysis bag. The dispersion was treated with Triton X‐100 to disrupt the liposome structure and release the encapsulated Sar. The aqueous phase was then measured for absorbance using UV spectroscopy at the maximum absorption wavelength of 340 nm. The concentration of Sarmentosin in the samples was calculated using a standard curve based on the peak area of the standard Sar sample.

### Transmission Electron Microscopy

The freeze‐dried powder was fixed in 2.5% glutaraldehyde for 4 hours. Specimens were washed with Millonig's phosphate buffer solution (pH = 7.3), treated with 1% osmium tetroxide for one hour, and washed again. After dehydration in acetone gradients (50%, 70%, and 90% for 10 min each, followed by two rounds of 100% acetone at 15‐min intervals), specimens were soaked in a 1:1 mixture of acetone and resin for 12 h. They were then embedded in 100% resin at 37 °C overnight. Once solidified, specimens were polymerized at 37 °C overnight and then at 60 °C for 12 h. Ultrathin sections were obtained using a UC‐7 ultramicrotome (Leica) with a diamond knife. Finally, sections were double‐stained with 3% uranyl acetate and lead nitrate and examined and photographed using an electron microscope (TF20, FEI, USA).

### In Vivo Imaging and Tracing

At 24 hours post‐SCI, mice were intravenously injected with 200 µL of control vehicle (saline), Lipo@Sar (100 mg kg^−1^, 1 µL DiR‐labeled), or Apt‐LP@Sar (100 mg kg^−1^, 1 µL DiR‐labeled). Imaging was conducted at 24 and 48 h post‐injection using the IVIS Spectrum instrument (PerkinElmer, Germany) with excitation at 754 nm and emission at 778 nm. Fluorescence intensity was statistically processed and quantified using the IVIS Living Image software. All data were imported together for analysis, with the signal threshold uniformly set to 3.73 × 10^7 to obtain representative images. The total radiant efficiency was statistically analyzed by selecting the same area of ROI at the injury site.

For in vivo tracing of aptamer‐labeled liposomes, Cy5 was synthesized at the 5′ end of the aptamer. At 24 h post‐spinal cord injury (SCI), mice were intravenously injected with 200 µL Apt (Cy5)‐LP@Sar (100 mg kg^−1^). Tissues were collected for Representative IF images with CD31, F4/80, Iba1, GFAP, and NeuN.

### Animal Treatment

The experimental procedure for animal studies is illustrated in Figure 7A. Eight‐week‐old female and male mice weighing between 20–25 g were subjected to SCI. At 1, 3, 5, and 7 dpi, intravenous injections of 200 µL saline and Apt‐LP@Sar (100 mg kg^−1^) were administered. 4 mice per group were used for CST tracing, three mice per group were used for imaging with the light‐sheet microscope after EB injection, 6 mice per group were used for various IF assays and neuroelectrophysiology, and 10 mice per group were used for neurobehavioral assessments. No mice died during the experiment for Apt‐LP@Sar efficacy evaluation.

### Anterograde Tracing of the Corticospinal Tracts (CSTs)

Anterograde tracing of CSTs was performed using Alexa Fluor 488‐labeled Dextran (10000 MW, Invitrogen), following established protocols.^[^ ^13^ ^]^ Two weeks post‐SCI, mice were anesthetized with 0.4‐0.6 mL of 0.3% pentobarbital sodium. Subsequently, the mice were secured in a stereotactic instrument, and following exposure of the skull, the portion above the sensorimotor cortex was removed using a dental drill. Four microinjections (0.4 µL per injection) of Alexa Fluor 488‐labeled Dextran were precisely targeted to layer V in the sensorimotor cortex (two injections per hemisphere) at the following coordinates from bregma: Anteroposterior ± 0.5 mm, Mediolateral ± 1 mm, Dorsoventral 0.55 mm. The injections were administered at a 0.1 µL min^−1^ rate using a 10 µL syringe equipped with a 36 G beveled needle tip. The needle was kept in place for 5 min after each injection to ensure sufficient infiltration. Each group consisted of four animals that underwent CST tracing. The main CST fiber length was quantified by measuring the continuous length of CST fibers extending past the lesion border. The CST area was determined by calculating the proportion of CST‐positive area within a 300 µm^2^ region centered around the lesion border.

### Neuroelectrophysiology

Electromyography was conducted on 28 dpi to assess the motor‐evoked potentials (MEPs) of the mice. Intraperitoneal injection of 0.4‐0.6 mL of 0.3% sodium pentobarbital was administered for anesthesia. A positive stimulating electrode was positioned over the motor area of the cerebral cortex's skull surface, with a negative stimulating electrode placed near the orbital bones. Additionally, a recording electrode was inserted into either the left or right gastrocnemius muscle, a reference electrode was placed in the distal tendon of the hind limb muscle, and a ground electrode was positioned under the back skin. Stimulation involved a 3‐mA single square wave (2 Hz) for 0.2 ms and hind limb amplitudes were recorded by measuring from the initiation point of the first response wave to the highest peak.

### BMS Score Evaluation and Swimming Test

For evaluating hindlimb motor function recovery in SCI mice, the BMS score system was utilized following established protocols.^[^ ^65^ ^]^ Assessments were conducted preinjury and on days 1, 3, 7, 14, and 28 dpi. BMS scores ranged from 0 to 9, with 0 representing complete paralysis and 9 indicating normal hindlimb movement function. Before scoring, mice were acclimated to the test environment by placing them on the test table, and each mouse was observed for 4 min.

LSS swim score was utilized to assess swimming ability, considering hindlimb movement, hindlimb alternation, forelimb dependency, trunk stability, and body angle, as previously described.^[^ ^66^ ^]^ Mice underwent a three‐day training period to swim in a water‐filled tank (5 × 15 cm) before surgery. Surgery was performed after this training, and on the 28th day post‐surgery, mice were observed in the same tank for one min.

Two independent and blinded observers simultaneously assessed and assigned the BMS and LSS scores to the mice. The final score was recorded as the average of the scores provided by the two observers.

### Statistical Analysis

All statistical analysis was conducted using GraphPad Prism (version 7.0, USA). The Robust Outlier Test (Q = 1%) was used to identify outliers from the original data. Results were presented as mean ± standard deviation (SD). The sample size (n) for each experiment is indicated in the figure legend. Normality was assessed using the Shapiro‐Wilk test (Shapiro‐Wilk for n < 5 and both Shapiro‐Wilk and Kolmogorov‐Smirnov for n ≥ 5). For two‐group comparisons, a two‐sided unpaired t‐test was employed after confirming that data were normally distributed and exhibited homogeneity of variances (checked by the F‐test). For multiple group comparisons, the Brown‐Forsythe test was used to assess the homogeneity of variances. After the data were checked to meet the criteria of normal distribution and homogeneity of variance, ordinary one‐way ANOVA followed by Tukey's post‐hoc multiple comparisons test was performed. Two‐way ANOVA followed by Tukey's post‐hoc multiple comparisons test was applied for analyses involving two variables. Statistical significance was set at p < 0.05. In figures, ns indicates p ≥ 0.05, * denotes p < 0.05, ** denotes p < 0.01, *** denotes p < 0.001, and **** denotes p < 0.0001.

## Conflict of Interest

The authors declare no conflict of interest.

## Author Contributions

L.J., J.H., and H.L. conceived and designed the research. Y.C., T.W., and C.D. supervised all aspects of the research and provided critical revisions. J.X., F.Y., Y.L., and Y.X. conducted cellular experiments. J.X., C.S., T.Q., and C.L. performed animal experiments. J.X. and Y.D. conducted bioinformatic analysis. J.X., C.S., and Y.D. performed statistical data analysis. J.X. and L.J. wrote and finalized the article.

## Supporting information



Supporting Information

Supporting Information

Supporting Information

Supporting Information

Supporting Information

## Data Availability

The data that support the findings of this study are available from the corresponding author upon reasonable request.
