# Supplementary material for: Endothelial Foxo1 Phosphorylation Inhibition via Aptamer‐Liposome Alleviates OPN‐Induced Pathological Vascular Remodeling Following Spinal Cord Injury
Source: Adv Sci (Weinh). 2024 Sep 28;11(43):2406398. doi: 10.1002/advs.202406398 (PMC11578346; doi:10.1002/advs.202406398)
Supplement: Supplementary file 1 — Supporting Information [file ADVS-11-2406398-s003.docx]

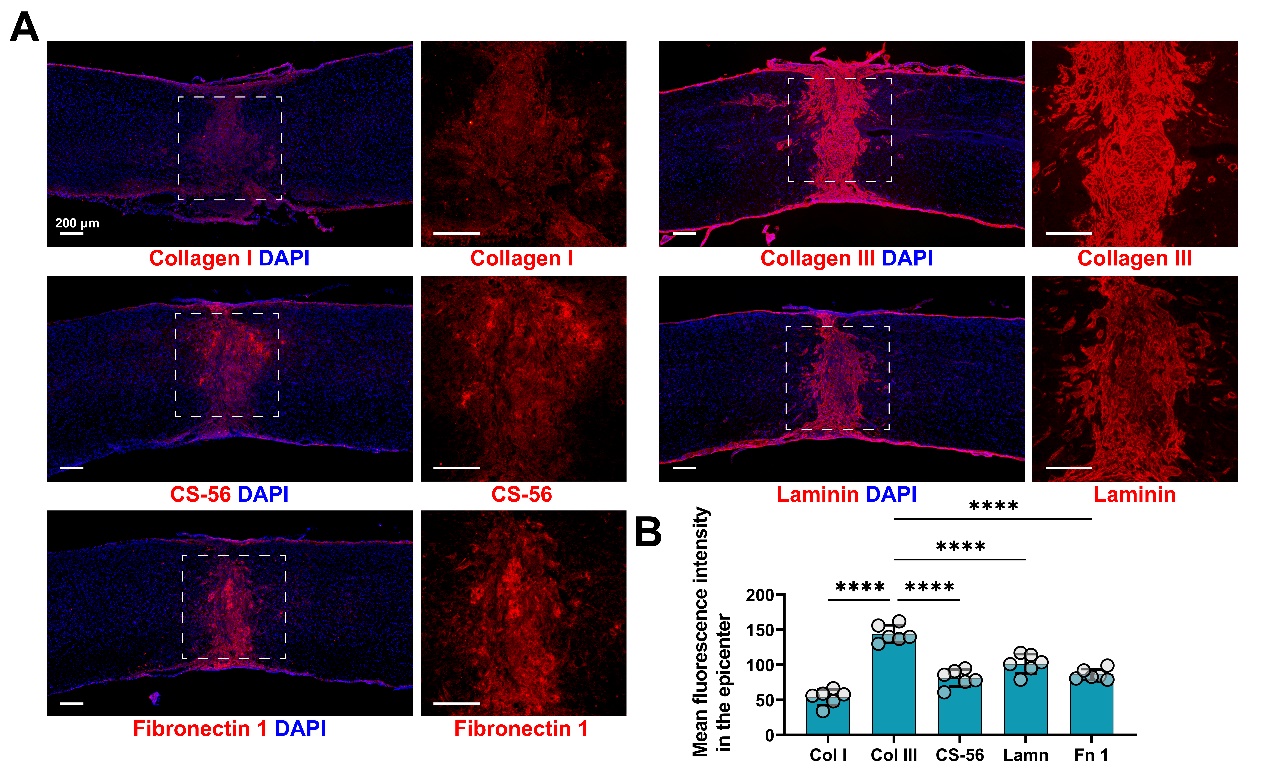
**Figure S1.** Extracellular matrix (ECM) deposition in the chronic phase of SCI. A) Representative confocal images of Collagen I/ Collagen III/CS-56/Laminin/Fibronectin 1 (red) DAPI (blue) of the spinal cord at 28 dpi. (scale bar = 200 μm, n=6) B) Quantitative analysis showing the mean fluorescence intensity of ECM proteins in the epicenter in (A). (n=6, mean ± SD, unpaired t-test) **** p<0.0001


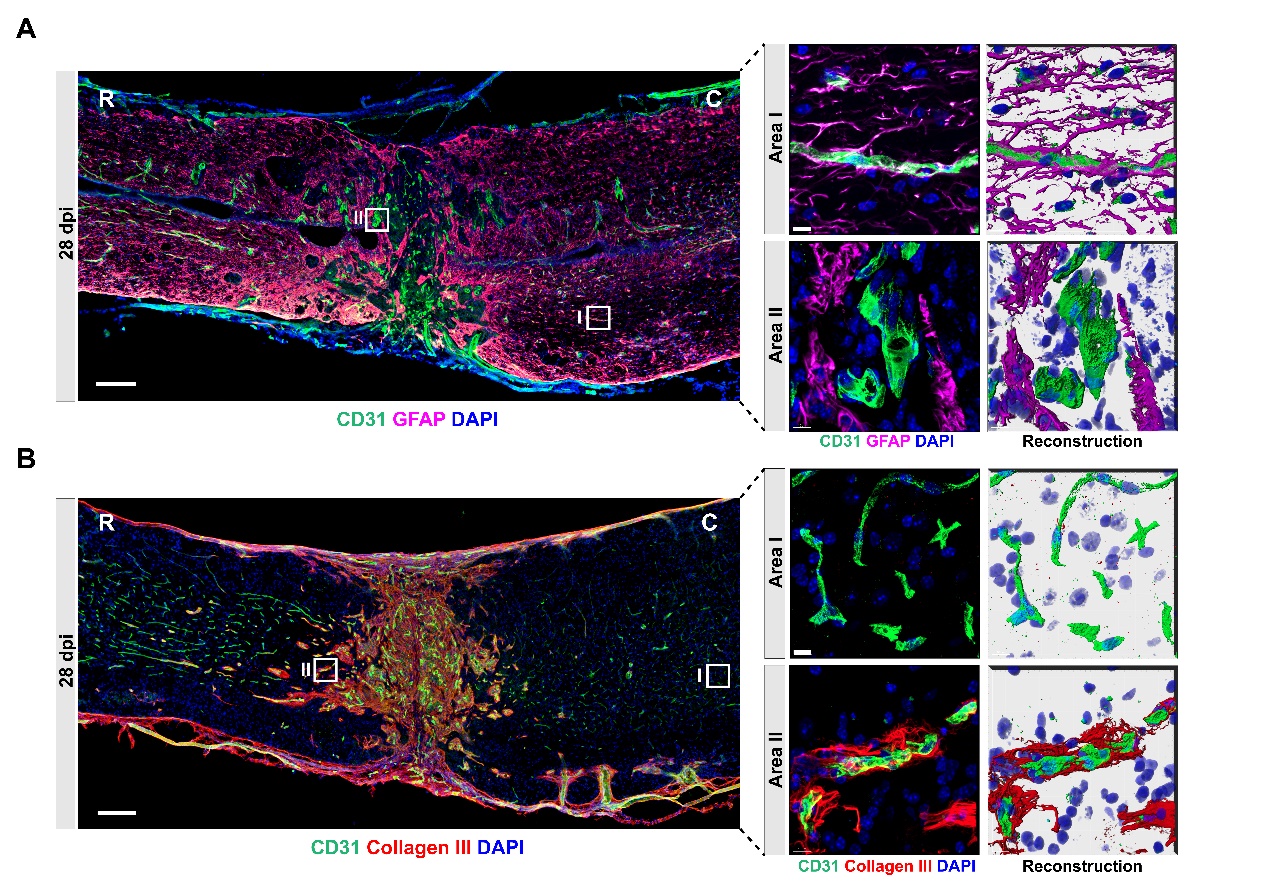
**Figure S2.** Traumatic pathological vascular remodeling in the chronic phase of SCI. A) Representative confocal images of CD31 (green), GFAP (pink), and DAPI (blue) and B) Representative confocal images of CD31 (green), Collagen III (red), and DAPI (blue) of the spinal cord at 28 dpi (scale bar = 200 μm), as well as the enlarged view of local vessels in Area I (injury epicenter) and Area II (uninjured area). (scale bar = 10 μm)


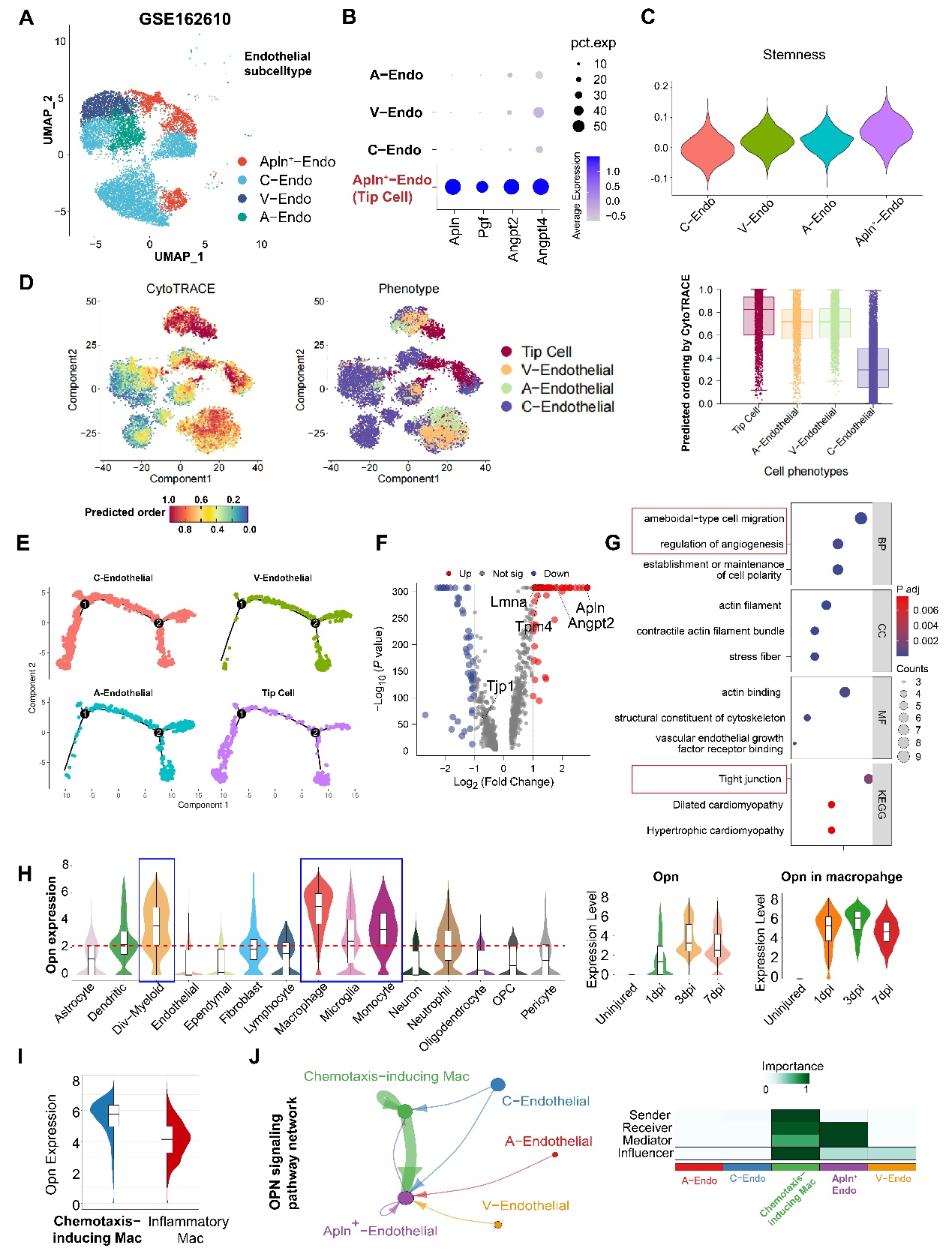
**Figure S3.** OPN**-**Apln^+^ endothelial tip cells interaction drive angiogenesis after SCI (GSE162610). A) UMAP plot showing clusters and celltype annotations of endothelial cells. B) Dot plot showing the average expression of cell markers in annotated celltypes. C) Scoring on the overall expression levels of stemness-related genes in different endothelial cell subtypes. D) Cytotrace analysis showing the degree of endothelial cell subtype differentiation, with predicted orders ranging from 0.0 to 1.0. Higher scores indicate lower differentiation levels. E) Cell trajectory analysis depicting the differentiation trajectory of endothelial cell subtypes. F) Volcano plot showing the DEGs between C-Endothelial and Apln^+^ Endothelial cells. G) GO and KEGG analysis of DEGs in (F). H) The expression level of Opn RNA in different cell types (left panel), different days post injury (dpi) (middle panel), and in macrophages of different dpi (right panel). I) The expression level of Opn RNA in chemotaxis-inducing macrophage and inflammatory macrophage. J) Cellchat analysis showing the OPN signaling pathway network between chemotaxis-inducing macrophage and different endothelial cell subtypes.


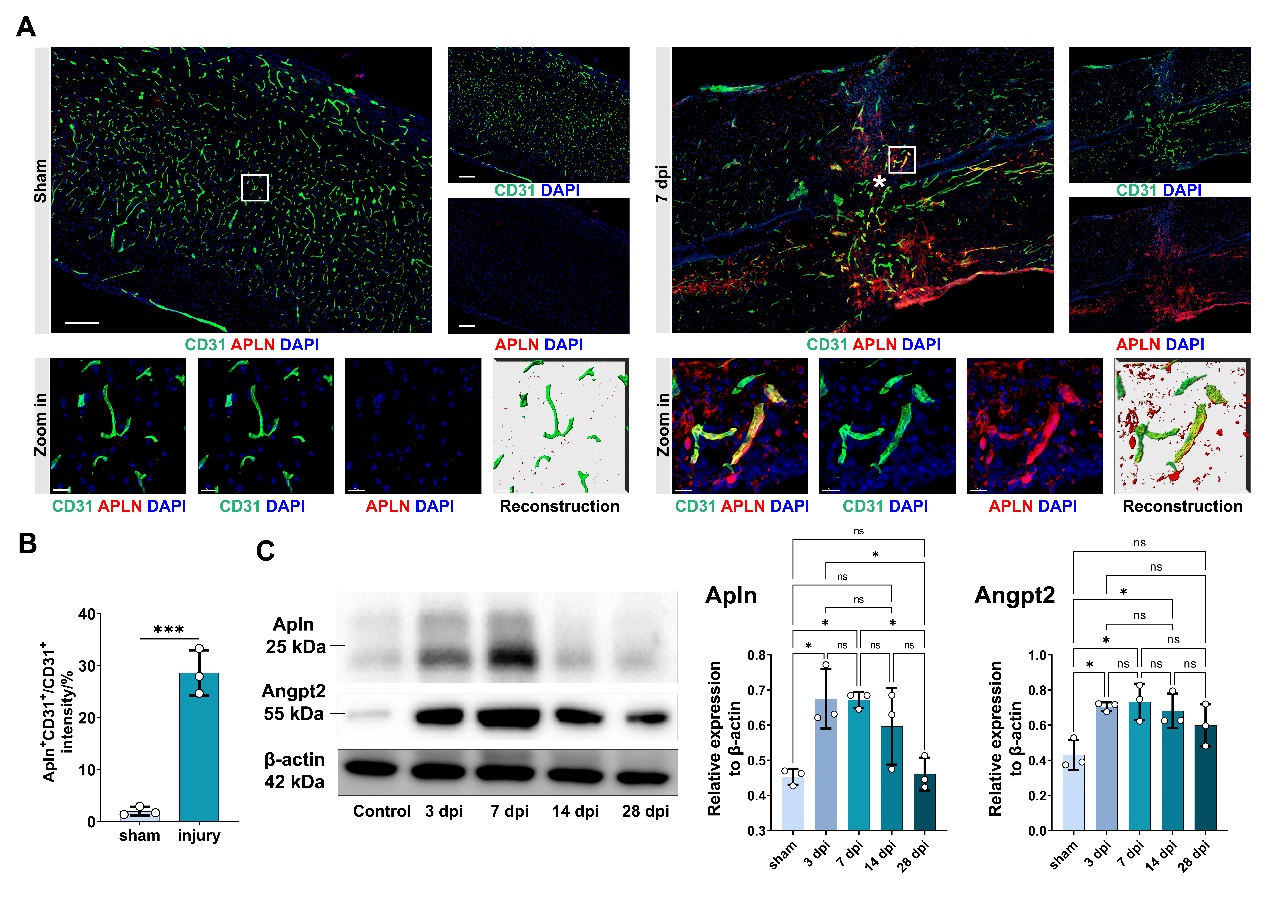


**Figure S4.** Apln expressed in newly formed endothelial cells after SCI. A) Representative confocal images of CD31 (green), Apln (red), and DAPI (blue) of the sham spinal cord and injured spinal cord at 7 dpi (Scale bar = 200 μm, n=3), as well as the enlarged view of the epicenter (white box). (Scale bar = 20 μm) B) Quantitative analysis of the Apln^+^CD31^+^/CD31^+^ fluorescence intensity in (A). (n=3, mean ± SD, unpaired t-test) C) Western blot images and quantitative analysis showing the relative protein expression levels of Apln and Angpt2 to β-actin in the sham group and at 3, 7, 14, and 28 dpi. (n=3, mean ± SD, one-way ANOVA, Tukey's multiple comparisons) ns not significant, ∗ p < 0.05, ∗∗∗ p < 0.001


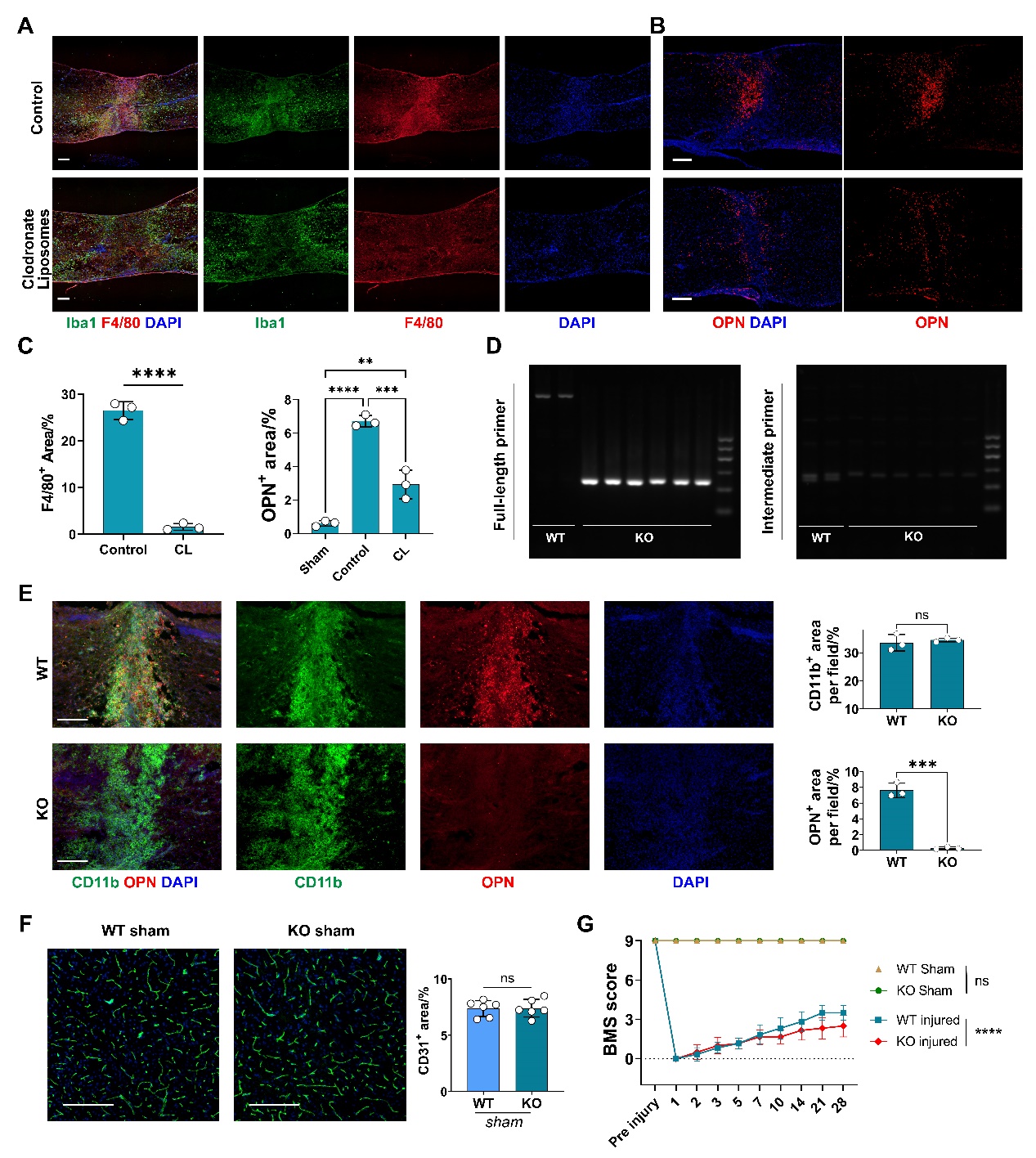


**Figure S5.** Establishment of OPN Knockout (KO) Mice. A) Representative IF images of Iba1 (green), F4/80 (red), and DAPI (blue) and B) Representative IF images of OPN (red), and DAPI (blue) at 7 dpi in mice administered with control liposomes and Clodronate liposomes. (Scale bar = 200 μm, n=3). C) Quantitative analysis of F4/80^+^ and OPN^+^ area in (A) and (B). (left: n=3, mean ± SD, unpaired t test; right: n=3, mean ± SD, one-way ANOVA, Tukey's multiple comparisons) D) Genotypic analysis results of tail DNA from wild-type (WT) mice and OPN KO mice. E) Representative IF images and quantitative analysis of CD11b (green), OPN (red), and DAPI (blue) in WT and OPN KO mice at 7 dpi. (Scale bar = 200 μm, n=3, mean ± SD, unpaired t test) F) Representative IF images and quantitative analysis of CD31 (green) in WT and OPN KO mice from the sham group. (Scale bar = 200 μm, n=3, mean ± SD, unpaired t test) G) Basso Mouse Scale (BMS) score over time post-injury in the sham and injury groups of WT and OPN KO mice (n=6, mean ± SD, two-way ANOVA, Tukey's multiple comparisons). ns not significant, ∗∗ p < 0.01, ∗∗∗ p < 0.001, ∗∗∗∗ p < 0.0001


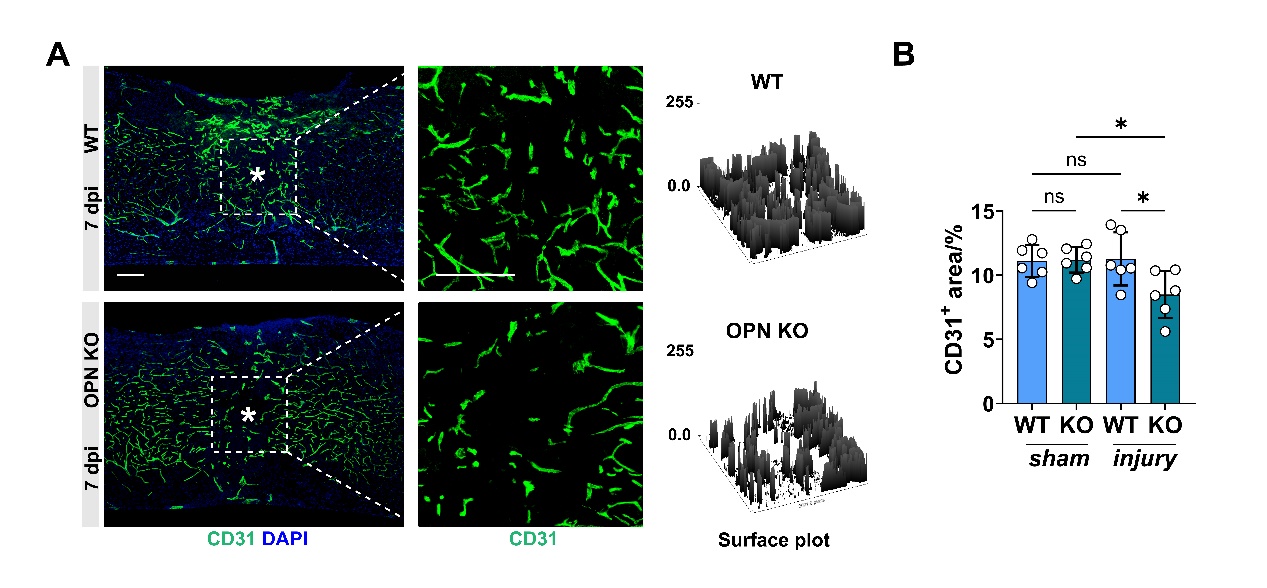
**Figure S6.** The OPN KO mice exhibit a reduction in vascular density after SCI. A) Representative IF images and surface plots of CD31 (green) and DAPI (blue) in WT and OPN KO mice at 7 dpi. (Scale bar = 200 μm, n=6) B) Quantitative analysis of CD31^+^ area in WT and OPN KO mice from the sham group or 7 dpi. (n=6, mean ± SD, one-way ANOVA, Tukey's multiple comparisons) ns not significant, ∗ p < 0.05


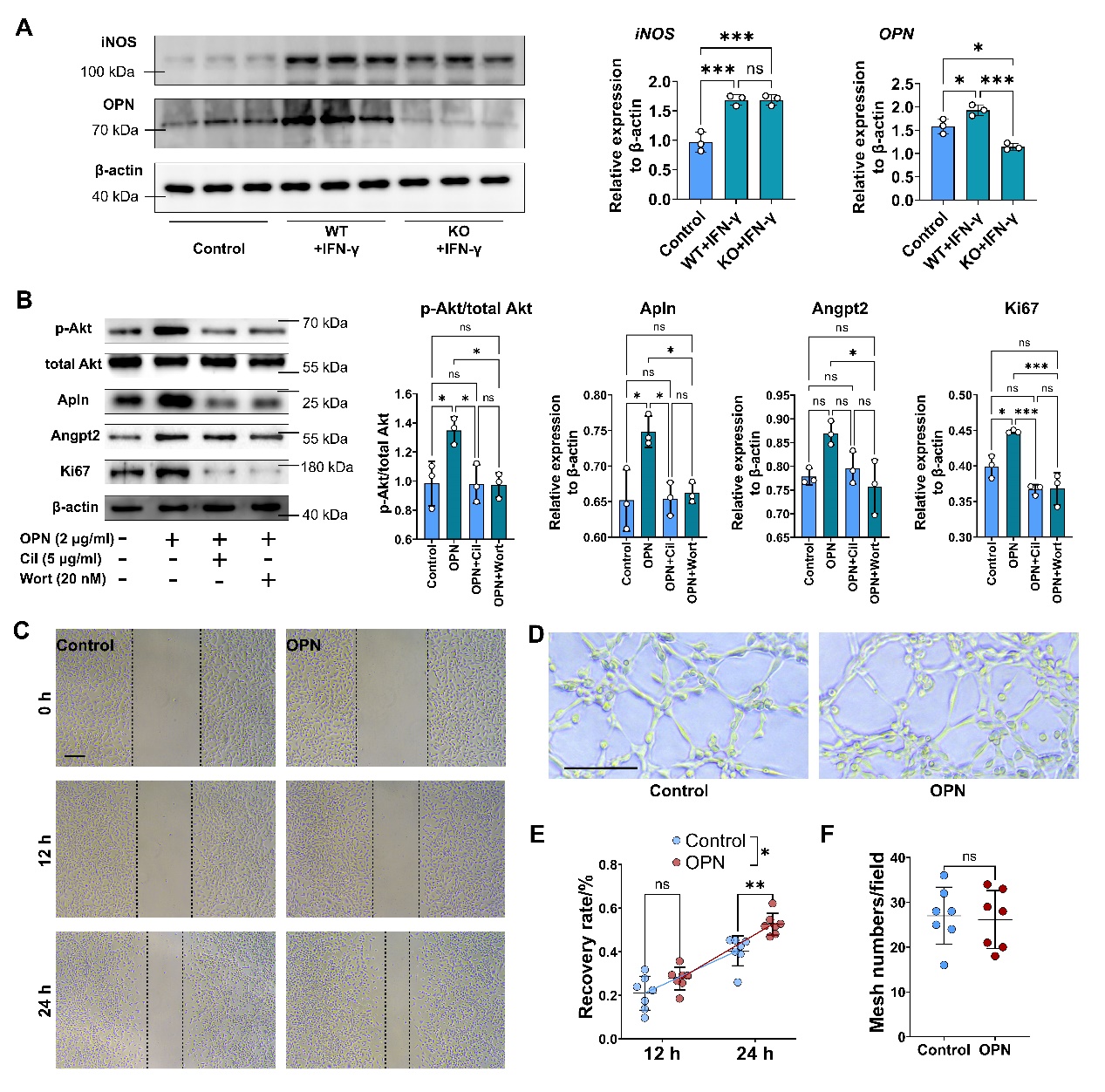
**Figure S7.** A) Western blot images and Quantitative analysis showing the relative expression level of iNOS and OPN to β-actin in the control group BMDMs, M1-polarized BMDMs induced by IFN-γ derived from WT mice, and M1-polarized BMDMs induced by IFN-γ derived from OPN KO mice. (n=6, mean ± SD, one-way ANOVA, Tukey's multiple comparisons) B) Western blot images and quantitative analysis showing the relative protein expression levels of p-Akt to total Akt, Apln and Angpt2 to β-actin in different treatment group of SCMECs. (n=3, mean ± SD, one-way ANOVA, Tukey's multiple comparisons) C) Representative bright-field microscopy images of bEnd.3 cells at 0h, 12h, and 24h demonstrating lateral wound migration. (Scale bar = 200 μm, n=7) D) Representative bright-field microscopy images of bEnd.3 cells after 12 hours of culture on Matrigel demonstrating tube formation. (Scale bar = 200 μm, n=7) E) Quantitative analysis of recovery rate in (C). (n=7, mean ± SD, two-way ANOVA, Tukey's multiple comparisons) F) Quantitative analysis of mesh numbers in (D). (n=7, mean ± SD, unpaired t test) ns not significant, ∗ p < 0.05, ∗∗∗ p < 0.001


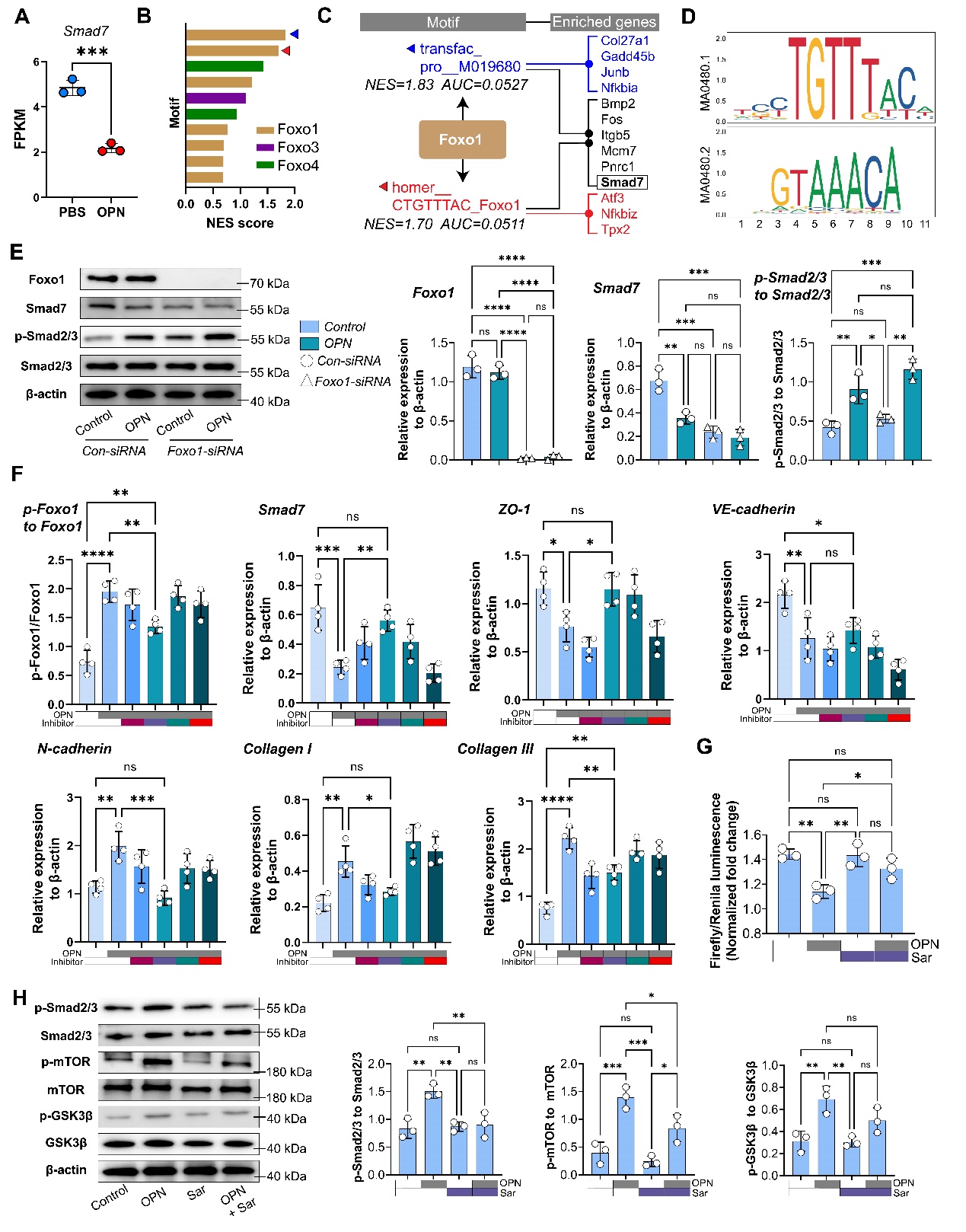
**Figure S8.** A) FPKM of Smad7 RNA with PBS or OPN treatment. B) The ranking of NES scores for the Foxo family transcription factor binding motifs corresponding to the top 200 DEGs after OPN treatment. C) Downstream DEGs corresponding to the two Foxo1 binding motifs. D) Sequences of the binding motifs of Foxo1 as shown in the Jaspar database. E) Western blot images and quantitative analysis showing the relative protein expression levels of Foxo1 and Smad7 to β-actin, p-Smad2/3 to Smad2/3 in different treatment group of SCMECs with OPN and siRNA. (n=3, mean ± SD, one-way ANOVA, Tukey's multiple comparisons) F) Quantitative analysis of the relative protein expression levels to Foxo1 or in Figure 6D. (n=4, mean ± SD, one-way ANOVA, Tukey's multiple comparisons) G) The normalized Firefly/Renila luminescence intensity in SCMECs transfected with Foxo1 overexpression and Smad7 promoter plasmid and further treated with OPN or/and Sar. (n=3, mean ± SD, one-way ANOVA, Tukey's multiple comparisons) H) Western blot images and quantitative analysis showing the relative protein expression levels of p-Smad2/3 to Smad2/3, p-mTOR to mTOR, and p-GSK3β to GSK3β in different treatment group of SCMECs with OPN or/and Sar. (n=3, mean ± SD, one-way ANOVA, Tukey's multiple comparisons) ns not significant, ∗ p < 0.05, ∗∗ p < 0.01, ∗∗∗ p < 0.001, ∗∗∗∗ p < 0.0001


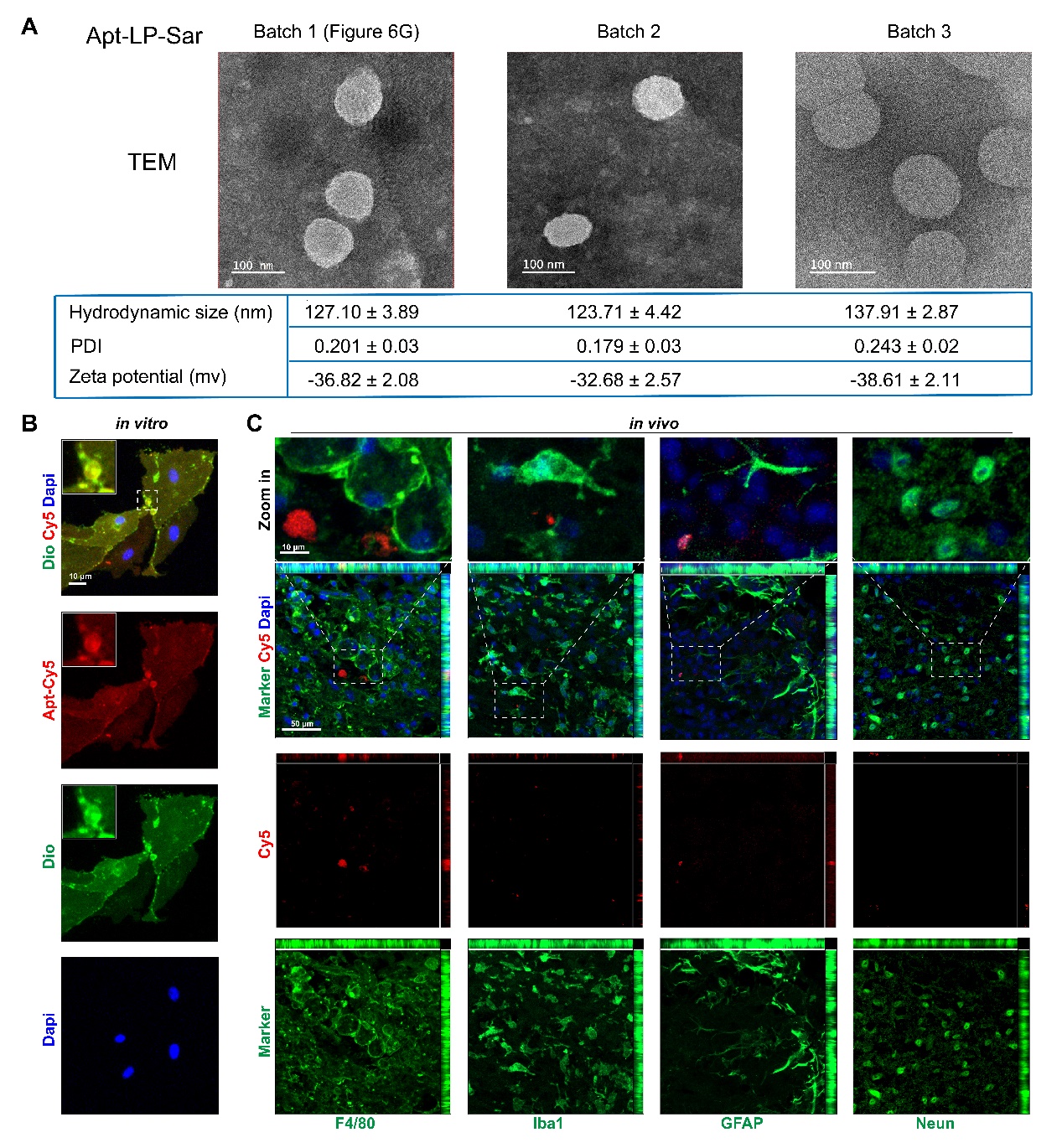
**Figure S9.** A) Representative TEM images illustrating the morphology and dispersity of Aptamer-liposome encapsulated sarmentosin (Apt-LP@Sar) in three bathes. (Scale bar = 100 nm) B) Representative confocal images demonstrating that after treatment with Dio-labeled Apt (Cy5)-LP@Sar, SCMECs exhibit co-localization of Dio (green) and Cy5 (red). C) Representative confocal images showing the localization of Apt (Cy5)-LP@Sar with macrophages (F4/80), microglia (Iba1), astrocytes (GFAP), and neurons (Neun). The localization of Apt (Cy5)-LP@Sar with endothelial cells (CD31) and quantitative analysis is presented in Figure 6L.


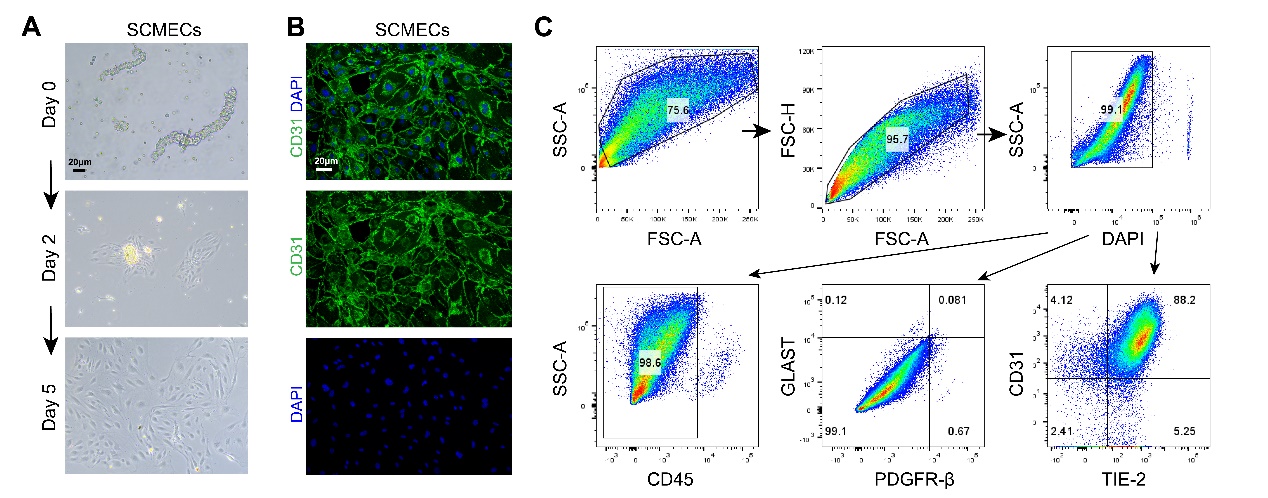
**Figure S10.** A) Light microscopy images showing the morphology of SCMECs cultured in vitro (Scale bar = 20 μm). B) IF images of SCMECs cultured in vitro (CD31: green, DAPI: blue, Scale bar = 20 μm). C) Flow cytometry results for SCMECs cultured in vitro show that 98.6% of viable cells are CD45^-^, 99.1% are GLAST^-^, and PDGFR-β^-^, 92.32% of viable cells are CD31^+^, and 93.45% are TIE-2^+^.


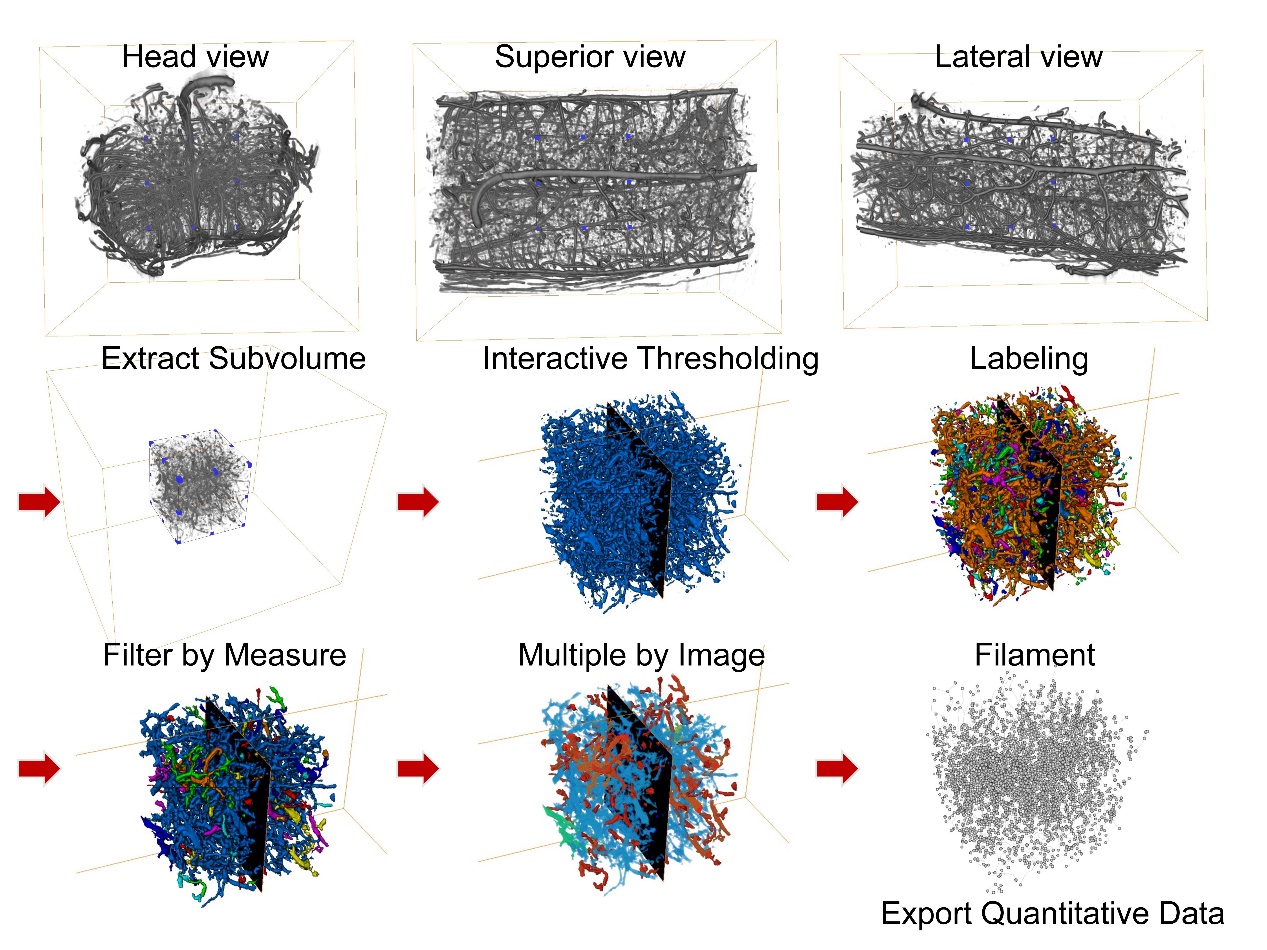
**Figure S11.** SRμCT 3D data processing steps: 1) Obtain a 3D image through volume rendering; 2) Extract a 600 × 600 × 600 voxel subvolume from either the sham or injury group spinal cord center; 3) Perform interactive thresholding on each xy plane, selecting a uniform CT threshold for all data; 4) Use the Labeling function to mark all vessel segments with different colors; 5) Remove small non-vessel voxel particles using the Filter by measure; 6) Overlay the filtered dataset with the original dataset using the Multiple by Image function to complement and reconstruct details; 7) Convert the image type to 8-bit and perform Filament reconstruction and analysis to calculate the vasculature's nodes, segments, and total curved length.
