# Supplementary material for: Endothelial Foxo1 Phosphorylation Inhibition via Aptamer‐Liposome Alleviates OPN‐Induced Pathological Vascular Remodeling Following Spinal Cord Injury
Source: Adv Sci (Weinh). 2024 Sep 28;11(43):2406398. doi: 10.1002/advs.202406398 (PMC11578346; doi:10.1002/advs.202406398)
Supplement: Supplementary file 2 — Supporting Information [file ADVS-11-2406398-s004.docx]

**Expanded Methods**

***Macrophage depletion in mice*:** A mouse model with macrophage depletion was established by intraperitoneal administration of 200 μl clodronate liposomes (Yeasen) starting three days before surgery and continuing every three days post-operation until 7 dpi. The control group received control liposomes (Yeasen) using the same administration process.

***Mouse bone marrow-derived macrophage isolation and culture*:** WT or OPN KO C57BL/6 mice (8 weeks old, weighing 20–25 g) were randomly selected and euthanized after a week of acclimatization. The mice were sterilized by immersion in a 75% alcohol solution for 5–6 minutes and secured onto a surgical plate within a sterile environment. A skin incision was made in the groin area, lower limb muscles were separated, and the femur and tibia were carefully exposed and removed intact. The femur and tibia were then separated along the joint, and any remaining muscle tissue was meticulously removed. After separating both sides of the epiphysis, bone marrow cells were flushed out using a 20 mL syringe filled with Iscove's Modified Dulbecco's Medium (IMDM) (Gibco). These cells were gently blown repeatedly with a dropper, followed by the addition of red blood cell lysate to eliminate any remaining red blood cells. The cells were then centrifuged at 1500 rpm for 5 minutes. The bone marrow-derived cells were collected after discarding the supernatant and resuspended in IMDM containing 10% fetal bovine serum (FBS) (Gibco). The cells were uniformly seeded into T75 culture flasks overnight to eliminate fast-adherent mesenchymal cells. The following day, the supernatant was collected, centrifuged at 1500 rpm for 5 minutes, and the pellet cells were retained. These cells were then resuspended in an IMDM medium containing 10% FBS and 20 ng/ml of recombinant mouse macrophage colony-stimulating factor (M-CSF) (Peprotech) to promote differentiation into macrophages. After 3 - 5 days of culture, the adherent cells were identified as mouse BMDMs.

***Scratch wound migration assay*:** A cross-shaped scratch was created in the monolayer cells of bEnd.3 using a 200 μl pipette tip when the cells reached 90% confluence in a six-well plate. Images of the wound were captured at 0 h, 12 h, and 24 h after scratching using an optical microscope (Zeiss). The distance between the edges of the scratch in three randomly selected fields was measured using ImageJ. The recovery rate is obtained by calculating the ratio of the migration distance to the original distance.

***Tube formation assay*:** Prior to the experiment, precooling was performed on a 96-well plate and pipette tips. A total of 100 μl of prechilled Matrigel (Corning) was added to each well of a 96-well plate. Subsequently, bEnd.3 cells (2 × 10^4 cells/well) were seeded onto the Matrigel. The formation of capillary-like structures was assessed using an optical microscope after 12 hours of culture. The ability to construct capillary-like structures was quantified using ImageJ software, which counted the mesh numbers in five randomly chosen microscopic fields.

***Molecular Docking and Drug Screening*:**

I. Protein Preprocessing

The AlphaFold2 model was utilized to obtain the crystal structure of the FOXO1 protein. The obtained protein crystal structures underwent preprocessing using the Schrödinger software's Protein Preparation Wizard module. This included steps such as protein preprocessing, regeneration of native ligand states, optimization of H-bond assignments, protein energy minimization, and removal of water.

II. Ligand Preprocessing

The 2D sdf structure files of eight compound libraries were processed using the LigPrep module within the Schrödinger suite to generate all 3D chiral conformations for each compound.

III. ADME/T Assessment

The QikProp module was employed to predict all ADME/T parameter scores for the ligand compounds, resulting in a total of 51 parameter types. Evaluation primarily considered adherence to Lipinski's Rule of Five, with parameters including mol_MW < 500, QPlogPo/w < 5, donorHB ≤ 5, and accptHB ≤ 10. Compounds satisfying these rules were considered drug-like, with lower parameter values indicating better drug-likeness.

A total of 35,143 ligand compounds from eight compound libraries were subjected to ADME/T assessment, revealing 19,486 compounds with higher potential for clinical development. Subsequent HTVS screening was conducted on these compounds.

IV. Identification of Active Sites

The SiteMap module within the Schrödinger suite was employed to predict the optimal binding sites. Subsequently, the Receptor Grid Generation module was used to set the most suitable enclosing box that perfectly enveloped the predicted binding sites, facilitating the identification of active pockets on the protein.

V. Molecular Docking

Each processed ligand from the eight compound libraries was sequentially docked with the active site of the FOXO1 protein using molecular docking techniques (HTVS, SP, and XP) with increasing docking precision. Lower docking scores indicated lower binding free energy and higher binding stability.

VI. MM-GBSA Analysis

The lowest-scoring ligand from the molecular docking was subjected to MM-GBSA calculations to analyze the binding free energy. The MM-GBSA dG Bind value approximates the small molecule's binding free energy to the protein, with lower values indicating higher stability of ligand binding to FOXO1.

Results from ADME/T, HTVS, SP, XP, and MM-GBSA analyses are available upon request.
