# Supplementary material for: Endothelial Foxo1 Phosphorylation Inhibition via Aptamer‐Liposome Alleviates OPN‐Induced Pathological Vascular Remodeling Following Spinal Cord Injury
Source: Adv Sci (Weinh). 2024 Sep 28;11(43):2406398. doi: 10.1002/advs.202406398 (PMC11578346; doi:10.1002/advs.202406398)
Supplement: Supplementary file 3 — Supporting Information [file ADVS-11-2406398-s005.docx]

**Major Resources Table**

**Animal (in vivo studies)**

| **Species** | **Vendor or Sources** | **Background Strain** | **Sex** | **Persistent ID** |
| --- | --- | --- | --- | --- |
| Mouse | Hunan SJA Laboratory Animal Co.，Ltd | C57BL/6J | Male/Female | RRID: IMSR_JAX:000664 |

**Genetically Modified Animals**

| **Species** | **Vendor or Sources** | **Background Strain** | **Other Information** | **Persistent ID** |
| --- | --- | --- | --- | --- |
| Mouse | Jackson laboratory | C57BL/6J | B6.129S6(Cg)-Spp1tm1Blh/J  OPN KO | RRID: IMSR_JAX:004936 |

**Antibodies**

| **Target Antigen** | **Vendor or Sources** | **Catalog#** | **Working Concentration** | **Persistent ID** |
| --- | --- | --- | --- | --- |
| Mouse myeloma cell line NS0-derived recombinant mouse CD31/PECAM-1  Glu18-Lys590 | R&D systems | FAB3628G | IF 1:400 | RRID: AB_10972784 |
| GFAP fusion protein Ag10423 | Proteintech | 16825-1-AP | IF 1:800 | RRID: AB_2109646 |
| Synthetic peptide corresponding to Human GFAP aa 400 to the C-terminus (C terminal) (Cysteine residue) | Abcam | ab53554 | IF 1:800 | RRID: AB_880202 |
| Collagen Type III (N-terminal) fusion protein Ag18658 | Proteintech | 22734-1-AP | IF 1:400 WB 1:1000 | RRID: AB_2879158 |
| Human recombinant ZO-1 fusion protein encompassing amino acids 334-634 | Thermo Fisher | 33-9100 | IF 1:200  WB: 1:2000 | RRID: AB_87181 |
| Synthetic peptide corresponding to Human alpha smooth muscle Actin (N terminal) | Abcam | ab5694 | IF 1:400 | RRID: AB_2223021 |
| Osteopontin fusion protein Ag19216 | Proteintech | 22952-1-AP | IF 1:400  WB 1:2000 | RRID: AB_2783651 |
| C57BL/10 mouse splenic T cells and concanavalin A-activated C57BL/10 splenocytes (CD11b) | Proteintech | FITC-65055 | IF 1:400 | RRID: AB_2883745 |
| Recombinant protein mapping to amino acids 1-122 representing full length Apelin of human origin | Santa Cruz | sc-293441 | IF 1:100  WB 1:500 | RRID: AB_3083487 |
| Angiopoietin 2 fusion protein Ag17938 | Proteintech | 24613-1-AP | WB 1:2000 | RRID: AB_2879639 |
| Recombinant protein to integrin alpha V | Abcam | ab179475 | IF 1:500 | RRID: AB_2716738 |
| Synthetic peptide to Ki67 | Abcam | ab15580 | IF 1:400 | RRID: AB_443209 |
| Nitric oxide synthase 2, inducible | Proteintech | 18985-1-AP | WB 1:2000 | RRID: AB_2782960 |
| Recombinant protein Beta Actin | Proteintech | 66009-1-Ig | WB 1:10000 | RRID: AB_2687938 |
| Carrier-protein conjugated synthetic peptide encompassing a sequence within the N-terminus region of human VE-Cadherin | GeneTex | GTX132982 | WB 1:2000 | RRID: AB_2886797 |
| Carrier-protein conjugated synthetic peptide encompassing a sequence within the C-terminus region of human N-Cadherin | GeneTex | GTX127345 | WB 1:2000 | RRID: AB_2885644 |
| Collagen Type I fusion protein Ag6281 | Proteintech | 14695-1-AP | WB 1:2000 | RRID: AB_2082037 |
| Synthetic peptide within Human Claudin 5 aa 200 to the C-terminus (intracellular) | Abcam | ab131259 | WB 1:2000 | RRID: AB_11157940 |
| A synthetic peptide corresponding to residues surrounding Val294 of human occludin protein | Cell Signaling Technology | 91131 | WB 1:1000 | RRID: AB_2934013 |
| FOXO1 fusion protein Ag13296 | Proteintech | 18592-1-AP | WB 1:1000  IP 1ug | RRID: AB_10860103 |
| A synthetic phosphopeptide corresponding to residues around Ser256 of human Fox01 | Cell Signaling Technology | 9461 | WB 1:1000 | RRID: AB_329831 |
| Histone-H3 fusion protein Ag10644 | Proteintech | 17168-1-AP | WB 1:5000 | RRID: AB_2716755 |
| SMAD7 fusion protein Ag13688 | Proteintech | 25840-1-AP | WB 1:1000 | RRID: AB_2848137 |
| Synthetic peptide within Human Smad2 + Smad3 aa 30-80 conjugated to keyhole limpet haemocyanin | Abcam | ab217553 | WB 1:1000 | RRID: AB_3073970 |
| Synthetic peptide corresponding to Human Smad2 + Smad3 (phospho T8) | Abcam | ab272332 | WB 1:1000 | RRID: AB_3073969 |
| Tissue, cells or virus corresponding to F4/80 | Abcam | ab6640 | IF 1:400 | RRID: AB_1140040 |
| Synthetic peptide to beta III Tubulin | Abcam | ab18207 | IF 1:400 | RRID: AB_444319 |
| Carrier-protein conjugated synthetic peptide encompassing a sequence within the C-terminus region of human Iba1 | GeneTex | GTX100042 | IF 1:400 | RRID: AB_1240434 |
| Synthetic peptide to Neun | Abcam | ab177487 | IF 1:400 | RRID: AB_2532109 |
| AKT fusion protein Ag0213 | Proteintech | 10176-2-AP | WB 1:2000 | RRID: AB_2224574 |
| Peptide to Phospho-AKT (Ser473) | Proteintech | 80455-1-RR | WB 1:4000 | RRID: AB_2918892 |
| Rabbit IgG control | Proteintech | 30000-0-AP | IP 1ug | RRID: AB_2819035 |
| Total mTOR protein | Cell Signaling Technology | 2983T | WB 1:1000 | RRID: AB_2105622 |
| Ser2448 of mTOR protein | Cell Signaling Technology | 5536T | WB 1:1000 | RRID: AB_10691552 |
| Total GSK3 beta | Affinity | AF5016 | WB 1:1000 | RRID: AB_2834935 |
| Phospho-GSK3 beta (Ser9) | Affinity | AF2016 | WB 1:1000 | RRID: AB_2834439 |
| FITC anti-mouse CD45 Antibody | Biolegend | 157214 | IF 1:200 | RRID: AB_2894427 |
| GLAST (ACSA-1) Antibody， PE | Miltenyi Biotec | 130-118-483 | IF 1:200 | RRID: AB_2811532 |
| CD140b Antibody, anti-mouse, PE-Vio 770 | Biolegend | 130-123-573 | IF 1:200 | RRID: AB_2928251 |
| Alexa Fluor 700 anti-mouse CD31 Antibody | Biolegend | 102444 | IF 1:200 | RRID: AB_2832289 |
| APC anti-mouse CD202b (Tie-2, CD202) Antibody | Biolegend | 124010 | IF 1:200 | RRID: AB_10897106 |
| Donkey Anti-Goat IgG H&L (Alexa Fluor® 488) | Abcam | ab150129 | IF 1:400 | RRID: AB_2687506 |
| Donkey Anti-Mouse IgG H&L (Alexa Fluor® 594) | Abcam | ab150108 | IF 1:400 | RRID: AB_2732073 |
| Donkey Anti-Rabbit IgG H&L (Alexa Fluor® 594) | Abcam | ab150076 | IF 1:400 | RRID: AB_2782993 |
| Donkey Anti-Rat IgG H&L (Alexa Fluor® 488) | Abcam | ab150153 | IF 1:400 | RRID: AB_2737355 |
| Donkey Anti-Rabbit IgG H&L (Alexa Fluor® 647) | Abcam | ab150075 | IF 1:400 | RRID: AB_2752244 |
| Donkey Anti-Rabbit IgG H&L (Alexa Fluor® 488) | Abcam | ab150073 | IF 1:400 | RRID: AB_2636877 |
| Donkey Anti-Rat IgG H&L (Alexa Fluor® 594) | Abcam | ab150156 | IF 1:400 | RRID: AB_2890252 |
| Goat Anti-Rabbit IgG(H+L)(peroxidase/HRP conjugated) | Elabscience | E-AB-1003 | WB 1:10000 | RRID: AB_2921220 |
| Goat Anti-Mouse IgG(H+L)(peroxidase/HRP conjugated) | Elabscience | E-AB-1001 | WB 1:10000 | RRID: AB_2715613 |

**Cultured Cells**

| **Name** | **Vendor or Sources** | **Persistent ID** |
| --- | --- | --- |
| Primary SCMECs | Laboratory isolation | N/A |
| Primary BMDMs | Laboratory isolation | N/A |
| bEnd.3 | Procell | CL-0598 |

**Other Reagents**

| **Description** | **Source/Repository** | **Persistent ID** |
| --- | --- | --- |
| Evans blue | Sigma Aldrich | E2129 |
| MICROFIL | Flow Tech inc | mv-112 |
| Methyl Salicylate | Macklin | M813577 |
| Paraformaldehyde,4％ | Solarbio | P1110 |
| Fetal Bovine Serum | Gibco | A5669401 |
| Dulbecco's Modified Eagle Medium (high glucose) | Gibco | 11965092 |
| Dulbecco's Modified Eagle Medium (low glucose) | Gibco | 11885084 |
| Iscove's Modified Dulbecco's Medium (IMDM) | Gibco | 12440053 |
| Collagenase Type II | Sigma Aldrich | C2-BIOC |
| Collagenase/dispase | Sigma Aldrich | COLLDISP-RO |
| Coating collagen | Sigma Aldrich | 122-20 |
| Heparin sodium | Selleckchem | S1346 |
| Puromycin 2HCl | Selleckchem | S7417 |
| Penicillin-Streptomycin | Gibco | 15070063 |
| Recombinant Murine FGF-basic | Peprotech | 450-33 |
| Recombinant Murine M-CSF | Peprotech | 315-02 |
| Recombinant Murine IFN gamma | Peprotech | 315-05 |
| Recombinant Mouse TGF-beta 1 Protein | R&D systems | 7666-MB |
| Recombinant Mouse Osteopontin/OPN Protein | R&D systems | 441-OP |
| Cilengitide | MedChemExpress | HY-16141 |
| Wortmannin | MedChemExpress | HY-10197 |
| GoScript™ Reverse Transcription System | Promega Corporation | A5003 |
| Mouse Direct PCR Kit (For Genotyping) | Bimake | B40013 |
| 2x SYBR Green qPCR master mix | Bimake | B21203 |
| TRIzol™ Plus RNA Purification Kit | Invitrogen | 12183555CN |
| BCA Protein Colorimetric Assay Kit | Elabscience | E-BC-K318-M |
| Antibody Dilution Buffer | Elabscience | E-IR-R106 |
| RIPA Lysis Buffer (Strong) | Elabscience | E-BC-R327 |
| Nuclear and Cytoplasmic Protein Extraction Kit | Beyotime | P0027 |
| SDS-PAGE Sample Loading Buffer | Beyotime | P0015L |
| Protease inhibitor cocktail for general use | Beyotime | P1005 |
| Basic ECL reagents | ShareBio | SB-WB001 |
| Surcose | BioFroxx | 1245 |
| Albumin Bovine (BSA) | BioFroxx | 4240 |
| Triton x-100 | Solarbio | T8200 |
| TBST buffer | Solarbio | T1081 |
| Tween-20 | Solarbio | T8220 |
| Fluoroshiel with DAPI | GeneTex | GTX30920 |
| Fluorescein isothiocyanate (FITC)-dextran | Sigma Aldrich | FD40 |
| Mouse Foxo1(NM_019739) plasmid  (CMV enhancer-MCS-3FLAG-SV40-Puromycin) | GeneChem | GOSE0386296 |
| Mouse Smad7(NM_001042660-promoter) plasmid  (MCS-firefly_Luciferase) | GeneChem | GOSE0386297 |
| Dual-Luciferase Reporter Assay System | Promega Corporation | E1910 |
| BeyoChIP™ ChIP Assay Kit | Beyotime | P2080S |
| DNA Purification Kit | Beyotime | D0033 |
| Pyrocatechol monoglucoside | BioBioPha | BBP02257 |
| Sarmentosin | BioBioPha | BBP00568 |
| Torachrysone 8-O-glucoside | BioBioPha | BBP02085 |
| Robtein | BioBioPha | BBP02380 |
| 3-O-Methylquercetin | BioBioPha | BBP00421 |
| Secodihydro-hydramicromelin B | BioBioPha | BBP02594 |
| Dihydrorobinetin | BioBioPha | BBP01845 |
| Cell Counting Kit-8 | Beyotime | C0038 |
| DiO | Beyotime | C1038 |
| DiR | Invitrogen | D12731 |
| Dextran | Invitrogen | D22910 |
| Clodronate liposomes | Yeasen | 40337ES |
| Control liposomes | Yeasen | 40338ES |
| Matrigel | Corning | 356234 |
| Aspirin | MCE | HY-14654 |
| Lipofectamin 2000 CD | Invitrogen | 12566014 |
| Cell Staining Buffer | Biolegend | 420201 |
