# Supplementary material for: Endothelial Foxo1 Phosphorylation Inhibition via Aptamer‐Liposome Alleviates OPN‐Induced Pathological Vascular Remodeling Following Spinal Cord Injury
Source: Adv Sci (Weinh). 2024 Sep 28;11(43):2406398. doi: 10.1002/advs.202406398 (PMC11578346; doi:10.1002/advs.202406398)
Supplement: Supplementary file 4 — Supporting Information [file ADVS-11-2406398-s001.docx]

Table S1. The primer pairs used for genotyping and quantitative real-time PCR

| Gene | Direction | Sequence (5’-3’) |
| --- | --- | --- |
| OPN full-length | forward | GTGACTTGGTGGTGATCTAGTG |
|  | reverse | AGATCAGAGGACTACAACAGAC |
| OPN intermediate | forward | CCAGTCACTCAAGGTCCTGT |
|  | reverse | CTGAAGAATGTTCGCAGC |
| Smad7 | forward | GGCCGGATCTCAGGCATTC |
|  | reverse | TTGGGTATCTGGAGTAAGGAGG |
| Gapdh | forward | AGGTCGGTGTGAACGGATTTG |
|  | reverse | TGTAGACCATGTAGTTGAGGTCA |
| Smad7 promoter1 | forward | GTACCTTCCTCCGCGGAAAC |
|  | reverse | CAATTCCAGACCAACGGGGG |
| Smad7 promoter2 | forward | CGAACCCATCCGGTGACTG |
|  | reverse | ACTCCAGATCCCCCATTCCT |
| Smad7 promoter3 | forward | GCCCTTTGGAATGTGTGGTC |
|  | reverse | GGCCACCCCCATGCTTTTA |
