# Supplementary material for: Endothelial Foxo1 Phosphorylation Inhibition via Aptamer‐Liposome Alleviates OPN‐Induced Pathological Vascular Remodeling Following Spinal Cord Injury
Source: Adv Sci (Weinh). 2024 Sep 28;11(43):2406398. doi: 10.1002/advs.202406398 (PMC11578346; doi:10.1002/advs.202406398)

ADVANCED SCIENCE Open Access

Supporting Information

for *Adv. Sci.*, DOI 10.1002/advs.202406398

Endothelial Foxo1 Phosphorylation Inhibition via Aptamer-Liposome Alleviates OPN-Induced Pathological Vascular Remodeling Following Spinal Cord Injury

Jiaqi Xu, Chaoran Shi, Yinghe Ding, Tian Qin, Chengjun Li, Feifei Yuan, Yudong Liu, Yong Xie, Yiming Qin, Yong Cao, Tianding Wu, Chunyue Duan, Hongbin Lu, Jianzhong Hu* and Liyuan Jiang**

Figure 2A

PageRuler™ Plus 10-250 kDa 26619

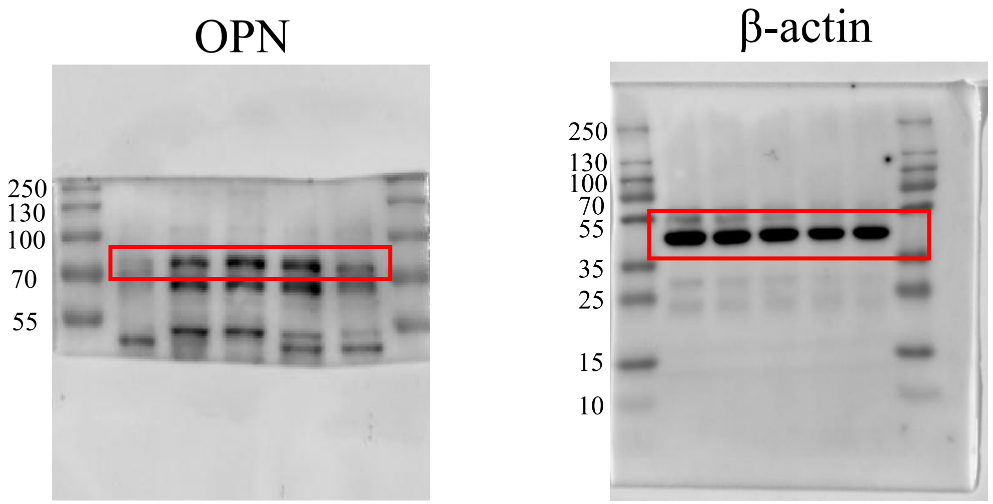

Figure 4D

PageRuler™ Plus 10-180 kDa 26616

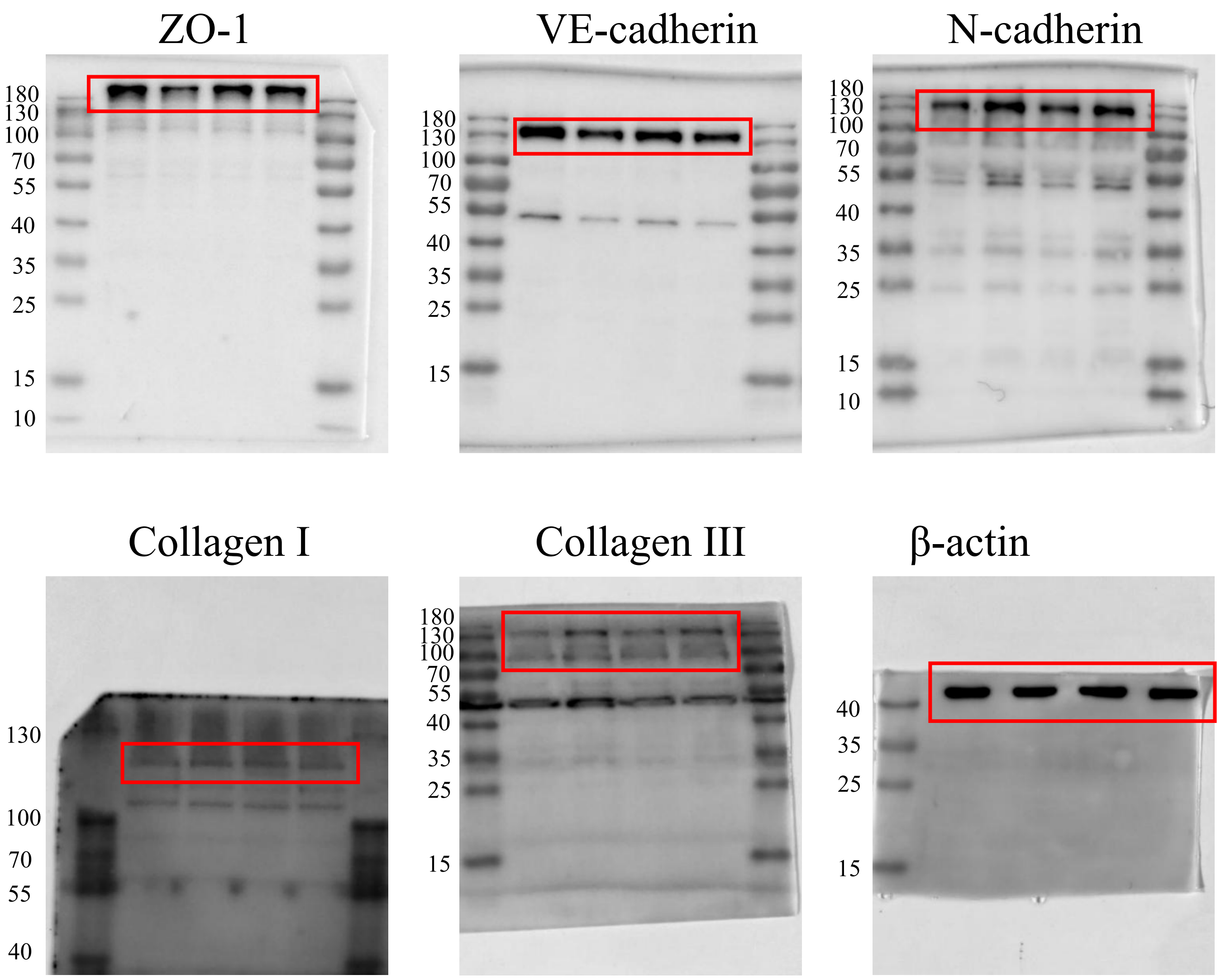

Figure 4F

PageRuler™ Plus 10-180 kDa 26616

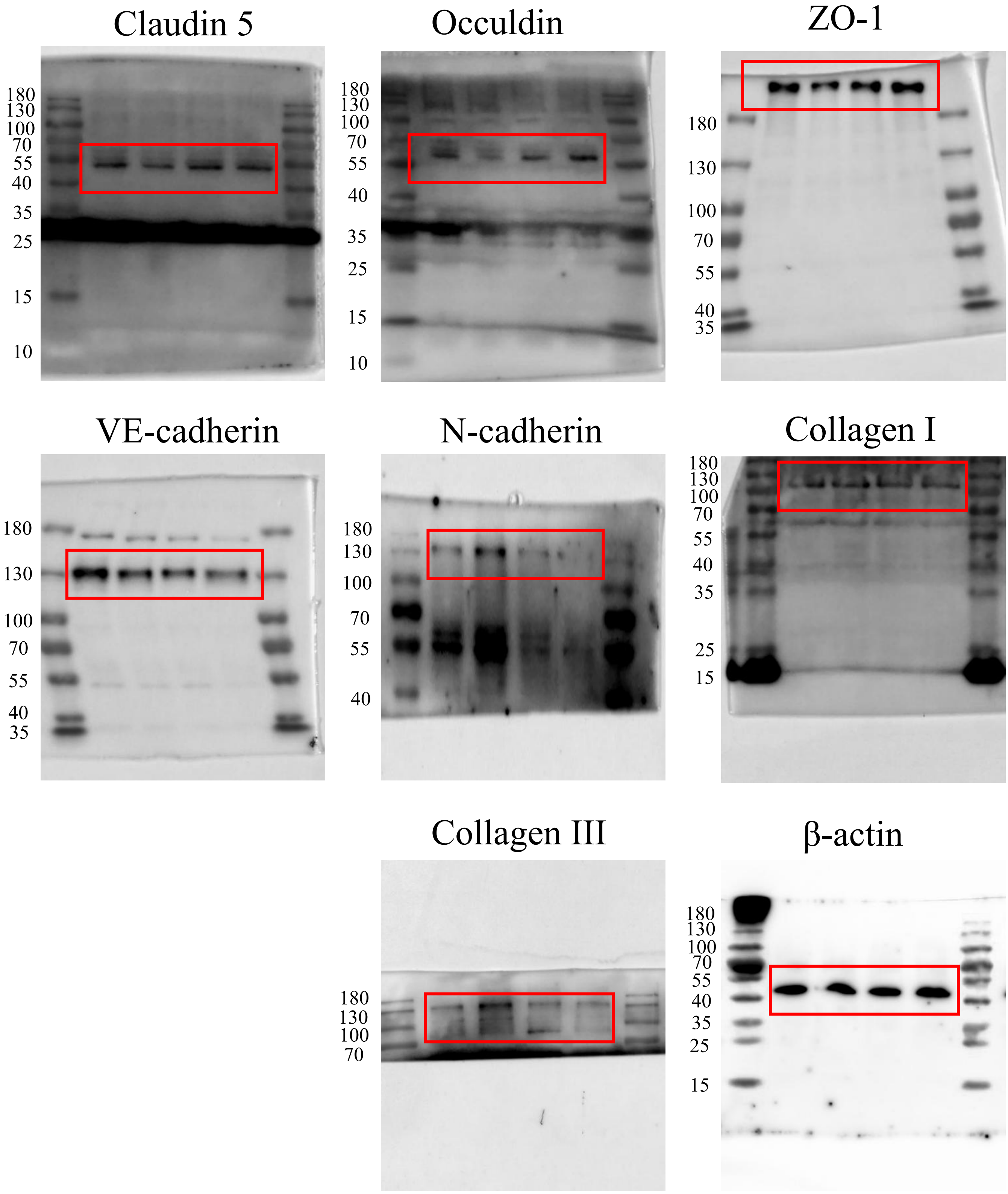

Figure 5E

PageRuler™ Plus 10-180 kDa 26616

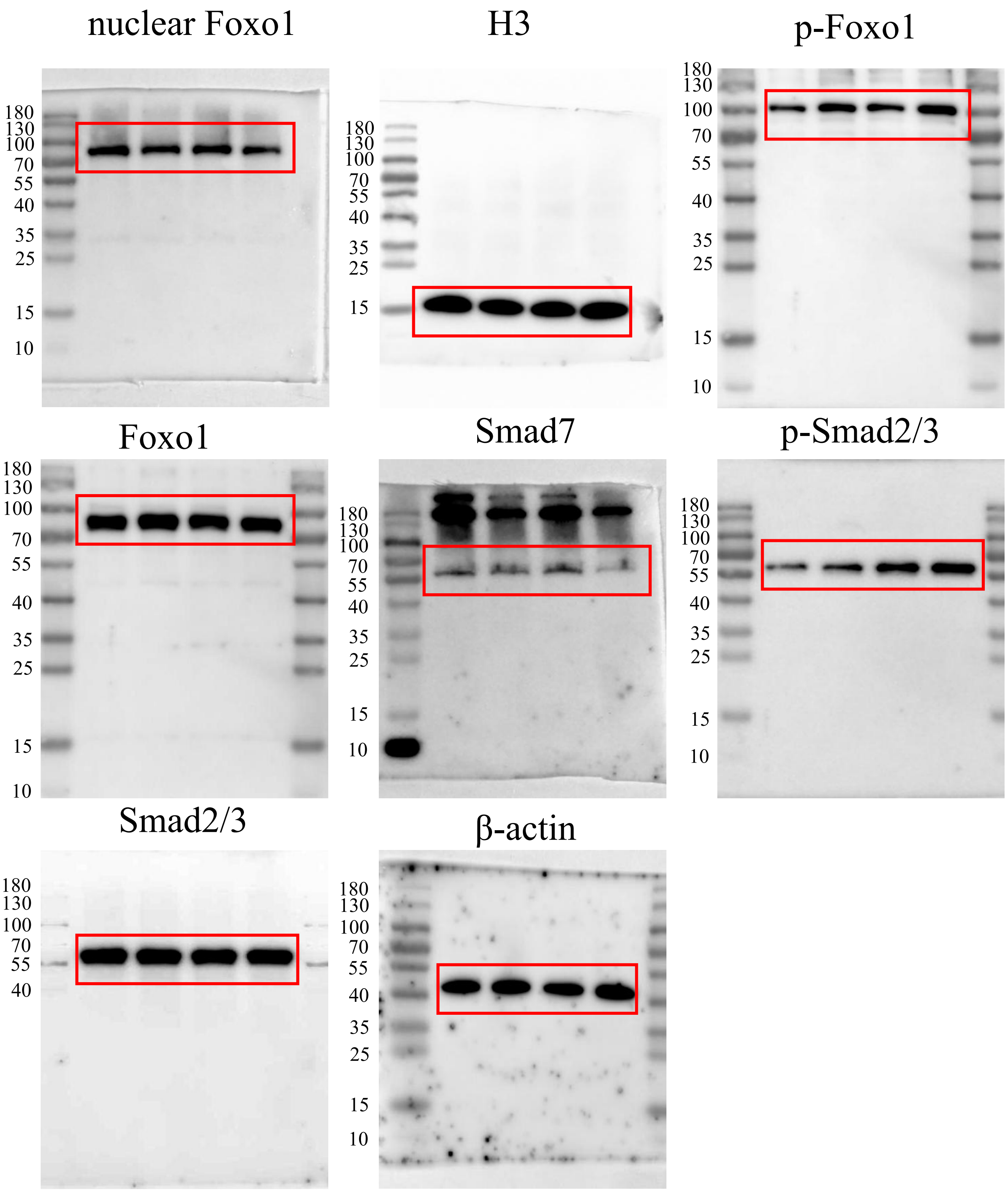

Figure 5H

PageRuler™ Plus 10-180 kDa 26616

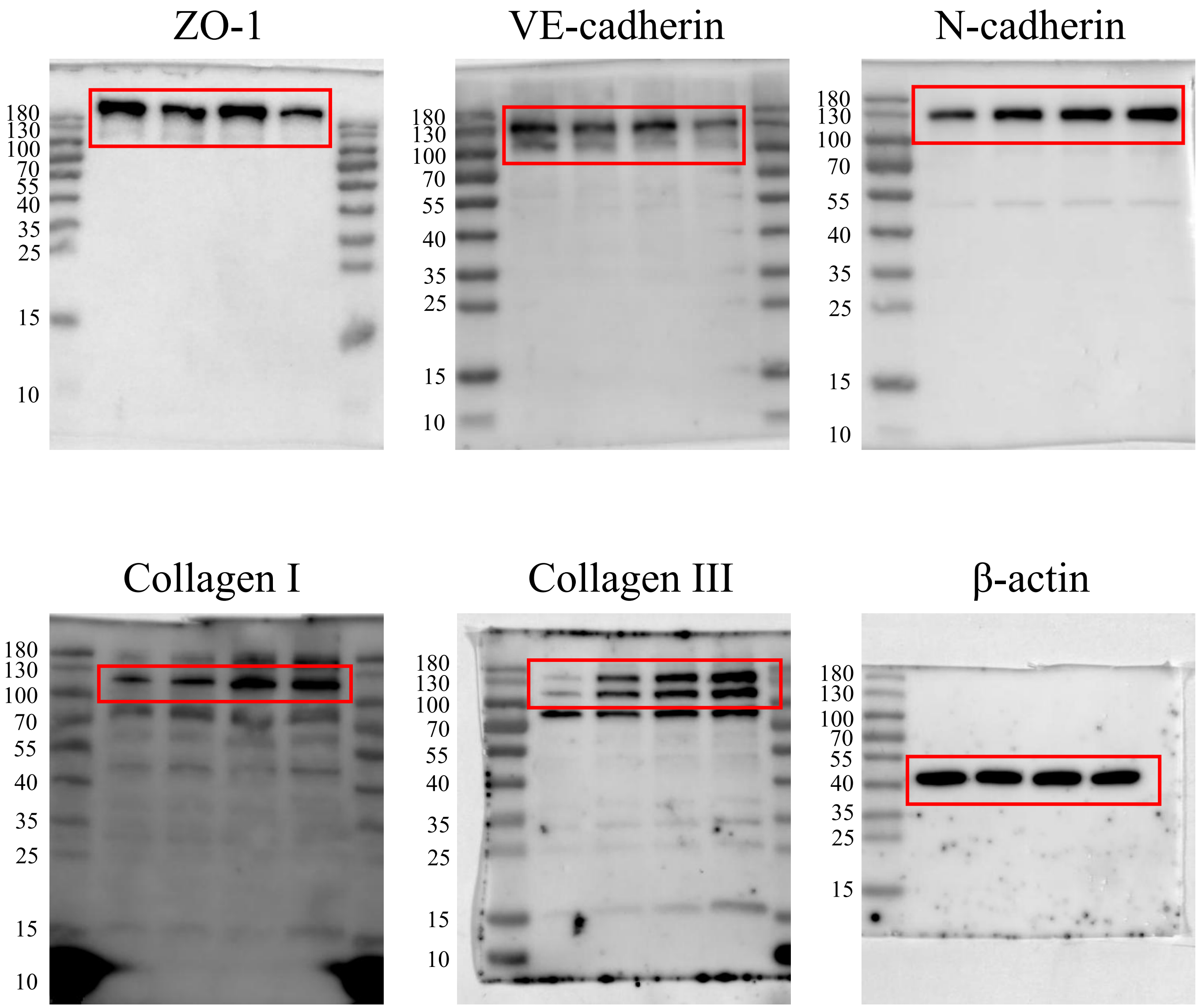

Figure 6D

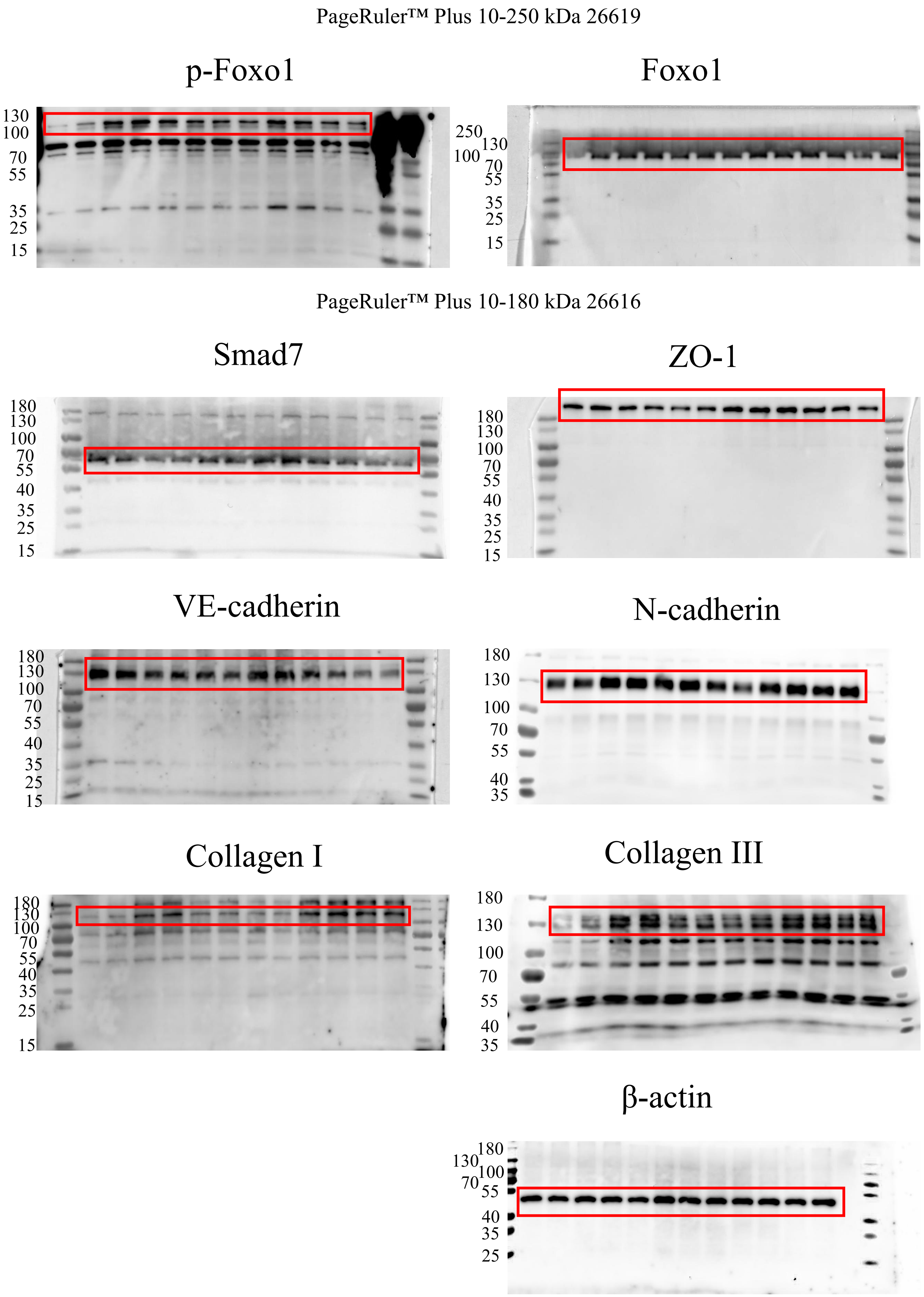
PageRuler™ Plus 10-250 kDa 26619
p-Foxo1
130 100 70 55 35 25 15
Foxo1
250 130 100 70 55 35 25 15
PageRuler™ Plus 10-180 kDa 26616
Smad7
180 130 100 70 55 40 35 25 15
ZO-1
180 130 100 70 55 40 35 25 15
VE-cadherin
180 130 100 70 55 40 35 25 15
N-cadherin
180 130 100 70 55 40 35
Collagen I
180 130 100 70 55 40 35 25 15
Collagen III
180 130 100 70 55 40 35
β-actin
180 130 100 70 55 40 35 25

Figure S4C

PageRuler™ Plus 10-250 kDa 26619

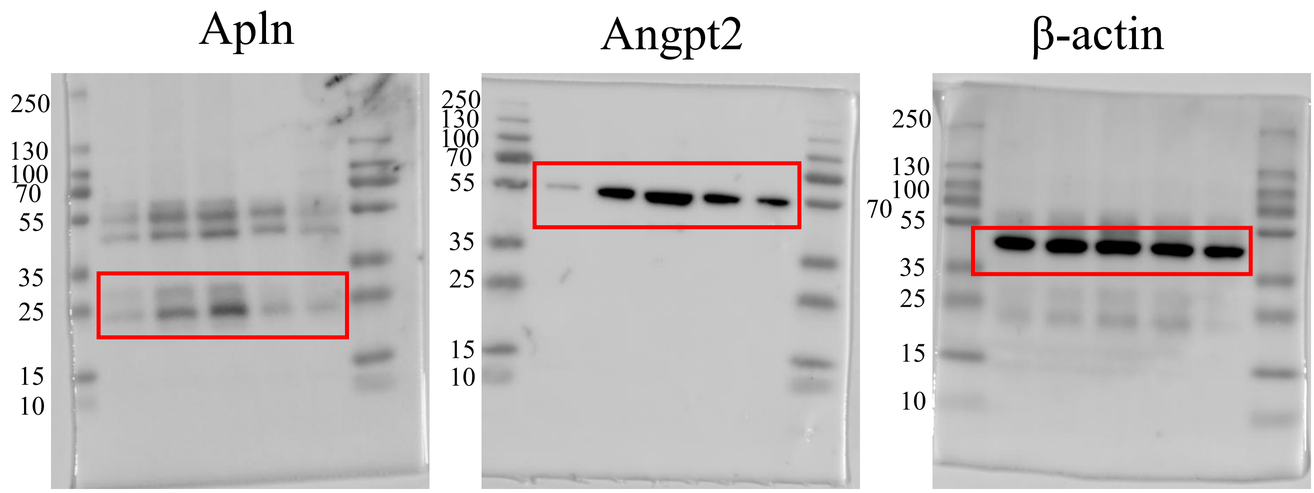

Figure S7A

PageRuler™ Plus 10-250 kDa 26619

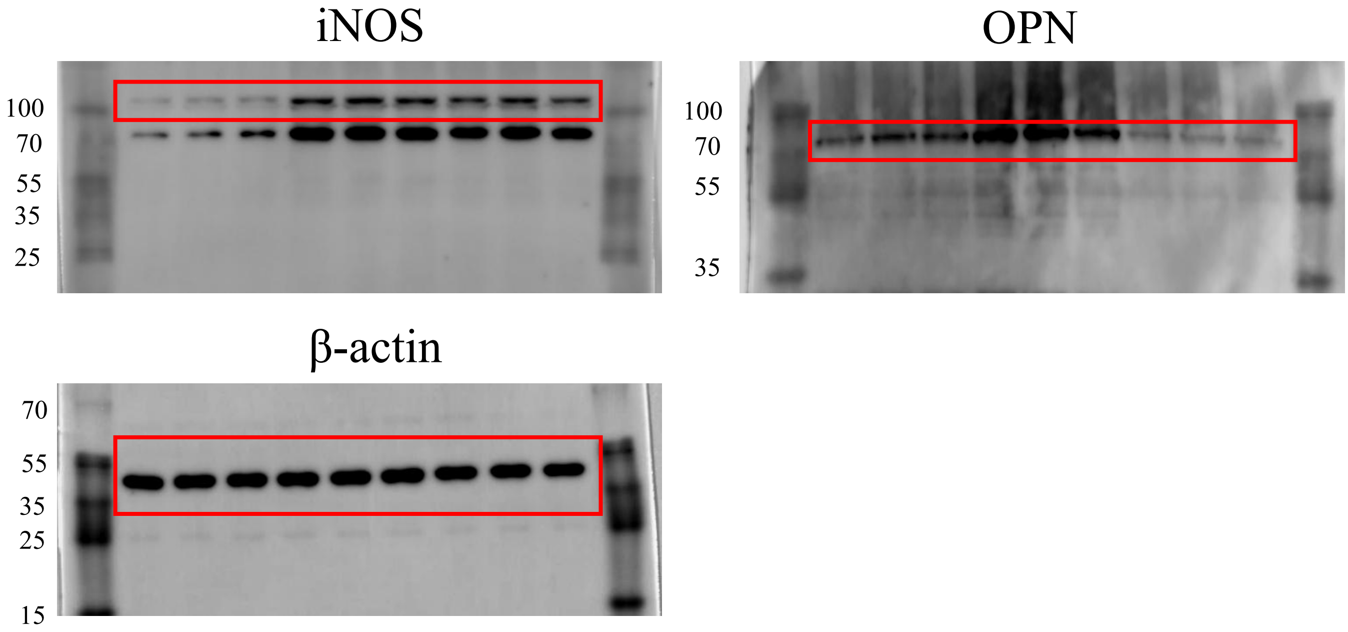

Figure S7C

PageRuler™ Plus 10-180 kDa 26616

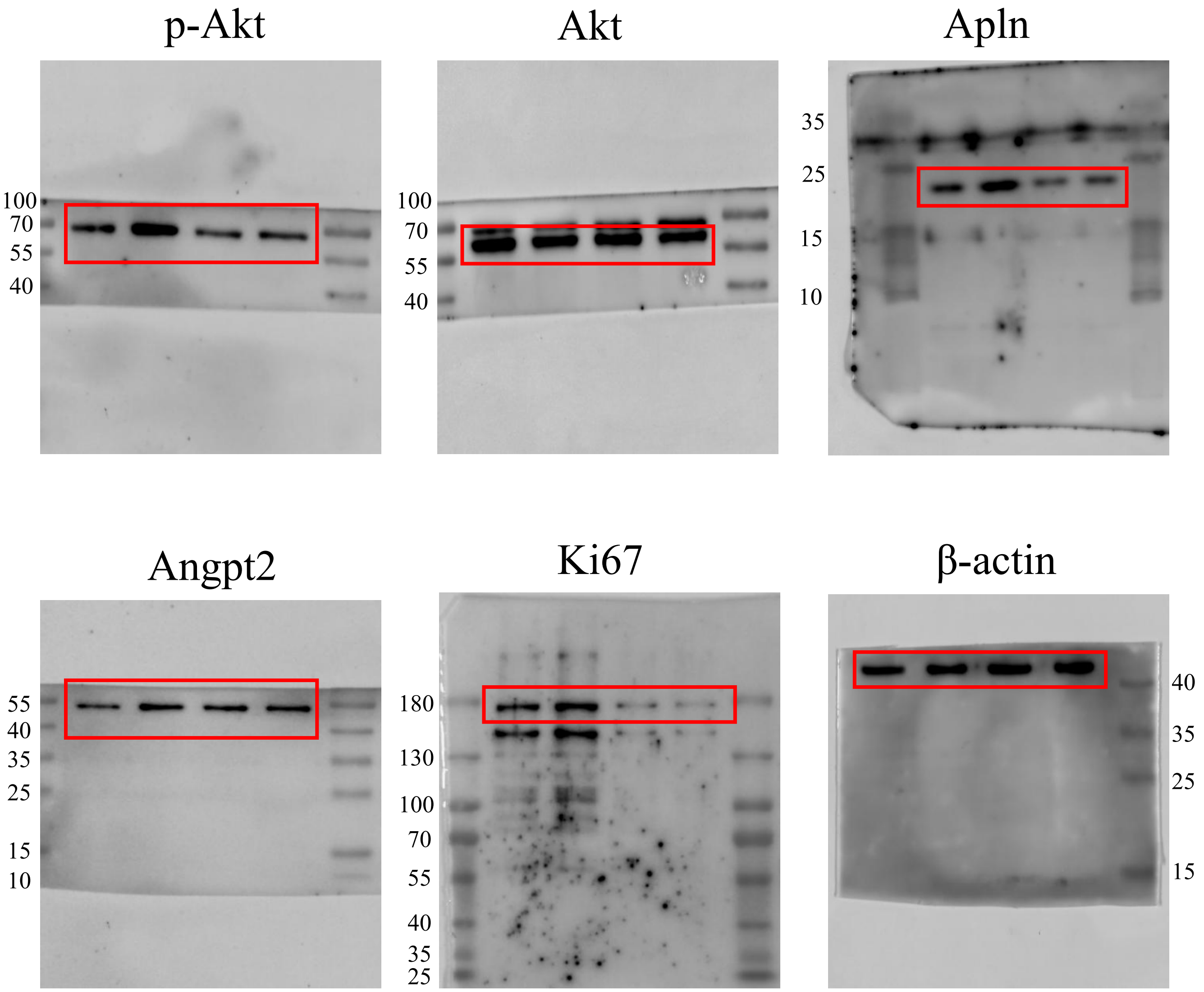

Figure S8E

PageRuler™ Plus 10-180 kDa 26616

Foxo1

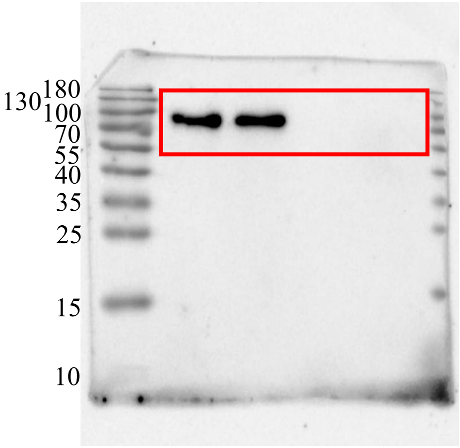

Smad7

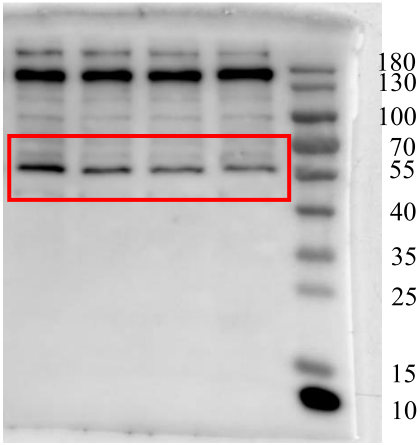

p-Smad2/3

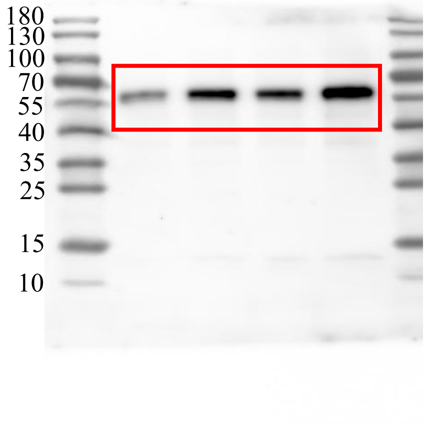

Smad2/3

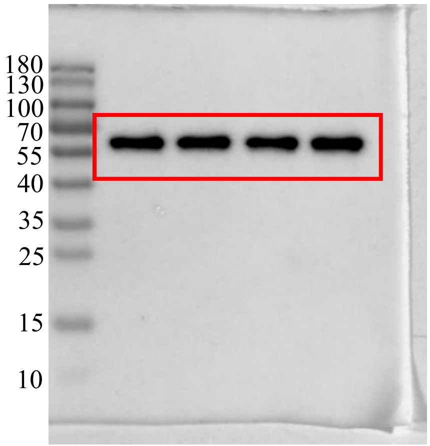

β-actin

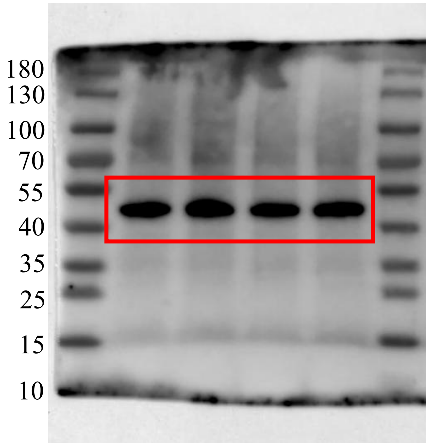

Figure S8H

PageRuler™ Plus 10-180 kDa 26616

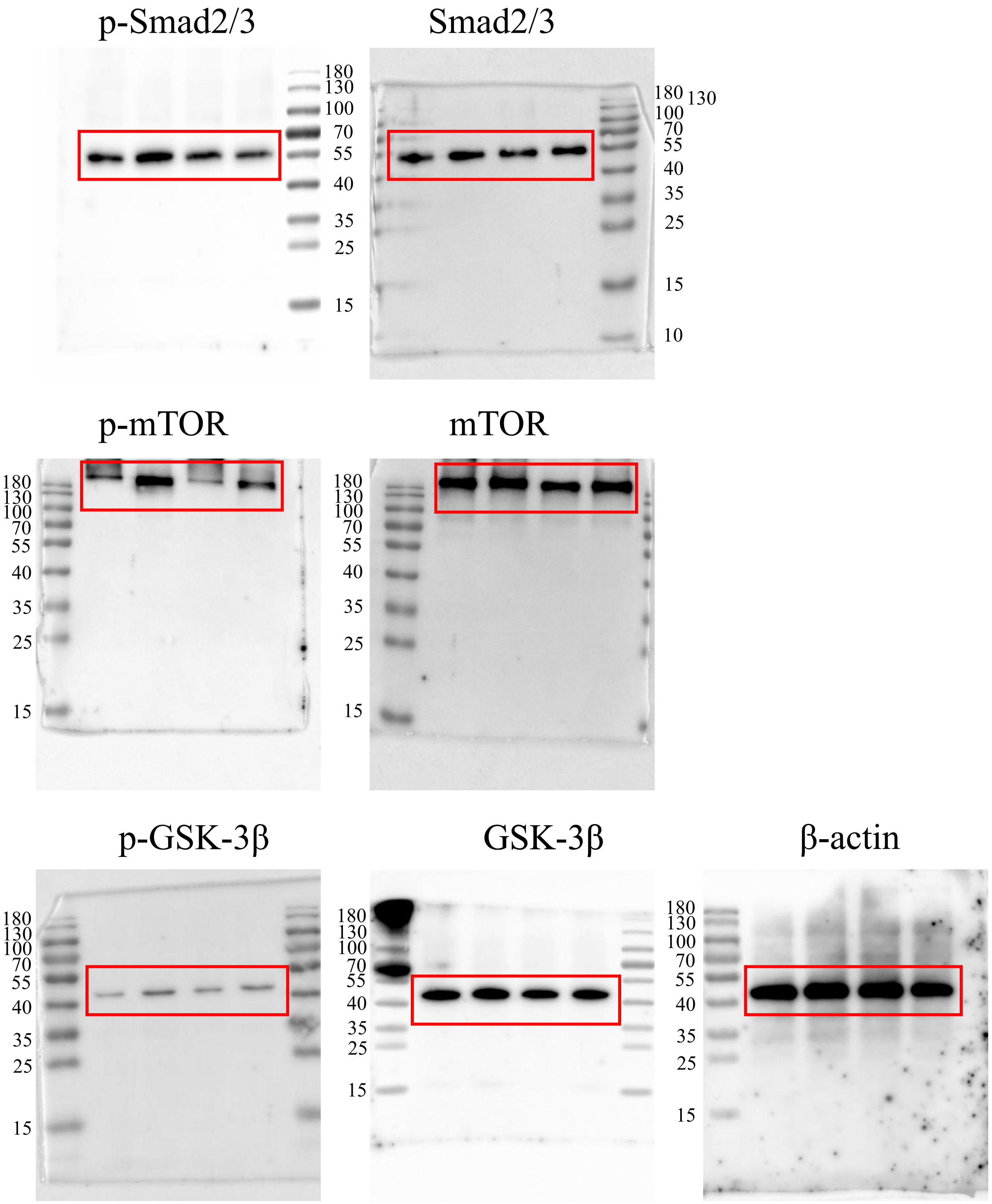

Supplement: Supplementary file 5 — Supporting Information [file ADVS-11-2406398-s002.pdf]
